# Discovery of SARS-CoV-2 main protease inhibitors using a synthesis-directed *de novo* design model†

**DOI:** 10.1039/d1cc00050k

**Published:** 2021-05-06

**Authors:** Aaron Morris, William McCorkindale, The COVID Moonshot Consortium, Nir Drayman, John D. Chodera, Savaş Tay, Nir London, Alpha A. Lee

**Affiliations:** PostEra Inc, 2 Embarcadero Centre San Franciso CA 94111 USA alpha.lee@postera.ai; Department of Physics, University of Cambridge CB3 0HE UK; The COVID Moonshot Consortium www.postera.ai/covid; The Pritzker School for Molecular Engineering, The University of Chicago Chicago IL USA; Computational and Systems Biology Program Sloan Kettering Institute, Memorial Sloan Kettering Cancer Center New York NY 10065 USA; Department of Organic Chemistry, The Weizmann Institute of Science Rehovot 76100 Israel

## Abstract

The SARS-CoV-2 main viral protease (M^pro^) is an attractive target for antivirals given its distinctiveness from host proteases, essentiality in the viral life cycle and conservation across coronaviridae. We launched the COVID Moonshot initiative to rapidly develop patent-free antivirals with open science and open data. Here we report the use of machine learning for *de novo* design, coupled with synthesis route prediction, in our campaign. We discover novel chemical scaffolds active in biochemical and live virus assays, synthesized with model generated routes.

## Abstract 2

We discovered potent SARS-CoV-2 main protease inhibitors using synthesis-directed molecular design.

## Graphical abstract

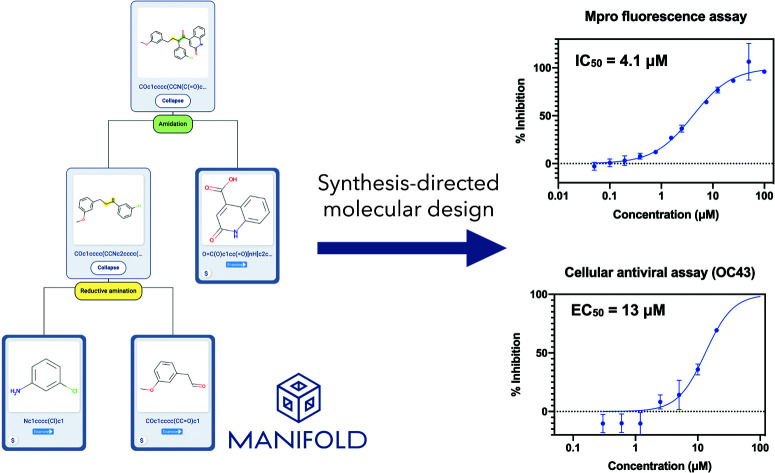

Coronaviruses are a family of pathogens that is frequently associated with serious and highly infectious human diseases, from the common cold to the SARS-CoV pandemic (2003, 774 deaths, 11% fatality rate), MERS-CoV pandemic (2012, 858 deaths, 34% fatality rate) and most recently the COVID-19 pandemic (ongoing pandemic, 1.7 million deaths up to Dec 2020). The main protease (M^pro^) is one of the best characterized drug targets for direct-acting antivirals.^1,2^ M^pro^ is essential for viral replication and its binding site is distinct from known human proteases, thus inhibitors are unlikely to be toxic.^3,4^ Moreover, the high degree of conservation across different coronaviruses renders M^pro^ targeting a fruitful avenue towards pan-cornavirus antivirals.^5^ To date, most reported M^pro^ inhibitors are peptidomimetics, covalent, or both.^2^ Peptidomimetics are challenging to develop into oral therapeutics, and covalent inhibitors incur additional idiosyncratic toxicity risks. We launched the COVID Moonshot consortium in March 2020, aiming to find oral antivirals against COVID-19 in an open-science, patent-free manner.^6^

Here we report the prospective use of a simple model to rapidly expand hits. Starting from 42 compounds with IC_50_ within assay dynamic range (<100 μM) and 515 inactives, our model designed 5 new compounds predicted to have higher activity, together with predicted synthetic routes. All designs were were chemically synthesized and experimentally tested, and 3 have measurable activity against M^pro^. The top compound has comparable M^pro^ inhibition to the best in the training set, but with a different scaffold, and is active against the OC43 coronavirus in a live virus assay.

Algorithmic *de novo* design aims to automatically generate compounds that are chemically diverse, synthetically accessible and biologically active.^7^ Classic approaches apply heuristics to fragment and modify known active compounds, with the region of chemical space explored and synthetic accessibility constrained by those rules.^8,9,10^ Recent machine learning approaches explore chemical space in more abstract molecular representation space,^11,12^ but this often comes at the expense of synthetic accessibility.^13^ Our approach builds on rule-based fragmentation and molecule generation, but employs a method that combines regression and classification amid noisy data, and use of machine learning to predict synthesis routes. Our model comprises two parts: compound prioritisation and chemical space exploration.

Our compound prioritisation model aims to predict whether a designed compound is likely to be an improvement in activity over the incumbent. However, as is typical in the hit-expansion stage, bioactivity modelling is hindered by insufficient data where the majority of compounds are inactive, and noisy data as measurement variability increases for lower affinity compounds. Thresholding the data and framing the problem as classification of active/inactive would not allow us to rank compounds based on predicted improvement over the incumbent, yet the amount of measured bioactivity data and the measurement noise makes a regression approach challenging.

To overcome both challenges, we develop a learning-to-rank framework.^14,15^ Rather than training a regression model to predict the IC_50_ of a compound, we instead train a classifier to predict whether a compound is more or less active than another compound, with the input to the model being the *difference* in molecular descriptors between the molecules (see Fig. 1 for a schematic). This model accounts for both compounds with IC_50_ measurements and compounds that are simply inactive–active compounds are ranked by their IC_50_, all inactives with no measurable IC_50_ are considered less active than active compounds, and inactive–inactive pairs are ignored. Further, we account for noise by only considering IC_50_ differences amongst actives above 5 μM. We use the FastAI Tabular model,^16^ with input features generated from concatenated Morgan, Atom Pair, and Topological Torsion fingerprints implemented in RDkit,^17^ and dataset was randomly split into training (80%) and testing (20%); details about model implementation can be found in ESI† and source code.

**Fig. 1:**
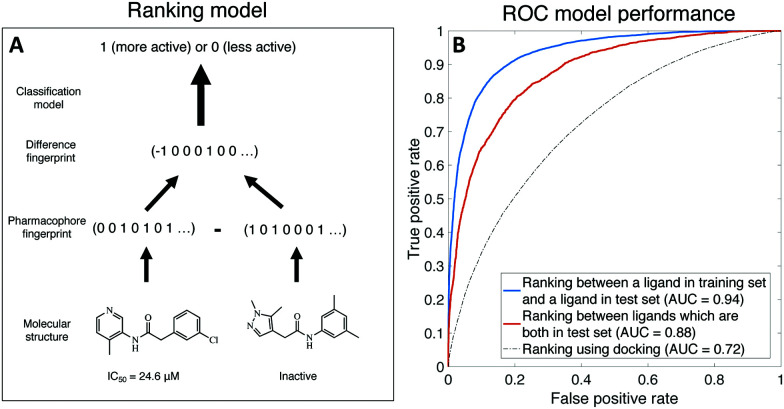
Relative ranking of ligands can be predicted by our learning-to-rank machine learning model. (A) A schematic of the model setup. A classifier takes the difference in pharmacophore fingerprint between two molecules and predicts where one molecule is more or less active than the other. (B) The receiver operating characteristic curve of classifying whether a molecule is more/less active than the other. AUC 95% CI reported in main text.

Fig. 1 shows that our binary ranking model achieves an AUC of 0.88 (95% CI: [0.83,0.96]) in ranking ligands within the test set, and AUC for 0.94 (95% CI: [0.91,0.98]) where we compare a ligand in the training set against another ligand in the test set; the latter is more relevant as our goal is finding ligands more active than the best incumbent. The 95% confidence interval is computed using bootstrapping. We also compare our model against OpenEyeâ€™s FRED hybrid docking mode as implemented in the “Classic OEDocking” floe, a physics-based docking algorithm, on the Orion online platform, which achieves AUC of 0.72; 95% CI: [0.722,0.723] (see ESI† for implementation details). Note that docking does not require ligand bioactivity as training data, thus is not a directly comparison to machine learning. In the ESI† Material, we discuss that our model ranks ligands better than a model that directly learns IC_50_ (AUC = 0.86; 95% CI: [0.71,0.95]).

Beyond train-test split, model performance can be evaluated from a time-split. Five months have elapsed from the time we deployed our model to select compounds to writing up the manuscript. During that time, the COVID Moonshot Consortium (a team of expert medicinal chemists) has independently designed, synthesised and tested 356 compounds,^18^ out of which 15% were better than the top **2** compounds (having IC_50_ comparable within error) in our dataset. Table 1 shows that our model has an enrichment factor of ∼2, *i.e.* if we rescore the 356 compounds synthesized by the medicinal chemistry team using our model, and pick the top 1%–10% percentile, the proportion of molecules that would be better than the top **2** compounds would be ∼2*x* higher than human selection.

**Table 1:** Enrichment factor for the time-split dataset, where we consider model performance on data arriving after the model has been deployed to generate compounds for synthesis and testing

Percentile	1%	2.5%	10%
Enrichment factor	1.7	2.3	1.7

Having demonstrated the accuracy of our ranking model, we now turn to chemical space exploration. We first consider a set of chemically reasonable perturbations (*e.g.* amide to retroamide, amide to urea), which is applied to the whole set of active molecules. We then fragment along synthetically accessible bonds (*e.g.* amides and aromatic C–C and C–N), and reconnect the synthons to generate an exhaustive library. The resulting library of 8.8 million generated molecules is scored using our ranking model by the probability of having a higher potency compared to the most potent molecule in the dataset.

Although virtual “reactions” were used to generate new molecules, the synthons are not necessarily off-the-shelf nor the reactions optimal. As such, we use a retrosynthesis predictor to triage based on synthetic accessibility. We fed top hits into Manifold, our platform for synthesis route prediction (https://postera.ai/manifold). Manifold searches for synthetic routes starting from purchasable molecules. The underlying technology is based on Molecular Transformer, a machine learning model for reaction prediction using sequence-to-sequence translation.^19,20^ The top 5 molecules with predicted routes <4 steps were synthesised and tested (Fig. 2A). For comparison, the most potent molecules from the training set are shown in Fig. 2B; **1–5** have Tanimoto similarity <0.48 (1024 bit ECFP6) to every molecule in the training set.

**Fig. 2:**
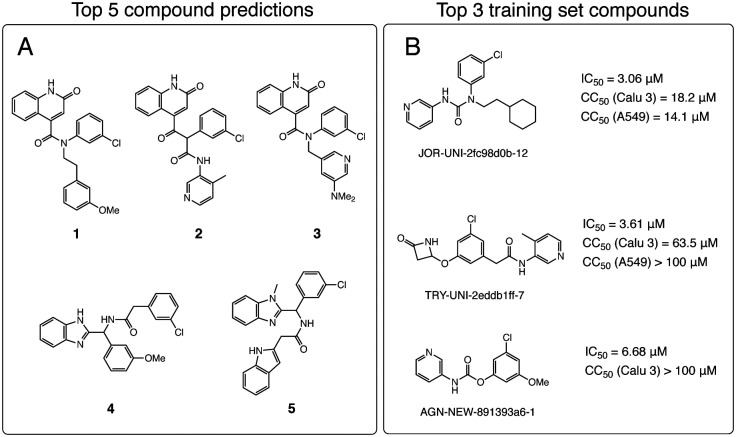
Our synthesis-driven design model prioritises molecular scaffold that are not in the top hits. (A) The 5 compounds selected by our methodology for synthesis and testing. (B) The top **3** compounds from the training set, with potency and cytotoxicity measurements.

Fig. 3 shows that for Compounds **1**, **2**, **4** and **5** our retrosynthesis algorithm generates successful routes, thus provides a reasonable estimate of synthetic complexity. The syntheses were carried out at the Wuxi AppTec and compounds were assayed as received. Minor variations in building blocks were employed depending on what was readily available. We note that our algorithm failed to estimate the synthetic complexity of Compound **3**. The final amide formation step was unexpectedly challenging, and no desired product was seen despite significant efforts in condition screening. Compound **3** was furnished *via* an alternative strategy, employing an Ullmann coupling to arylate the amide, which was not predicted by our approach.

**Fig. 3:**
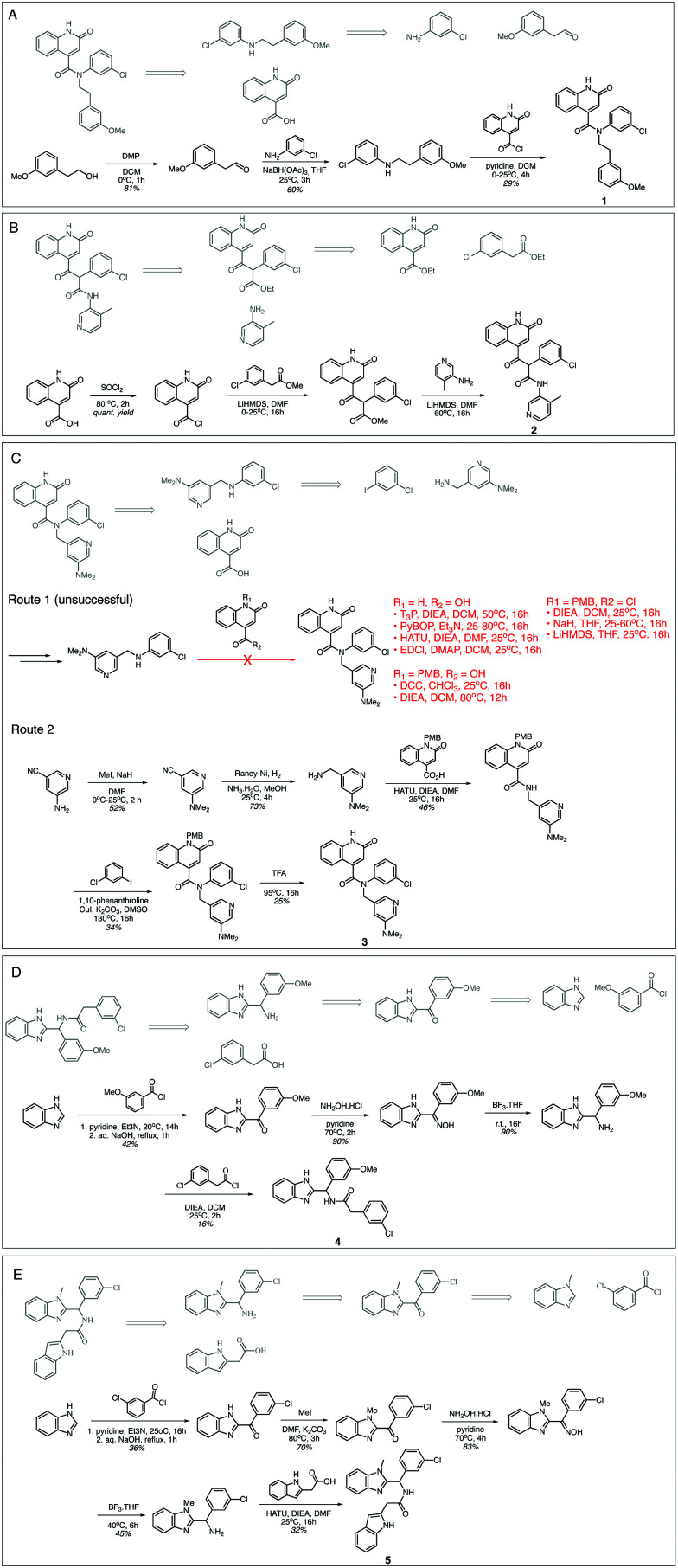
Model generated synthetic schemes that are experimentally validated. Schemes (A–E) show the synthesis schemes generated by our model (grey) and experimental schemes for Compounds **1–5**. The ESI† contains experimental procedures provided by our contract research organisation.

Compounds **1–5** were tested for Mpro activity using a fluorescence assay. Fig. 4 shows that Compounds **1–3** have IC_50_ within assay dynamic range (<100 μM), and Compound **1** has IC_50_ = 4.1 μM. Compound **1** is further assayed in live virus assays, with the less pathogenic OC43 coronavirus, showing EC_50_ = 13 μM and is not cytotoxic (CC_50_ > 100 μM against *A*_549_ cell line; CC_50_ is the concentration required to cause 50% cell death). We employ OC43 as a rapid surrogate assay for SARS-CoV-2 as the former can be done in a BSL-2 rather than BSL-3 lab. Interestingly, the top non-cytotoxic hit of the training set (TRY-UNI-2eddb1ff-7) does not show OC43 activity, showcasing the utility of using generative models to suggest new scaffolds with complementary physicochemical properties.

**Fig. 4:**
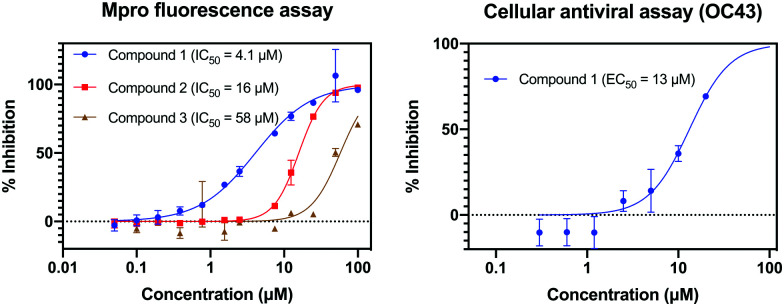
Three compounds generated using our synthesis-directed model exhibit Mpro activity. Our most active compound has measurable antiviral activity against the OC43 coronavirus and no measurable cytotoxic effect (CC_50_(*A*_549_) > 100 μM). 95% CI: IC50 (M^pro^)–Compound **1** [3.42,4.86] μM, Compound **2** [15.1,16.5] μM, Compound **3** [48.8,69.4] μM; EC50 (OC43)–Compound **1** [10.1, 18.4] μM. See ESI† for assay details.

In summary, we demonstrated the utility of a *de novo* design model, guided by estimation of synthetic complexity, for generating ideas in hit expansion. At the time of writing, the quinolone series is undergoing optimisation by the COVID Moonshot initiative (https://postera.ai/covid). Data for Compound **1–5** is registered as the *ALP-POS-ddb*41*b*15 series on the Moonshot platform.

J. D. C. acknowledges support from NIH grants P30 CA008748 and GM124270. W. M. acknowledges the support of the Gates Cambridge Trust. 

## Conflicts of interest

A. M. and A. A. L. are co-founders and shareholders of PostEra (https://postera.ai). J. D. C is a current member of the Scientific Advisory Board of OpenEye Scientific Software, Interline Therapeutics, and Redesign Science. The Chodera laboratory receives or has received funding from multiple sources,including the National Institutes of Health, the National Science Foundation, the Parker Institute for Cancer Immunotherapy, Relay Therapeutics, Entasis Therapeutics, Silicon Therapeutics, Interline Therapeutics, EMD Serono (Merck KGaA), AstraZeneca, Vir Biotechnology, Bayer, XtalPi, the Molecular Sciences Software Institute, the Starr Cancer Consortium, the Open Force Field Consortium, Cycle for Survival, a Louis V. Gerstner Young Investigator Award, and the Sloan Kettering Institute. A complete funding history for the Chodera lab can be found at http://choderalab.org/funding.

## Supplementary Material

CC-057-D1CC00050K-s001

CC-057-D1CC00050K-s002

CC-057-D1CC00050K-s003

CC-057-D1CC00050K-s004

CC-057-D1CC00050K-s005

CC-057-D1CC00050K-s006

CC-057-D1CC00050K-s007

CC-057-D1CC00050K-s008

CC-057-D1CC00050K-s009

CC-057-D1CC00050K-s010

CC-057-D1CC00050K-s011

CC-057-D1CC00050K-s012

CC-057-D1CC00050K-s013

CC-057-D1CC00050K-s014

CC-057-D1CC00050K-s015

CC-057-D1CC00050K-s016

CC-057-D1CC00050K-s017

CC-057-D1CC00050K-s018

CC-057-D1CC00050K-s019

CC-057-D1CC00050K-s020

CC-057-D1CC00050K-s021

CC-057-D1CC00050K-s022

CC-057-D1CC00050K-s023

CC-057-D1CC00050K-s024

CC-057-D1CC00050K-s025

CC-057-D1CC00050K-s026

CC-057-D1CC00050K-s027

CC-057-D1CC00050K-s028

CC-057-D1CC00050K-s029

CC-057-D1CC00050K-s030

CC-057-D1CC00050K-s031

CC-057-D1CC00050K-s032

CC-057-D1CC00050K-s033

CC-057-D1CC00050K-s034

CC-057-D1CC00050K-s035

CC-057-D1CC00050K-s036

CC-057-D1CC00050K-s037

CC-057-D1CC00050K-s038

CC-057-D1CC00050K-s039

CC-057-D1CC00050K-s040

CC-057-D1CC00050K-s041

CC-057-D1CC00050K-s042

CC-057-D1CC00050K-s043

CC-057-D1CC00050K-s044

CC-057-D1CC00050K-s045

CC-057-D1CC00050K-s046

CC-057-D1CC00050K-s047

CC-057-D1CC00050K-s048

CC-057-D1CC00050K-s049

CC-057-D1CC00050K-s050

CC-057-D1CC00050K-s051

CC-057-D1CC00050K-s052

CC-057-D1CC00050K-s053

CC-057-D1CC00050K-s054

CC-057-D1CC00050K-s055

CC-057-D1CC00050K-s056

CC-057-D1CC00050K-s057

CC-057-D1CC00050K-s058

CC-057-D1CC00050K-s059

CC-057-D1CC00050K-s060

CC-057-D1CC00050K-s061

CC-057-D1CC00050K-s062

CC-057-D1CC00050K-s063

CC-057-D1CC00050K-s064

CC-057-D1CC00050K-s065

CC-057-D1CC00050K-s066

CC-057-D1CC00050K-s067

CC-057-D1CC00050K-s068

CC-057-D1CC00050K-s069

CC-057-D1CC00050K-s070

CC-057-D1CC00050K-s071

CC-057-D1CC00050K-s072

CC-057-D1CC00050K-s073

CC-057-D1CC00050K-s074

CC-057-D1CC00050K-s075

CC-057-D1CC00050K-s076

CC-057-D1CC00050K-s077

CC-057-D1CC00050K-s078

CC-057-D1CC00050K-s079

CC-057-D1CC00050K-s080

CC-057-D1CC00050K-s081

CC-057-D1CC00050K-s082

CC-057-D1CC00050K-s083

CC-057-D1CC00050K-s084
