# Supplementary material for: Discovery of SARS-CoV-2 main protease inhibitors using a synthesis-directed de novo design model
Source: Chem Commun (Camb). 2021 May 6;57(48):5909–12. doi: 10.1039/d1cc00050k (PMC8204246; doi:10.1039/d1cc00050k)
Supplement: CC-057-D1CC00050K-s003 [file CC-057-D1CC00050K-s003.pdf]

Compound ID: 00000000

EB2224-19-P1A CDCl3 Bruker_NT-A_400MHZ

WuXi AppTec
An Integrated R&D Service Company

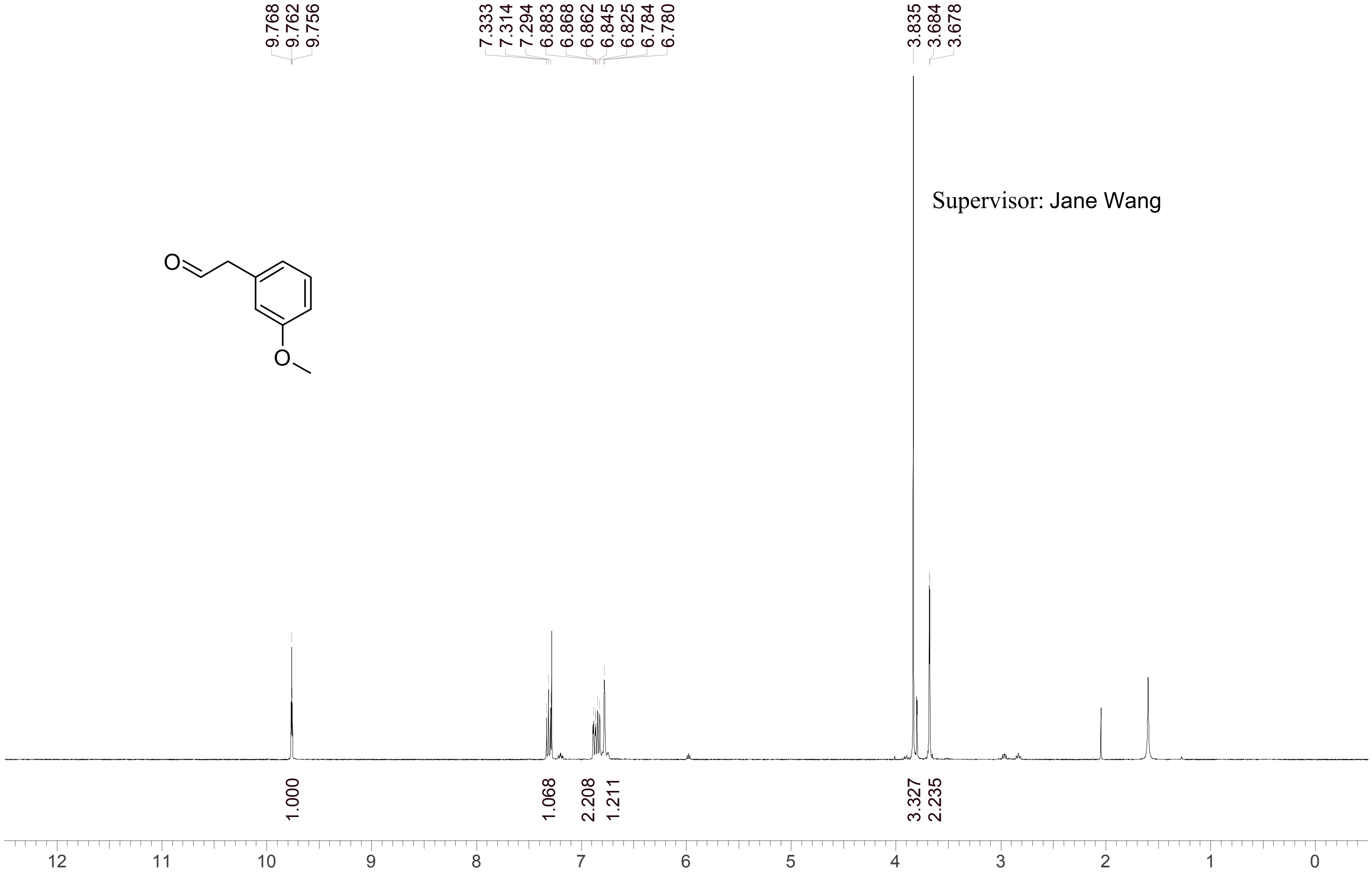

Acquisition Time (sec)	1.9999
Comment	EB2224-19-P1A CDCl3 Bruker_NT-A_400MHZ
Date	17 Jul 2020 03:57:45
Frequency (MHz)	400.1500
Nucleus	1H
Number of Transients	8
Origin	Avance
Original Points Count	16393
Owner	nmrsu
Points Count	65536
Pulse Sequence	zg30
Receiver Gain	101.00
SW(cyclical) (Hz)	8196.72
Solvent	CHLOROFORM-d
Spectrum Offset (Hz)	2471.2373
Spectrum Type	standard
Sweep Width (Hz)	8196.60
Temperature (degree C)	-273.000

^{1}H NMR (400MHz, CHLOROFORM-d) δ = 9.76 (t, *J*=2.4 Hz, 1H), 7.31 (t, *J*=7.9 Hz, 1H), 6.91 - 6.81 (m, 2H), 6.78 (d, *J*=1.5 Hz, 1H), 3.84 (s, 3H), 3.68 (d, *J*=2.4 Hz, 2H)

Confidential. For research only Not for regulatory filing

Operator:

Date:
