# Supplementary material for: Discovery of SARS-CoV-2 main protease inhibitors using a synthesis-directed de novo design model
Source: Chem Commun (Camb). 2021 May 6;57(48):5909–12. doi: 10.1039/d1cc00050k (PMC8204246; doi:10.1039/d1cc00050k)
Supplement: CC-057-D1CC00050K-s007 [file CC-057-D1CC00050K-s007.pdf]

Compound ID: ALP-POS-ddb41b15-4

EB2224-34-P1B MeOD Bruker_NT-C_400MHz

Supervisor: Jane Wang

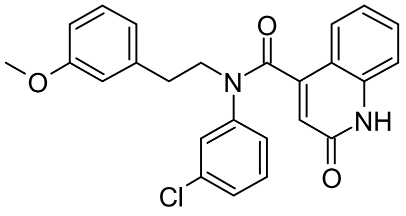

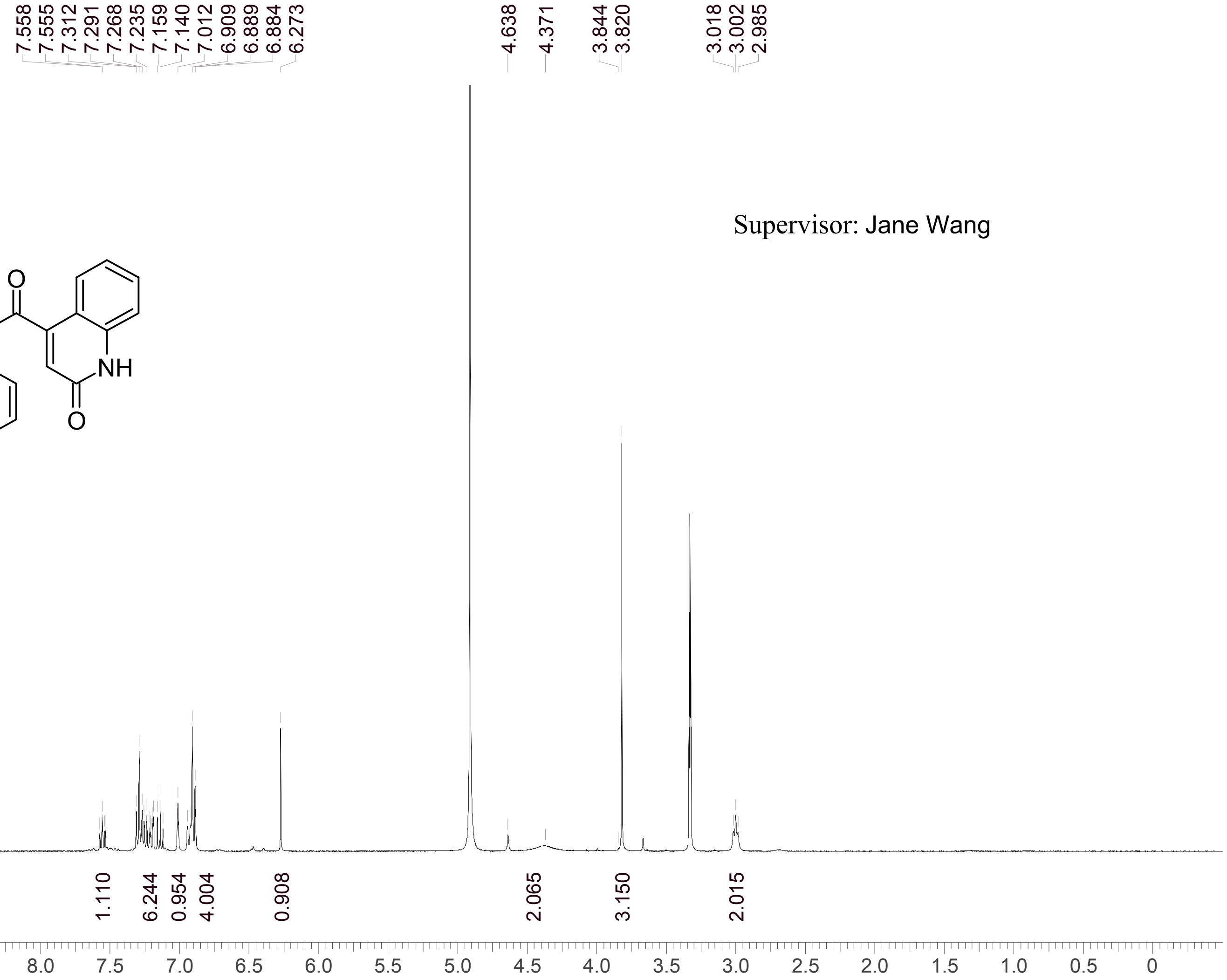

Acquisition Time (sec)	1.9999
Comment	EB2224-34-P1B MeOD Bruker_NT-C_400MHz
Date	26 Jul 2020 11:01:03
Frequency (MHz)	400.1400
Nucleus	1H
Number of Transients	8
Origin	Avance
Original Points Count	16393
Owner	nmrsu
Points Count	65536
Pulse Sequence	zg30
Receiver Gain	101.00
SW(cyclical) (Hz)	8196.72
Solvent	METHANOL-d4
Spectrum Offset (Hz)	2400.8411
Spectrum Type	standard
Sweep Width (Hz)	8196.60
Temperature (degree C)	21.663

^{1}H NMR (400MHz, METHANOL-d_4) δ = 7.56 (ddd, *J*=1.5, 7.0, 8.3 Hz, 1H), 7.32 - 7.11 (m, 6H), 7.01 (t, *J*=1.9 Hz, 1H), 6.95 - 6.88 (m, 4H), 6.27 (s, 1H), 4.66 - 4.26 (m, 2H), 3.82 (s, 3H), 3.00 (br t, *J*=6.7 Hz, 2H)

Confidential. For research only Not for regulatory filing

Operator:

Date:

Confidential. For research information only.

LCMS REPORT

Print time : 07/24/2020 17:01:48
Compound ID :ALP-POS-ddb41b15-4
Sample ID : EB2224-34-P1B
Injection Date :7/24/2020 4:59:58 PM
Injection Vol : 2ul
Location :tray1 vail10
Acq Method :5-95AB_1.5min_220&254_Shimadzu.lcm
Org DataFile :D:\DATA\2020\2007\200724\EB2224-34-P1B.lcd
Instrument & column:LCMS-SAW 1-2402
Chromolith Flash RP-18, 5um,3.0*25mm

Chromatogram

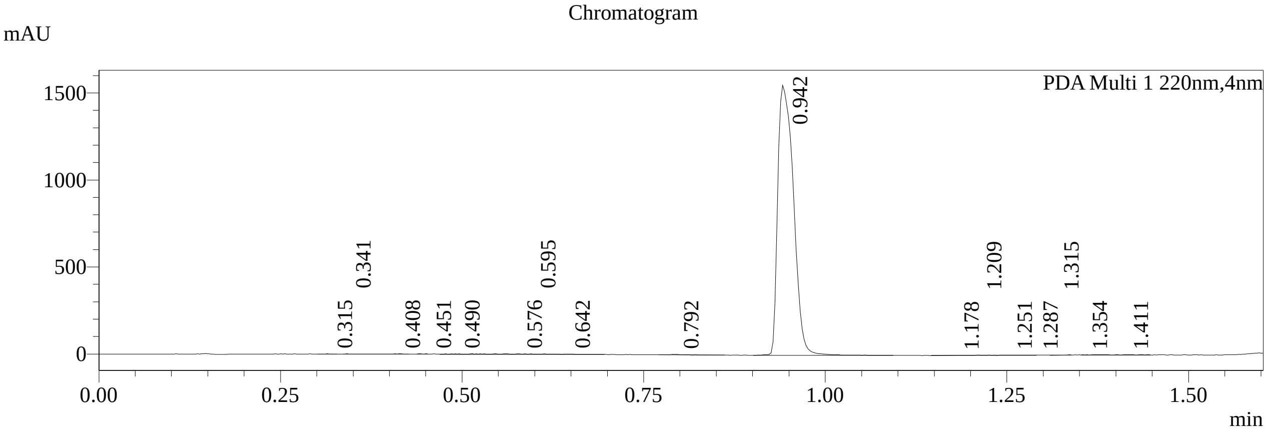

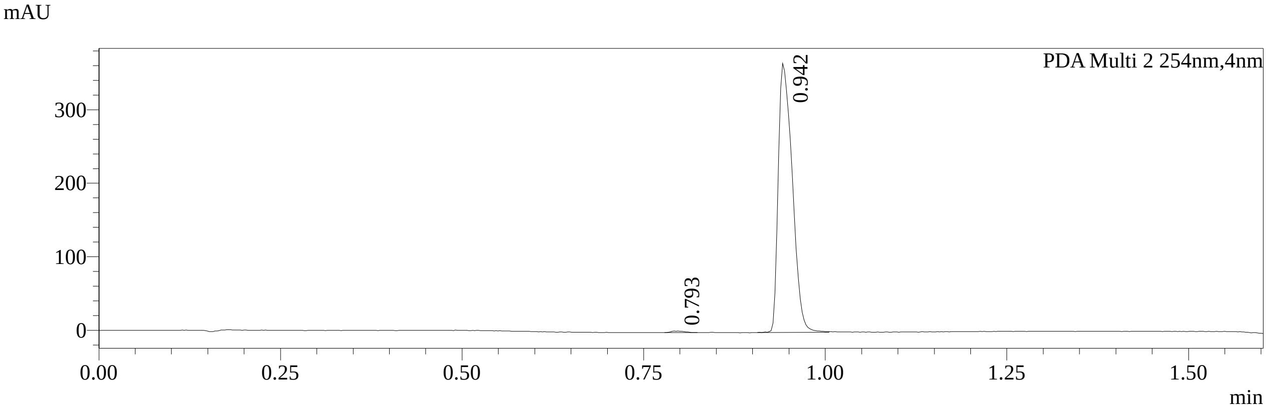

1 PDA Multi 1 / 220nm,4nm
2 PDA Multi 2 / 254nm,4nm

MS Chromatogram

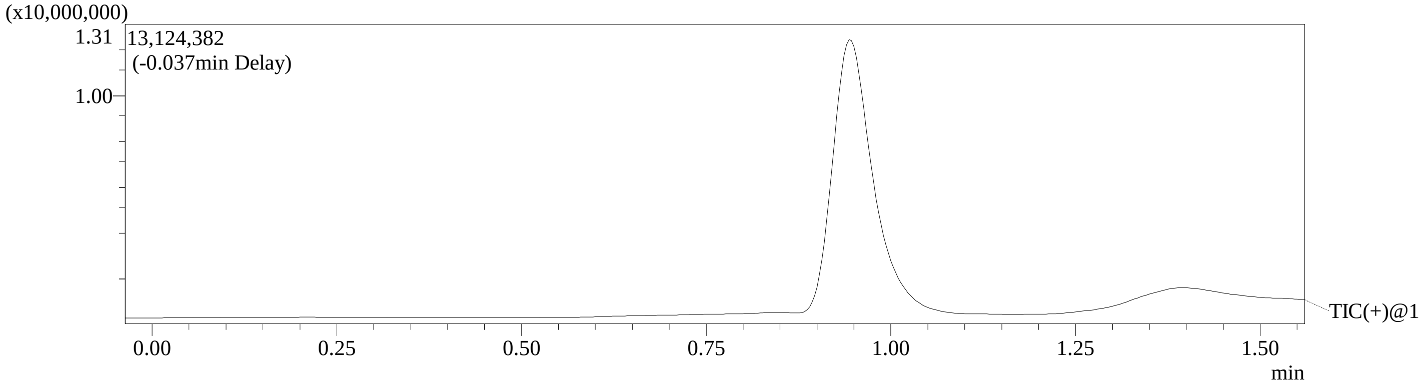

==

Integration Result

==

PDF created with pdfFactory Pro trial version www.pdffactory.com

PDA Ch1 220nm

Peak#	Ret. Time	Height	Height%	USP Width	Area	Area%
1	0.315	1179	0.074	0.031	1102	0.046
2	0.341	1526	0.096	0.009	1468	0.061
3	0.408	2072	0.131	0.064	3606	0.150
4	0.451	2010	0.127	0.018	1451	0.060
5	0.490	2662	0.168	0.056	3213	0.133
6	0.576	3174	0.200	0.170	12474	0.518
7	0.595	3163	0.200	0.410	8047	0.334
8	0.642	2136	0.135	0.078	3677	0.153
9	0.792	3370	0.213	0.040	4876	0.203
10	0.942	1551423	97.888	0.043	2347069	97.489
11	1.178	1301	0.082	0.073	1925	0.080
12	1.209	1553	0.098	0.104	3902	0.162
13	1.251	2081	0.131	0.050	3269	0.136
14	1.287	1673	0.106	0.074	1699	0.071
15	1.315	1866	0.118	0.040	2686	0.112
16	1.354	2170	0.137	0.053	3943	0.164
17	1.411	1543	0.097	0.052	3105	0.129

PDA Ch2 254nm

Peak#	Ret. Time	Height	Height%	USP Width	Area	Area%
1	0.793	2150	0.584	0.040	2886	0.579
2	0.942	365948	99.416	0.040	495606	99.421

Operator:_______________

Date:___________________

PDF created with pdfFactory Pro trial version www.pdffactory.com

Mass Spectrum

RetTime: 0.943 Datafile: D:\DATA\2020\2007\200724\EB2224-34-P1B.lcd

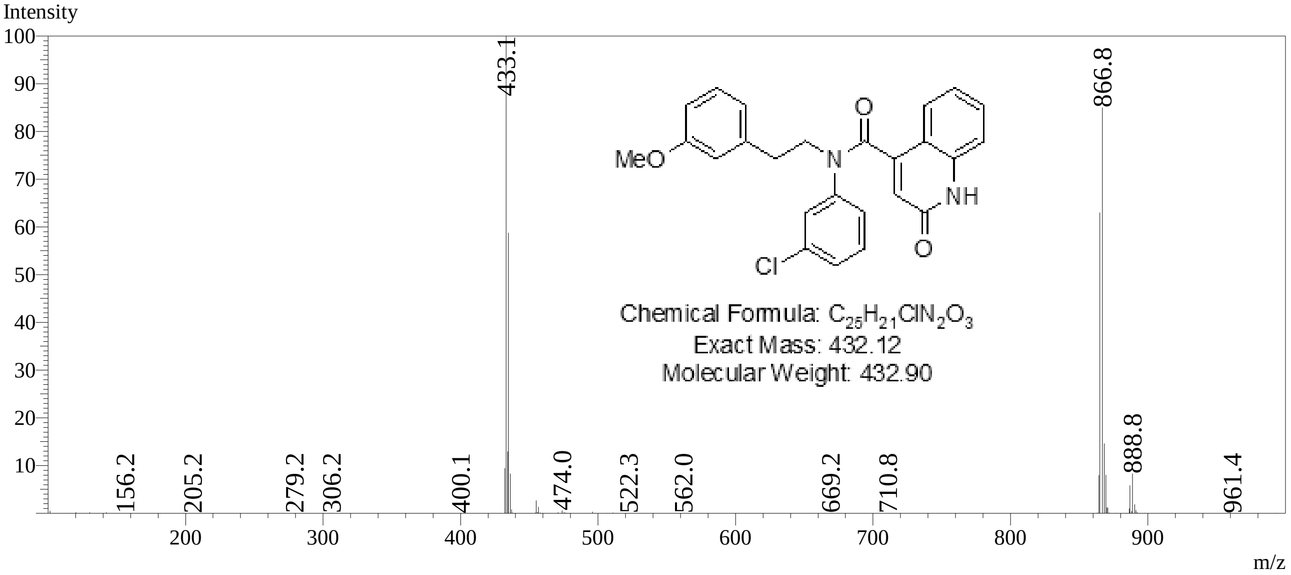

PDF created with pdfFactory Pro trial version www.pdffactory.com

:onfidential. For research information onl;

================== HPLC Analysis Report ==================

Sample ID	: EB2224-34-P1C
Compound ID	: ALP-POS-ddb41b15-4
Injecion Date	:7/24/2020 4:30:54 PM
Injection Volume	: 2 uL
Location	:Vial 81
Org Data File	: D:\DATA\2020\2007\200724\EB2224-34-P1C.lcd
Method File Name	: 10-80AB_8min_220&254.lcm
Instrument&Column	:HPLC-SAI 1-2402 Xtimate C18 2.1*30mm 3um

<Chromatogram>

Chromatogram
ALP-POS-ddb41b15-4 D:\DATA\2020\2007\200724\EB2224-34-P1C.lcd

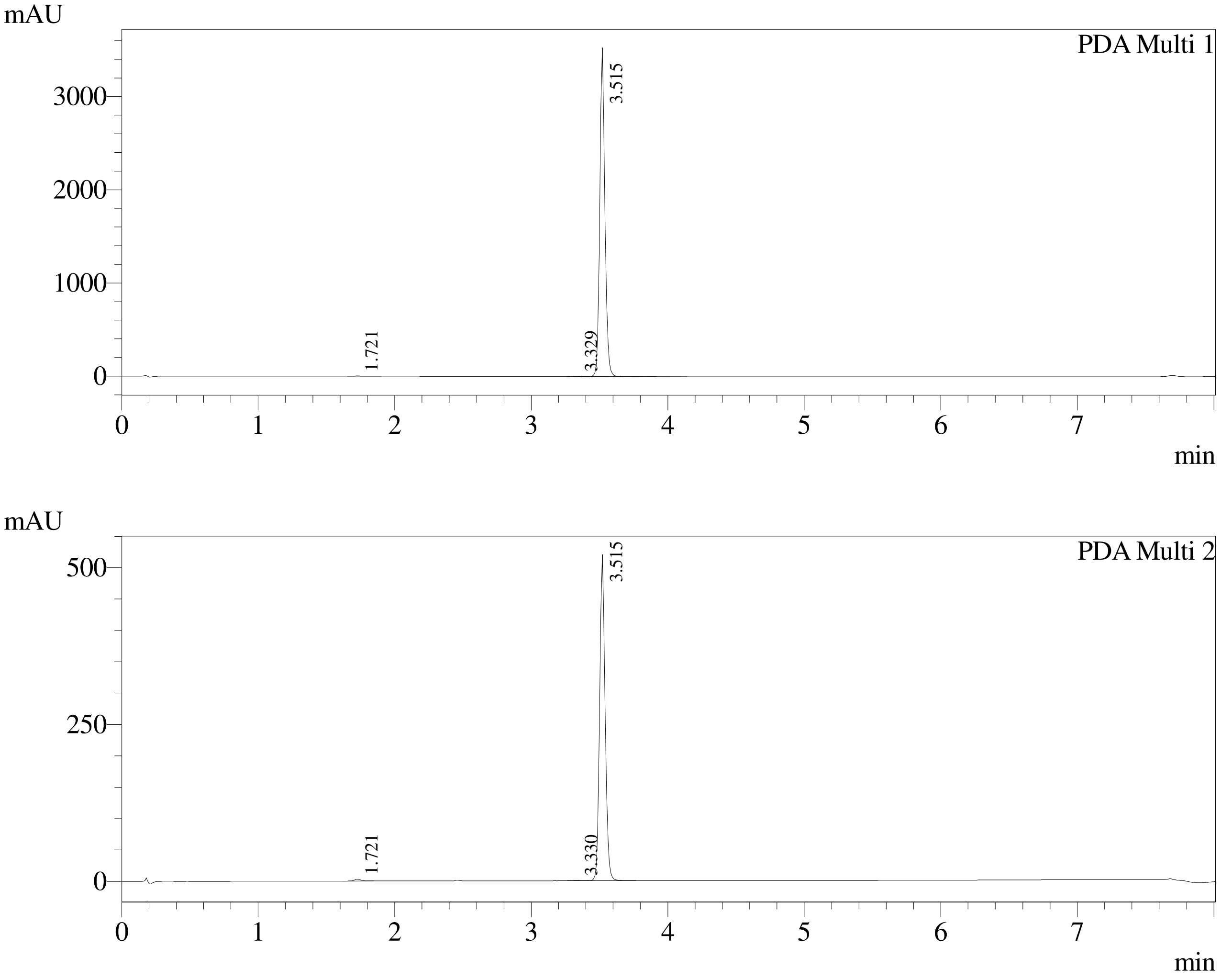

1 PDA Multi 1 / 220nm 4nm
2 PDA Multi 2 / 254nm 4nm

==
Integration Result
==

PeakTable

Ch1 220nm 4nm

Peak#	Ret. Time	Height	Height %	Area	Area %
1	1.721	3809	0.108	12525	0.136
2	3.329	4629	0.131	12715	0.138
3	3.515	3527699	99.761	9205901	99.727
Total		3536137	100.000	9231141	100.000

PeakTable

Ch2 254nm 4nm

Peak#	Ret. Time	Height	Height %	Area	Area %
1	1.721	2878	0.550	9559	0.651
2	3.330	830	0.159	2325	0.158
3	3.515	519715	99.292	1455706	99.190
Total		523422	100.000	1467589	100.000

PeakTable

Ch3

Operator:________________

Data:____________________
