# Supplementary material for: Discovery of SARS-CoV-2 main protease inhibitors using a synthesis-directed de novo design model
Source: Chem Commun (Camb). 2021 May 6;57(48):5909–12. doi: 10.1039/d1cc00050k (PMC8204246; doi:10.1039/d1cc00050k)
Supplement: CC-057-D1CC00050K-s009 [file CC-057-D1CC00050K-s009.pdf]

Compound ID: 00000000

EB2224-22-P1A CDCl3 Bruker_NT-C_400MHz

Supervisor: Jane Wang

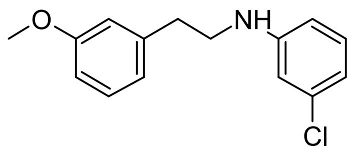

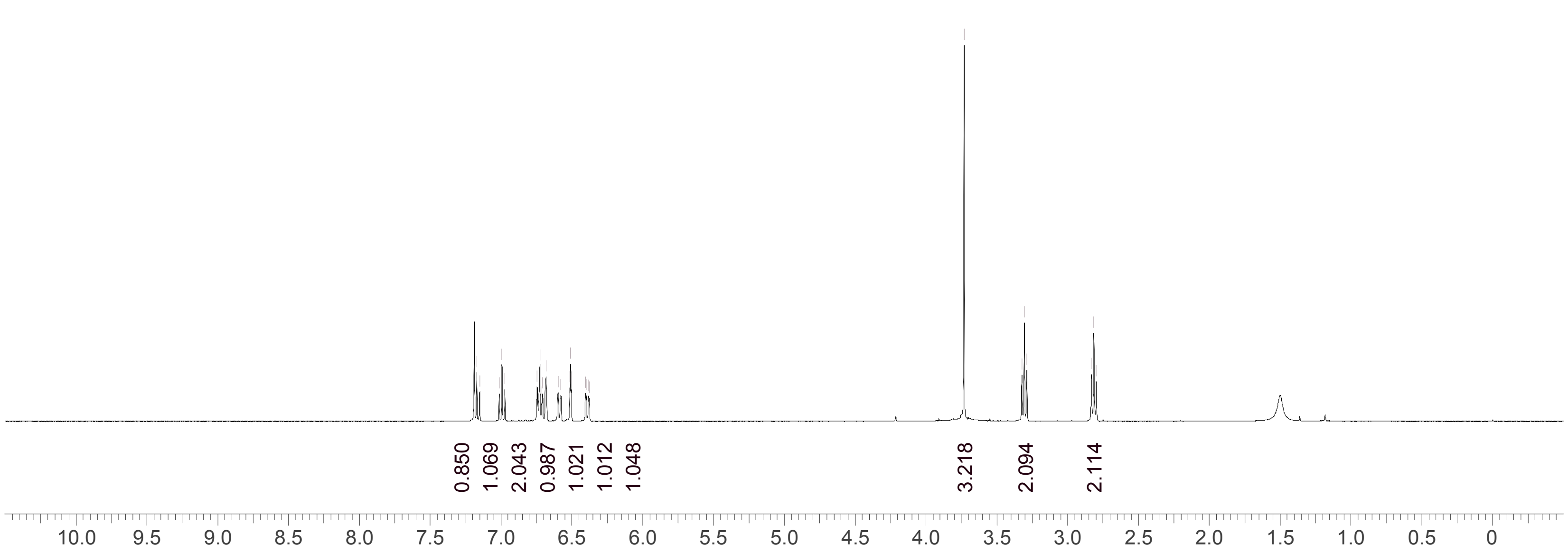

Acquisition Time (sec)	1.9999
Comment	EB2224-22-P1A CDCl3 Bruker_NT-C_400MHz
Date	19 Jul 2020 09:45:53
Frequency (MHz)	400.1400
Nucleus	1H
Number of Transients	8
Origin	Avance
Original Points Count	16393
Owner	nmrsu
Points Count	65536
Pulse Sequence	zg30
Receiver Gain	101.00
SW(cyclical) (Hz)	8196.72
Solvent	CHLOROFORM-d
Spectrum Offset (Hz)	2362.5042
Spectrum Type	standard
Sweep Width (Hz)	8196.60
Temperature (degree C)	23.170

^{1}H NMR (400MHz, CHLOROFORM-d) δ = 7.19 - 7.14 (m, 1H), 6.99 (t, *J*=8.1 Hz, 1H), 6.76 - 6.70 (m, 2H), 6.68 (s, 1H), 6.59 (d, *J*=7.8 Hz, 1H), 6.51 (t, *J*=2.1 Hz, 1H), 6.39 (dd, *J*=2.1, 8.1 Hz, 1H), 3.73 (s, 3H), 3.31 (t, *J*=6.9 Hz, 2H), 2.81 (t, *J*=6.9 Hz, 2H)

Confidential. For research only Not for regulatory filing

Operator:

Date:
