# Supplementary material for: Discovery of SARS-CoV-2 main protease inhibitors using a synthesis-directed de novo design model
Source: Chem Commun (Camb). 2021 May 6;57(48):5909–12. doi: 10.1039/d1cc00050k (PMC8204246; doi:10.1039/d1cc00050k)
Supplement: CC-057-D1CC00050K-s011 [file CC-057-D1CC00050K-s011.pdf]

Confidential. For research information only.

LCMS REPORT

Print time : 07/22/2020 13:05:39
Compound ID :1
Sample ID : EB2224-32-P1A
Injection Date :7/22/2020 1:03:50 PM
Injection Vol : 6ul
Location :tray1 vail76
Acq Method :5-95AB_1.5min_220&254_Shimadzu.lcm
Org DataFile :D:\DATA\2020\2007\200722\EB2224-32-P1A.lcd
Instrument & column:LCMS-SAW 1-2402
Chromolith Flash RP-18, 5um,3.0*25mm

Chromatogram

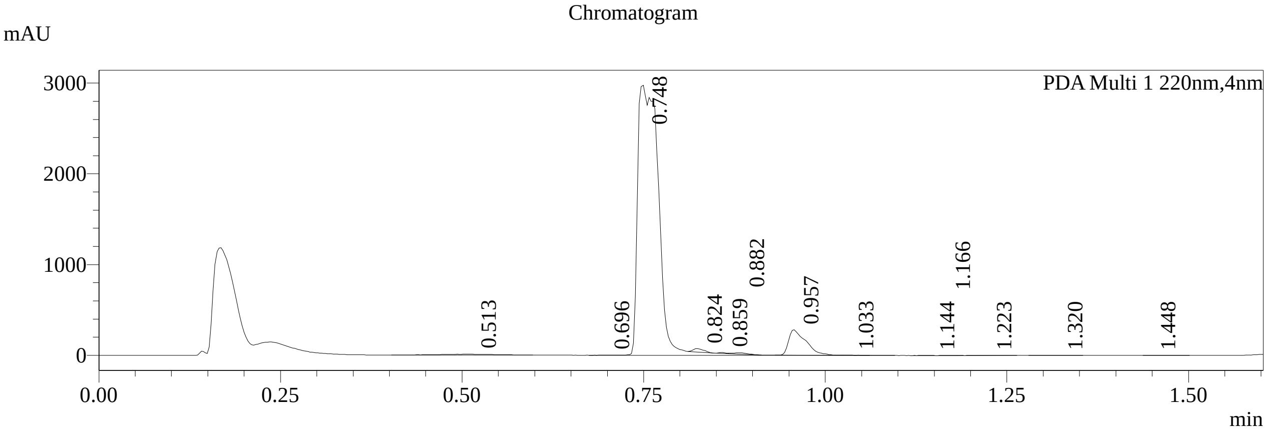

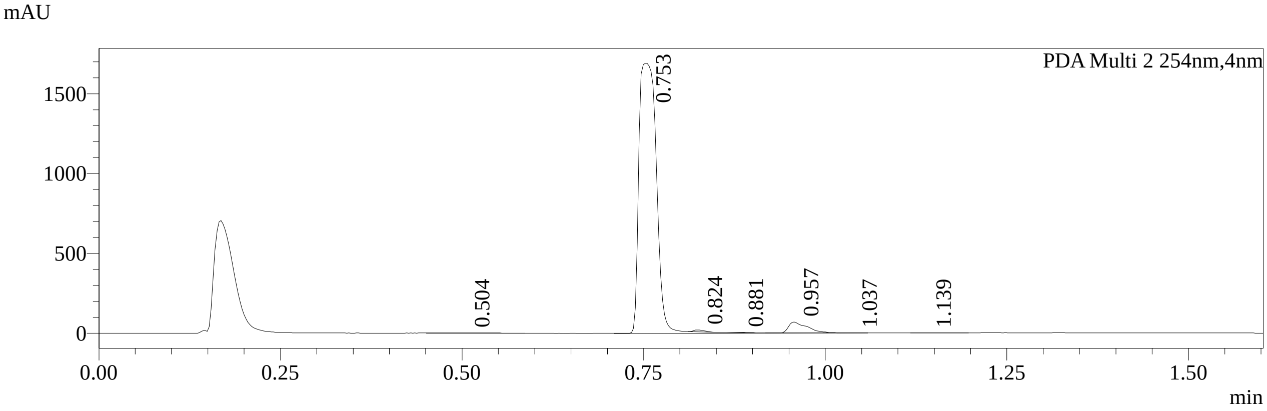

1 PDA Multi 1 / 220nm,4nm
2 PDA Multi 2 / 254nm,4nm

MS Chromatogram

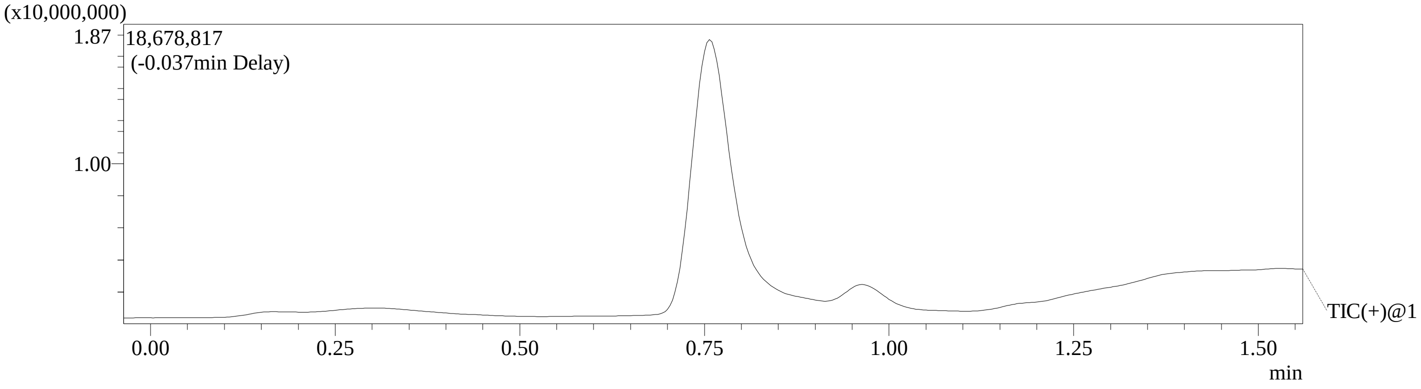

Integration Result

PDF created with pdfFactory Pro trial version www.pdffactory.com

PDA Ch1 220nm

Peak#	Ret. Time	Height	Height%	USP Width	Area	Area%
1	0.513	9503	0.284	0.131	48744	0.746
2	0.696	1848	0.055	0.051	2832	0.043
3	0.748	2975238	89.000	0.048	5934002	90.875
4	0.824	38252	1.144	0.033	38342	0.587
5	0.859	11114	0.332	0.032	8593	0.132
6	0.882	16861	0.504	0.050	22367	0.343
7	0.957	279953	8.374	0.047	458196	7.017
8	1.033	2500	0.075	0.043	3080	0.047
9	1.144	1220	0.036	0.036	1481	0.023
10	1.166	1505	0.045	0.031	1435	0.022
11	1.223	2091	0.063	0.066	5304	0.081
12	1.320	2012	0.060	0.047	4224	0.065
13	1.448	861	0.026	0.053	1254	0.019

PDA Ch2 254nm

Peak#	Ret. Time	Height	Height%	USP Width	Area	Area%
1	0.504	2695	0.152	0.154	13245	0.444
2	0.753	1688570	95.162	0.042	2831848	95.009
3	0.824	11766	0.663	0.034	12389	0.416
4	0.881	1831	0.103	0.039	1981	0.066
5	0.957	68135	3.840	0.054	118834	3.987
6	1.037	777	0.044	0.055	1260	0.042
7	1.139	638	0.036	0.027	1067	0.036

Operator:_______________

Date:___________________

PDF created with pdfFactory Pro trial version www.pdffactory.com

Mass Spectrum

RetTime: 0.747 Datafile: D:\DATA\2020\2007\200722\EB2224-32-P1A.lcd

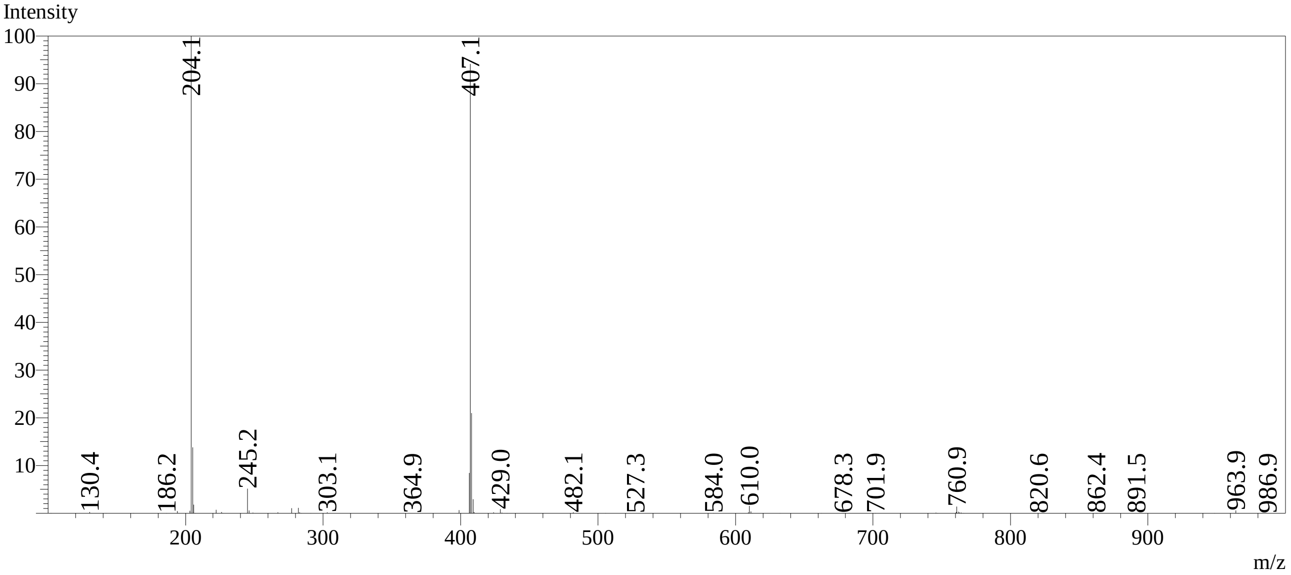

RetTime: 0.957 Datafile: D:\DATA\2020\2007\200722\EB2224-32-P1A.lcd

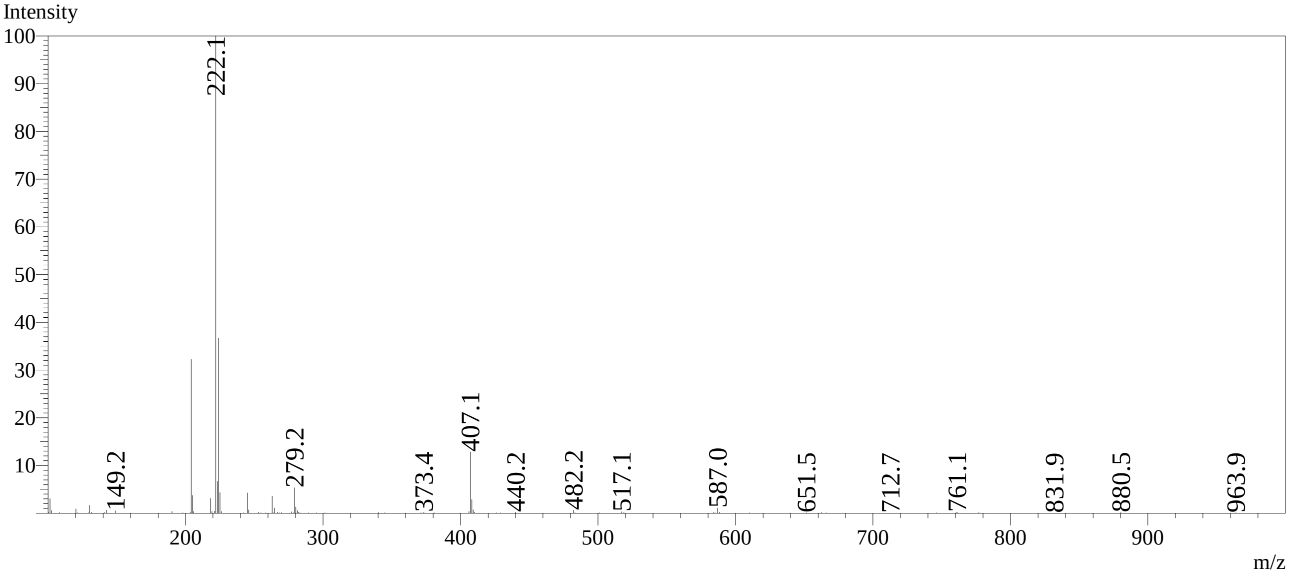

PDF created with pdfFactory Pro trial version www.pdffactory.com
