# Supplementary material for: Discovery of SARS-CoV-2 main protease inhibitors using a synthesis-directed de novo design model
Source: Chem Commun (Camb). 2021 May 6;57(48):5909–12. doi: 10.1039/d1cc00050k (PMC8204246; doi:10.1039/d1cc00050k)
Supplement: CC-057-D1CC00050K-s019 [file CC-057-D1CC00050K-s019.pdf]

Confidential. For research information only.

LCMS REPORT

Print time : 08/24/2020 16:23:39
Compound ID :1
Sample ID : EB2224-87-P1B
Injection Date :8/24/2020 4:21:55 PM
Injection Vol : 6ul
Location :tray1 vail23
Acq Method :5-95AB_1.5min_220&254_Shimadzu.lcm
Org DataFile :D:\DATA\2020\2008\200824\EB2224-87-P1B.lcd
Instrument & column:LCMS-SAW 1-2402
Chromolith Flash RP-18, 5um,3.0*25mm

Chromatogram

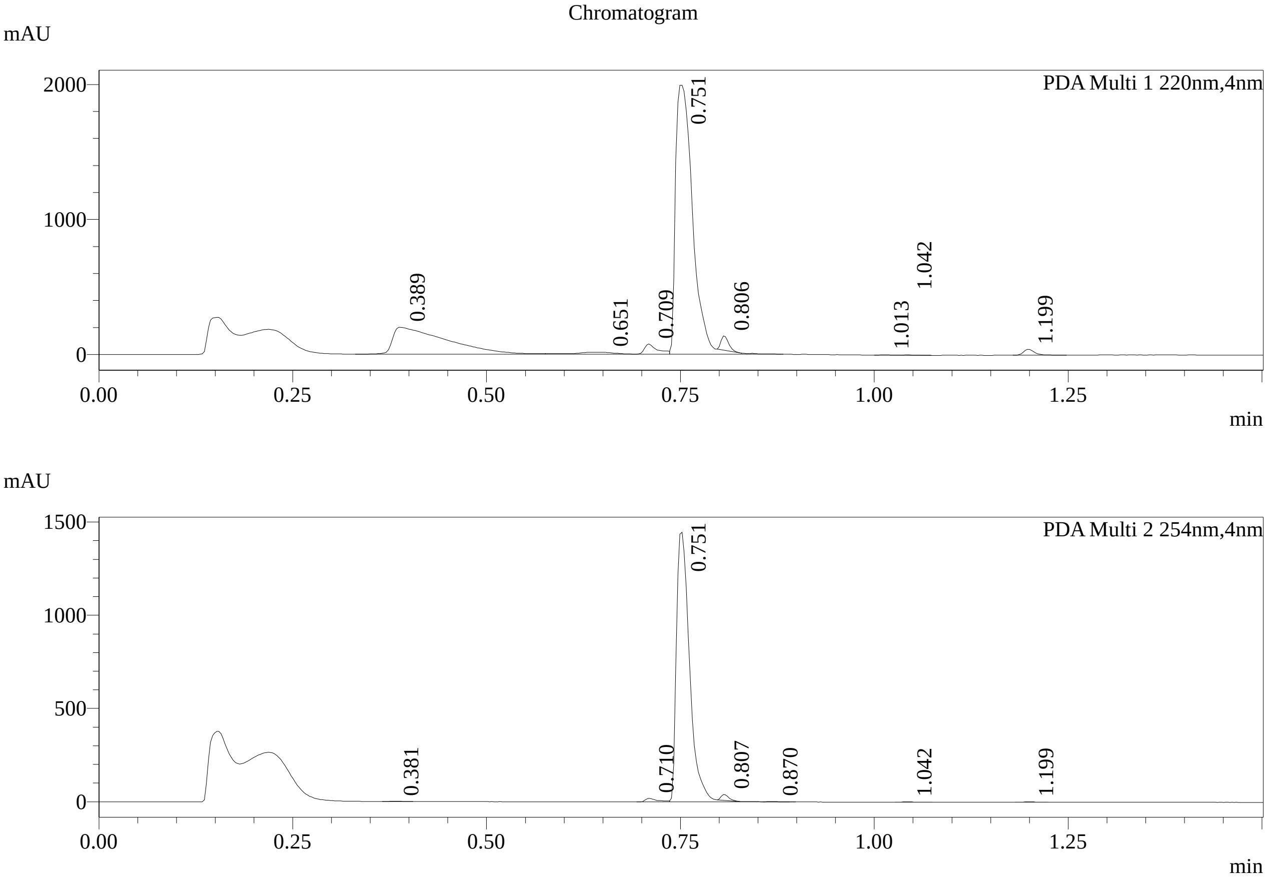

1 PDA Multi 1 / 220nm,4nm
2 PDA Multi 2 / 254nm,4nm

MS Chromatogram

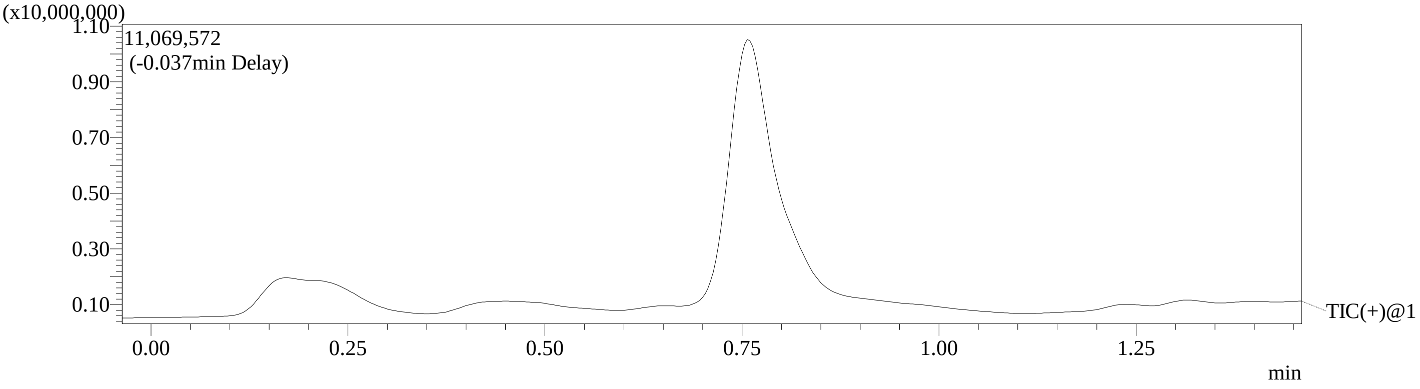

==

Integration Result

==

PDF created with pdfFactory Pro trial version www.pdffactory.com

PDA Ch1 220nm

Peak#	Ret. Time	Height	Height%	USP Width	Area	Area%
1	0.389	199314	8.181	0.139	958974	22.348
2	0.651	14849	0.610	0.078	51504	1.200
3	0.709	74996	3.078	0.030	89322	2.082
4	0.751	1991808	81.758	0.040	3071969	71.588
5	0.806	105484	4.330	0.021	69805	1.627
6	1.013	3551	0.146	0.023	2817	0.066
7	1.042	2364	0.097	0.027	2469	0.058
8	1.199	43864	1.800	0.028	44302	1.032

PDA Ch2 254nm

Peak#	Ret. Time	Height	Height%	USP Width	Area	Area%
1	0.381	2077	0.138	0.025	1856	0.105
2	0.710	19731	1.311	0.030	21946	1.241
3	0.751	1445925	96.109	0.035	1718911	97.182
4	0.807	30584	2.033	0.021	20297	1.148
5	0.870	1396	0.093	0.027	1172	0.066
6	1.042	2755	0.183	0.028	2600	0.147
7	1.199	1989	0.132	0.030	1964	0.111

Operator:_______________

Date:___________________

PDF created with pdfFactory Pro trial version www.pdffactory.com

Mass Spectrum

RetTime: 0.390 Datafile: D:\DATA\2020\2008\200824\EB2224-87-P1B.lcd

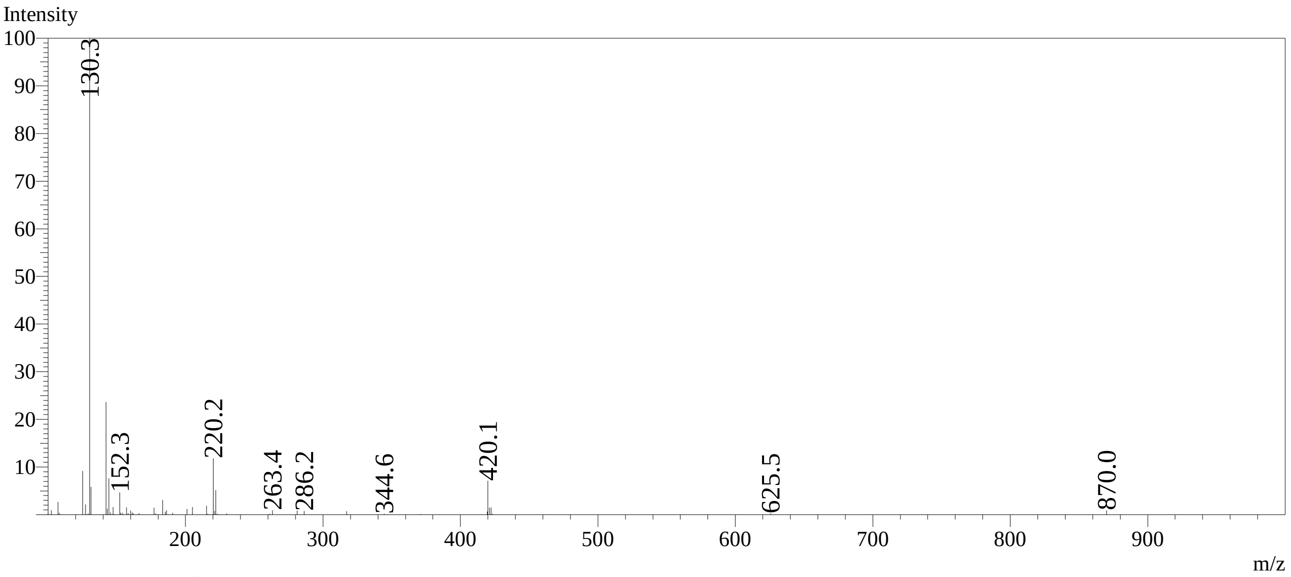

RetTime: 0.710 Datafile: D:\DATA\2020\2008\200824\EB2224-87-P1B.lcd

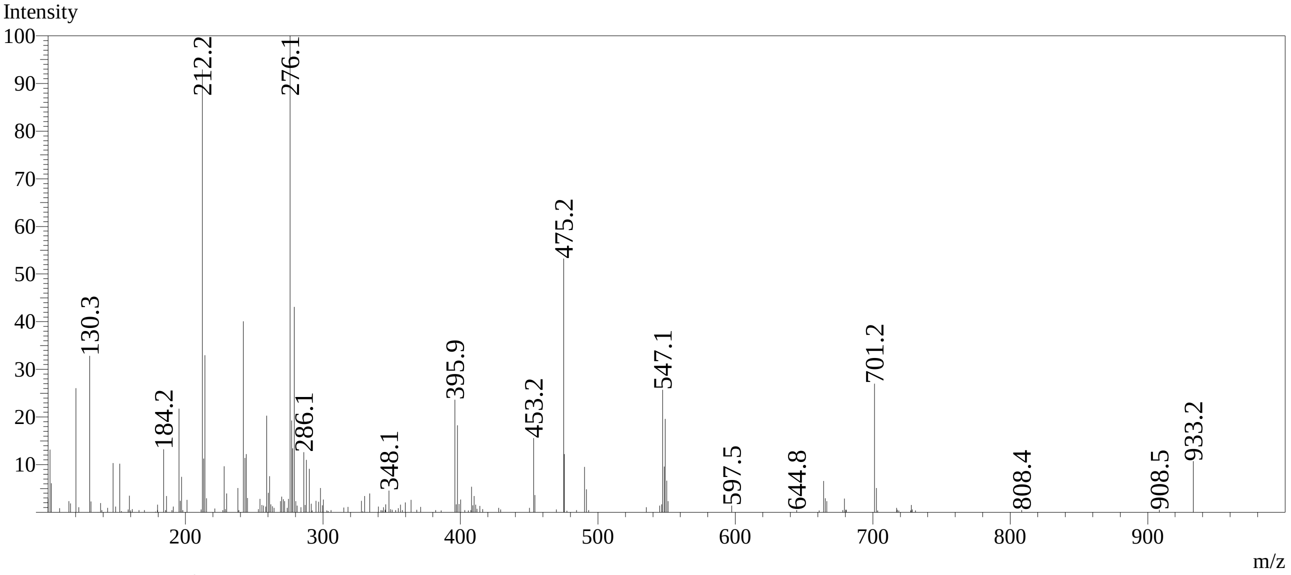

RetTime: 0.750 Datafile: D:\DATA\2020\2008\200824\EB2224-87-P1B.lcd

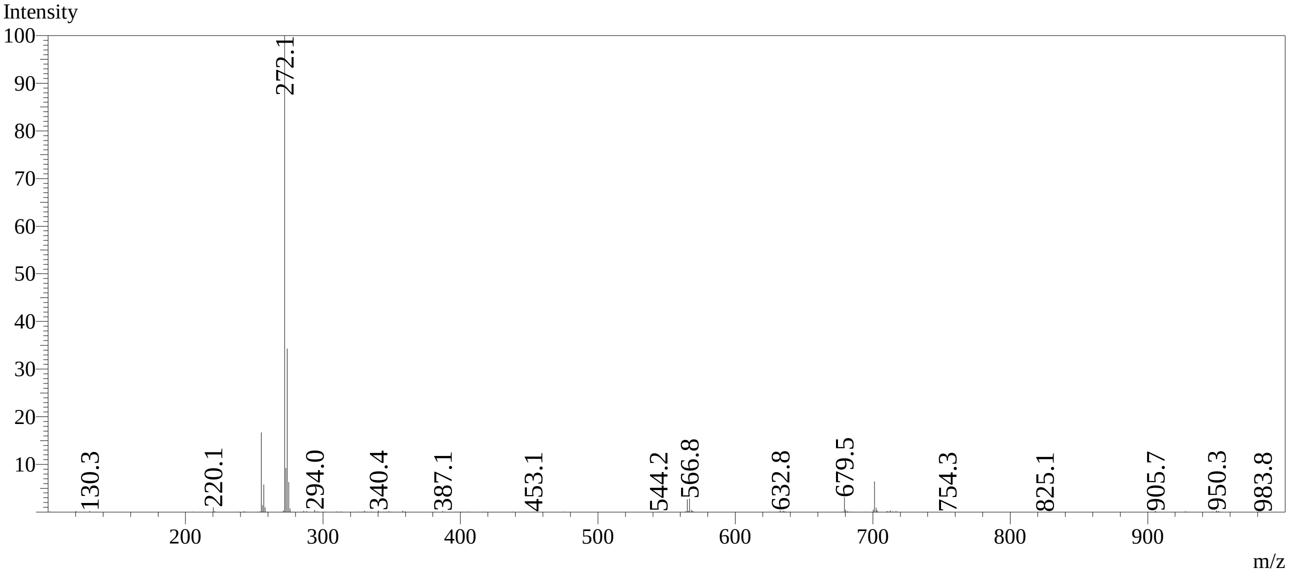

PDF created with pdfFactory Pro trial version www.pdffactory.com
