# Supplementary material for: Discovery of SARS-CoV-2 main protease inhibitors using a synthesis-directed de novo design model
Source: Chem Commun (Camb). 2021 May 6;57(48):5909–12. doi: 10.1039/d1cc00050k (PMC8204246; doi:10.1039/d1cc00050k)
Supplement: CC-057-D1CC00050K-s023 [file CC-057-D1CC00050K-s023.pdf]

Compound ID: ALP-POS-ddb41b15-8

EB2224-107-P1A DMSO Bruker_NT-C_400MHz

WuXi AppTec
An Integrated R&D Service Company

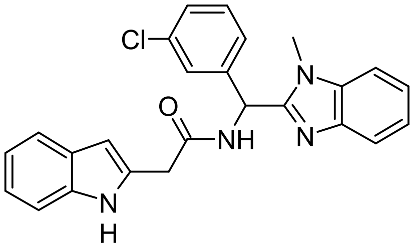

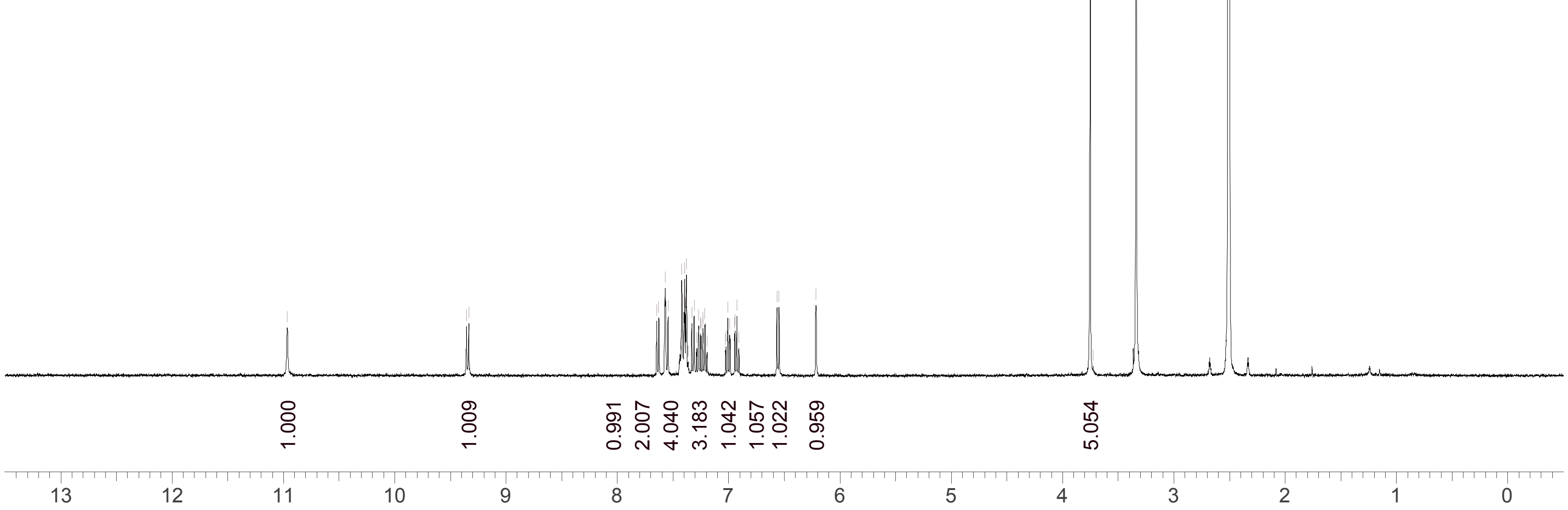

Acquisition Time (sec)	1.9999
Comment	EB2224-107-P1A DMSO Bruker_NT-C_400MHz
Date	31 Aug 2020 12:55:02
Frequency (MHz)	400.1400
Nucleus	1H
Number of Transients	8
Origin	Avance
Original Points Count	16393
Owner	nmrsu
Points Count	65536
Pulse Sequence	zg30
Receiver Gain	101.00
SW(cyclical) (Hz)	8196.72
Solvent	DMSO-d6
Spectrum Offset (Hz)	2400.8411
Spectrum Type	standard
Sweep Width (Hz)	8196.60
Temperature (degree C)	21.827

^{1}H NMR (400MHz, DMSO-d_6) δ = 10.97 (s, 1H), 9.35 (d, *J*=8.0 Hz, 1H), 7.64 (d, *J*=7.5 Hz, 1H), 7.59 - 7.52 (m, 2H), 7.44 - 7.36 (m, 4H), 7.34 - 7.19 (m, 3H), 7.04 - 6.97 (m, 1H), 6.96 - 6.89 (m, 1H), 6.56 (d, *J*=8.1 Hz, 1H), 6.21 (s, 1H), 3.75 (s, 5H)

Confidential. For research only Not for regulatory filing

Operator:

Date:

Confidential. For research information only.

LCMS REPORT

Print time : 08/31/2020 10:56:33
Compound ID :ALP-POS-ddb41b15-8
Sample ID : EB2224-107-P1C
Injection Date :8/31/2020 10:54:31 AM
Injection Vol : 6ul
Location :tray1 vail48
Acq Method :5-95AB_1.5min_220&254_Shimadzu.lcm
Org DataFile :D:\DATA\2020\2008\200831\EB2224-107-P1C.lcd
Instrument & column:LCMS-SAW 1-2402
Chromolith Flash RP-18, 5um,3.0*25mm

Chromatogram

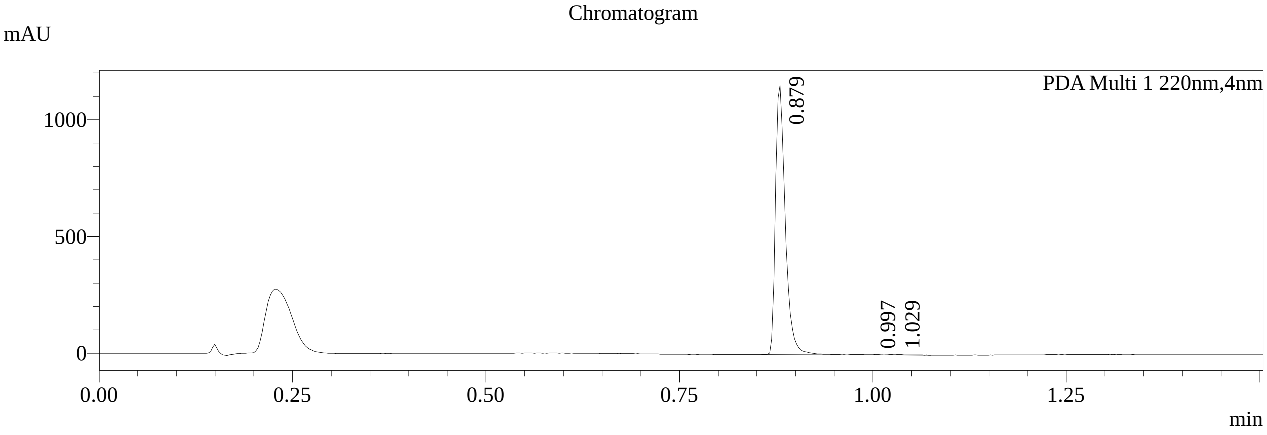

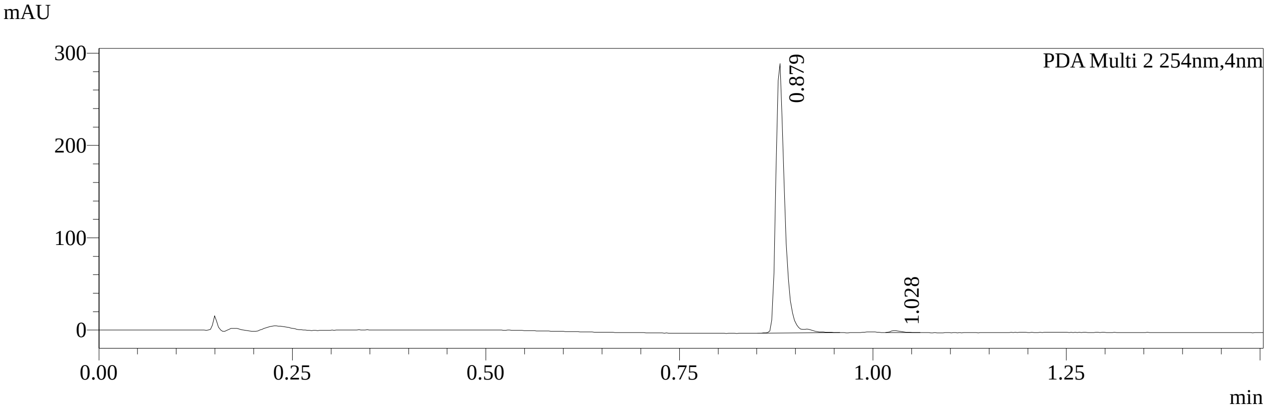

1 PDA Multi 1 / 220nm,4nm
2 PDA Multi 2 / 254nm,4nm

MS Chromatogram

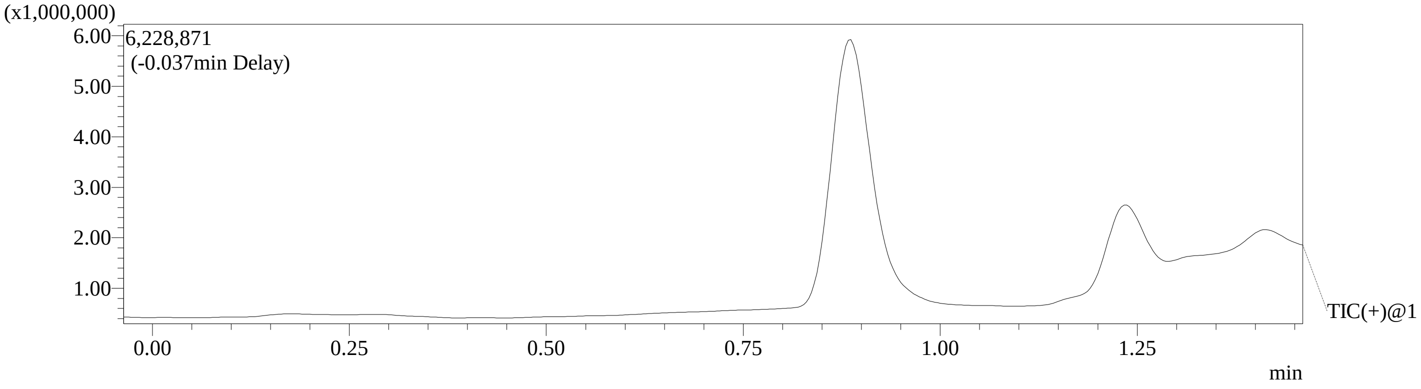

Integration Result

PDF created with pdfFactory Pro trial version www.pdffactory.com

PDA Ch1 220nm

Peak#	Ret. Time	Height	Height%	USP Width	Area	Area%
1	0.879	1152297	99.584	0.026	1022568	99.434
2	0.997	2021	0.175	0.044	2832	0.275
3	1.029	2796	0.242	0.029	2990	0.291

PDA Ch2 254nm

Peak#	Ret. Time	Height	Height%	USP Width	Area	Area%
1	0.879	292115	99.233	0.023	238162	99.004
2	1.028	2258	0.767	0.032	2397	0.996

Operator:_______________

Date:___________________

PDF created with pdfFactory Pro trial version www.pdffactory.com

Mass Spectrum

RetTime: 0.880 Datafile: D:\DATA\2020\2008\200831\EB2224-107-P1C.lcd

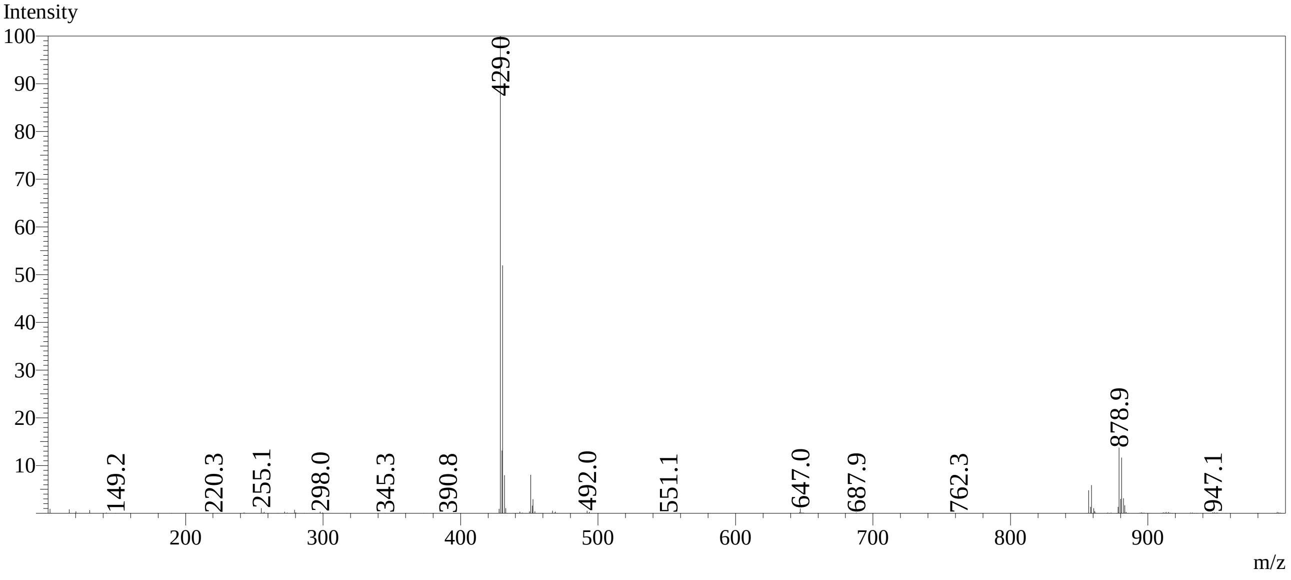

PDF created with pdfFactory Pro trial version www.pdffactory.com

:onfidential. For research information only

================== HPLC Analysis Report ==================

Sample ID	: EB2224-107-P1C2
Compound ID	: ALP-POS-ddb41b15-8
Injecion Date	:8/31/2020 11:42:31 AM
Injection Volume	: 6 uL
Location	:Vial 105
Org Data File	: D:\DATA\2020\2008\200831\EB2224-107-P1C2.lcd
Method File Name	: 10-80AB_8min_220&254.lcm
Instrument&Column	:HPLC-SAI 1-2402 Xtimate C18 2.1*30mm 3um

<Chromatogram>

Chromatogram
ALP-POS-ddb41b15-8 D:\DATA\2020\2008\200831\EB2224-107-P1C2.lcd

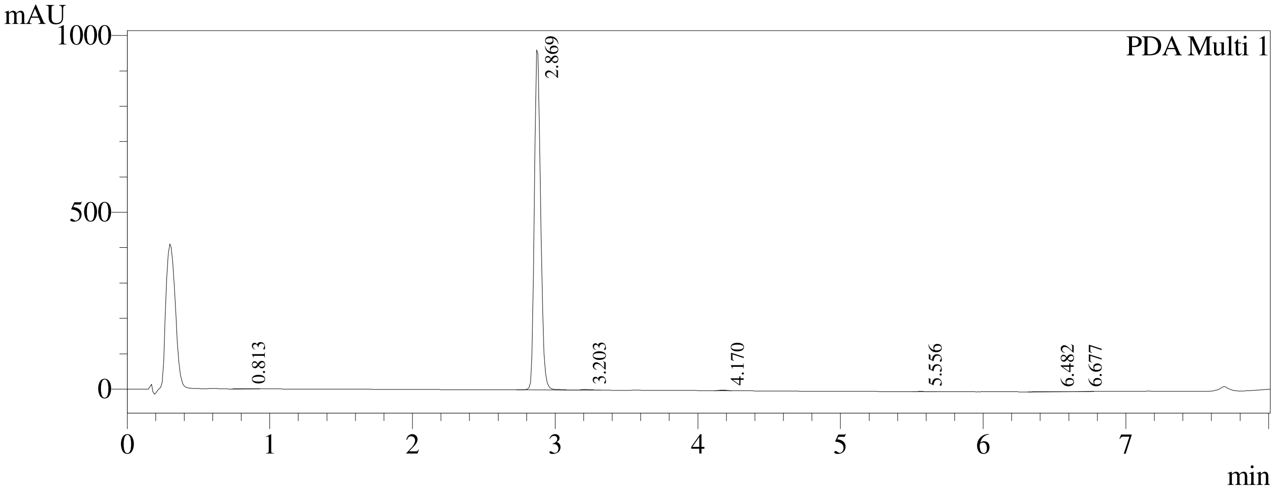

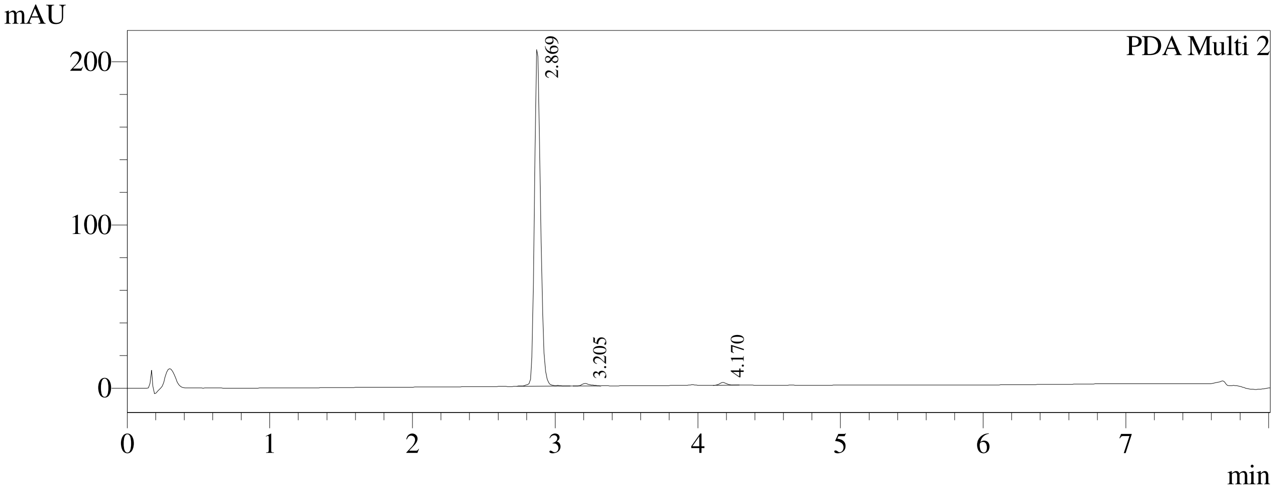

1 PDA Multi 1 / 220nm 4nm
2 PDA Multi 2 / 254nm 4nm

Integration Result

PeakTable

Ch1 220nm 4nm

Peak#	Ret. Time	Height	Height %	Area	Area %
1	0.813	797	0.082	4564	0.152
2	2.869	961435	99.318	2963437	98.977
3	3.203	1801	0.186	6987	0.233
4	4.170	2219	0.229	7021	0.235
5	5.556	680	0.070	2356	0.079
6	6.482	790	0.082	8033	0.268
7	6.677	312	0.032	1654	0.055
Total		968032	100.000	2994052	100.000

PeakTable

Ch2 254nm 4nm

Peak#	Ret. Time	Height	Height %	Area	Area %
1	2.869	206124	98.387	616972	98.027
2	3.205	1582	0.755	6216	0.988
3	4.170	1797	0.858	6204	0.986
Total		209504	100.000	629392	100.000

PeakTable

Ch3

Operator:_______________

Data:___________________
