# Supplementary material for: Discovery of SARS-CoV-2 main protease inhibitors using a synthesis-directed de novo design model
Source: Chem Commun (Camb). 2021 May 6;57(48):5909–12. doi: 10.1039/d1cc00050k (PMC8204246; doi:10.1039/d1cc00050k)
Supplement: CC-057-D1CC00050K-s027 [file CC-057-D1CC00050K-s027.pdf]

Compound ID: 00000000

EB2224-75-P1A DMSO Bruker_NT-C_400MHz

WuXi AppTec
An Integrated R&D Service Company

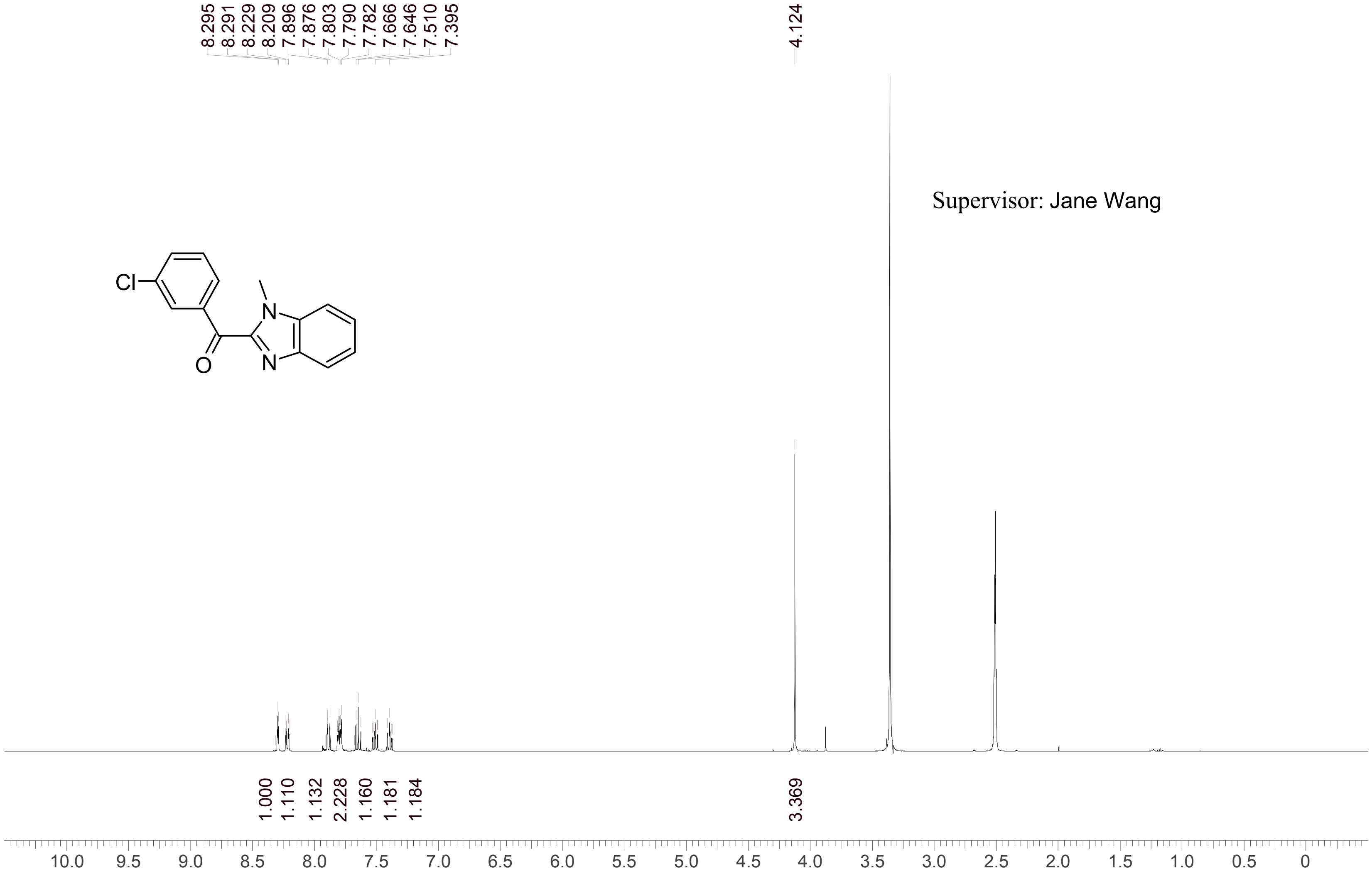

Supervisor: Jane Wang

Acquisition Time (sec)	1.9999
Comment	EB2224-75-P1A DMSO Bruker_NT-C_400MHz
Date	17 Aug 2020 09:45:06
Frequency (MHz)	400.1400
Nucleus	1H
Number of Transients	8
Origin	Avance
Original Points Count	16393
Owner	nmrsu
Points Count	65536
Pulse Sequence	zg30
Receiver Gain	101.00
SW(cyclical) (Hz)	8196.72
Solvent	DMSO-d6
Spectrum Offset (Hz)	2400.8411
Spectrum Type	standard
Sweep Width (Hz)	8196.60
Temperature (degree C)	22.713

^{1}H NMR (400MHz, DMSO-d_6) δ = 8.30 (t, *J*=1.8 Hz, 1H), 8.22 (td, *J*=1.3, 7.8 Hz, 1H), 7.91 - 7.87 (m, 1H), 7.83 - 7.77 (m, 2H), 7.68 - 7.62 (m, 1H), 7.51 (ddd, *J*=1.1, 7.1, 8.3 Hz, 1H), 7.39 (ddd, *J*=1.1, 7.1, 8.2 Hz, 1H), 4.12 (s, 3H)

Confidential. For research only Not for regulatory filing

Operator:

Date:
