# Supplementary material for: Discovery of SARS-CoV-2 main protease inhibitors using a synthesis-directed de novo design model
Source: Chem Commun (Camb). 2021 May 6;57(48):5909–12. doi: 10.1039/d1cc00050k (PMC8204246; doi:10.1039/d1cc00050k)
Supplement: CC-057-D1CC00050K-s029 [file CC-057-D1CC00050K-s029.pdf]

Compound ID: 00000000

EB2224-64-P1A DMSO Bruker_NT-B_400MHZ

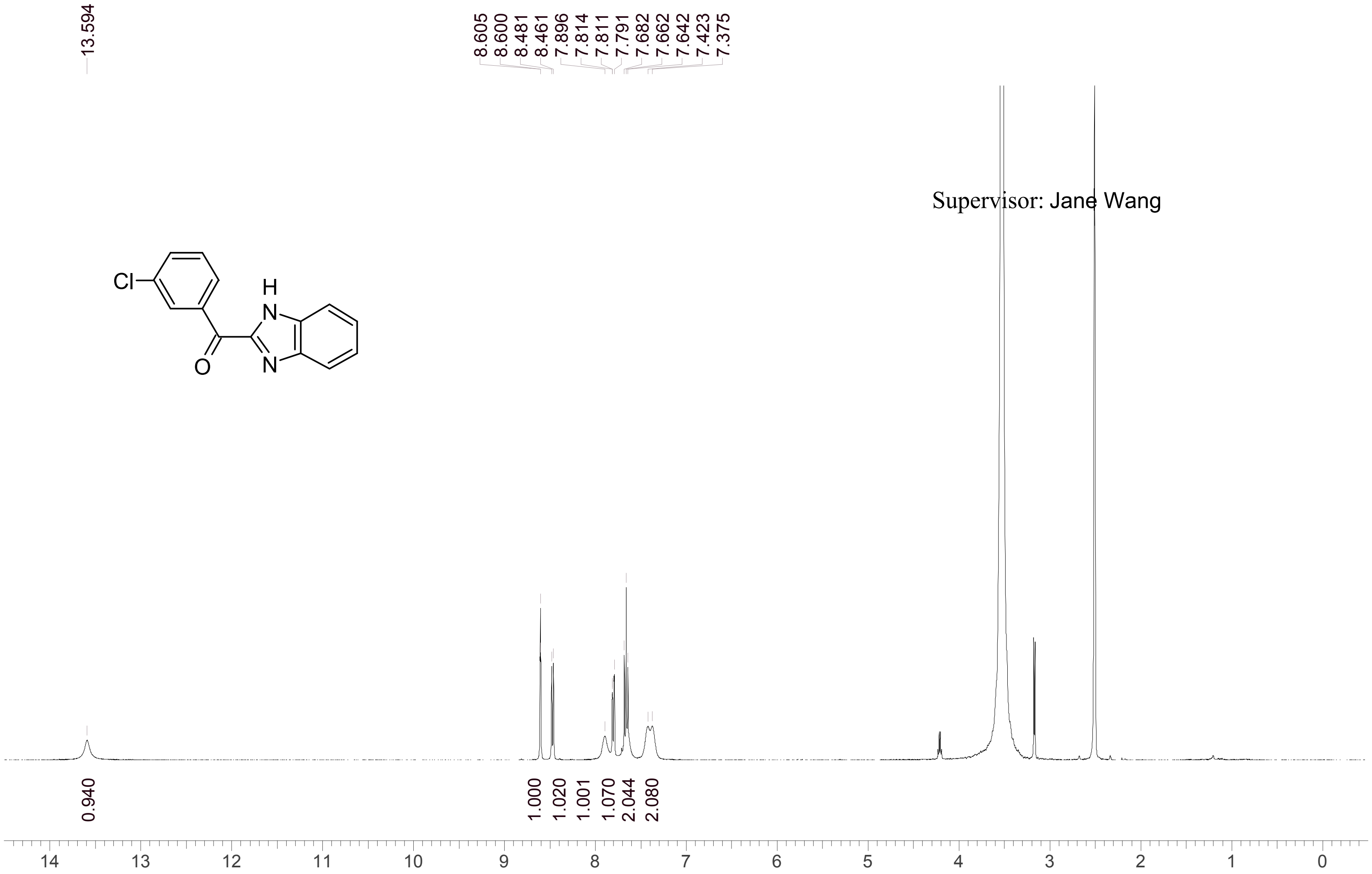

Supervisor: Jane Wang

Acquisition Time (sec)	3.9977
Comment	EB2224-64-P1A DMSO Bruker_NT-B_400MHZ
Date	10 Aug 2020 07:54:34
Frequency (MHz)	400.1300
Nucleus	1H
Number of Transients	8
Origin	Avance
Original Points Count	32768
Owner	nmrsu
Points Count	65536
Pulse Sequence	zg30
Receiver Gain	53.33
SW(cyclical) (Hz)	8196.72
Solvent	DMSO-d6
Spectrum Offset (Hz)	2470.8015
Spectrum Type	standard
Sweep Width (Hz)	8196.60
Temperature (degree C)	25.637

^{1}H NMR (400MHz, DMSO-d_6) δ = 13.59 (br s, 1H), 8.60 (t, *J*=1.8 Hz, 1H), 8.47 (d, *J*=7.9 Hz, 1H), 7.90 (br s, 1H), 7.82 - 7.78 (m, 1H), 7.66 (t, *J*=7.9 Hz, 2H), 7.40 (br d, *J*=19.4 Hz, 2H)

Confidential. For research only Not for regulatory filing

Operator:

Date:
