# Supplementary material for: Discovery of SARS-CoV-2 main protease inhibitors using a synthesis-directed de novo design model
Source: Chem Commun (Camb). 2021 May 6;57(48):5909–12. doi: 10.1039/d1cc00050k (PMC8204246; doi:10.1039/d1cc00050k)

CompoundID: ALP-POS-ddb41b15-1

EB1773-69-P1A3 CDCl3 Bruker_NT-C_400MHz

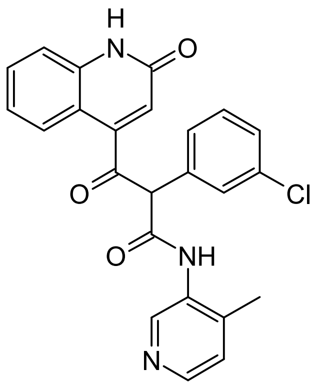

Exact Mass: 431.10

Supervisor:Jane Wang

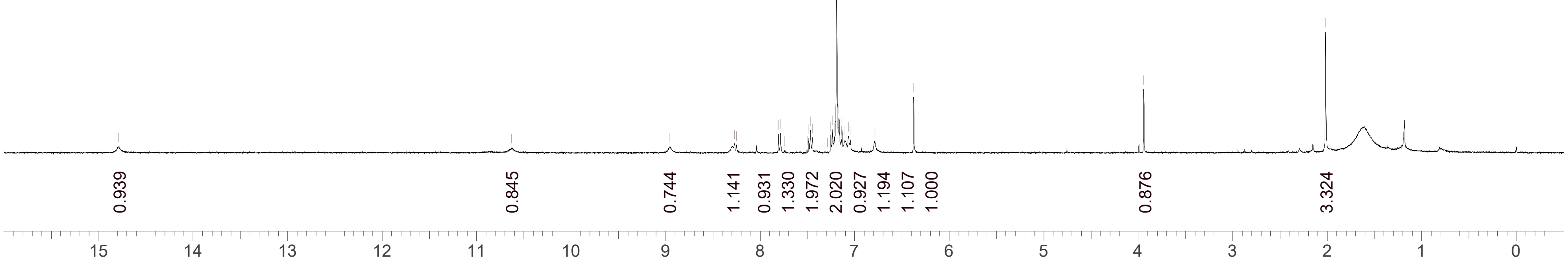

Confidential. For research only Not for regulatory filing

Operator:

Date:

Confidential. For research information only.

LCMS REPORT

Print time : 09/24/2020 15:45:09

Compound ID :1
Sample ID : EB1773-69-P1G
Injection Date :2020/9/24 15:40:46
Injection Vol : 4ul
Location :tray1 vail97
Acq Method :D:\method\10-80AB_4min_E_Shimadzu.lcm
Org DataFile :D:\DATA\2020\2009\200924\EB1773-69-P1G.lcd
Instrument & column:LCMS-SAP 1-2402
Xtimate C18, 3um,2.1*30mm

Chromatogram

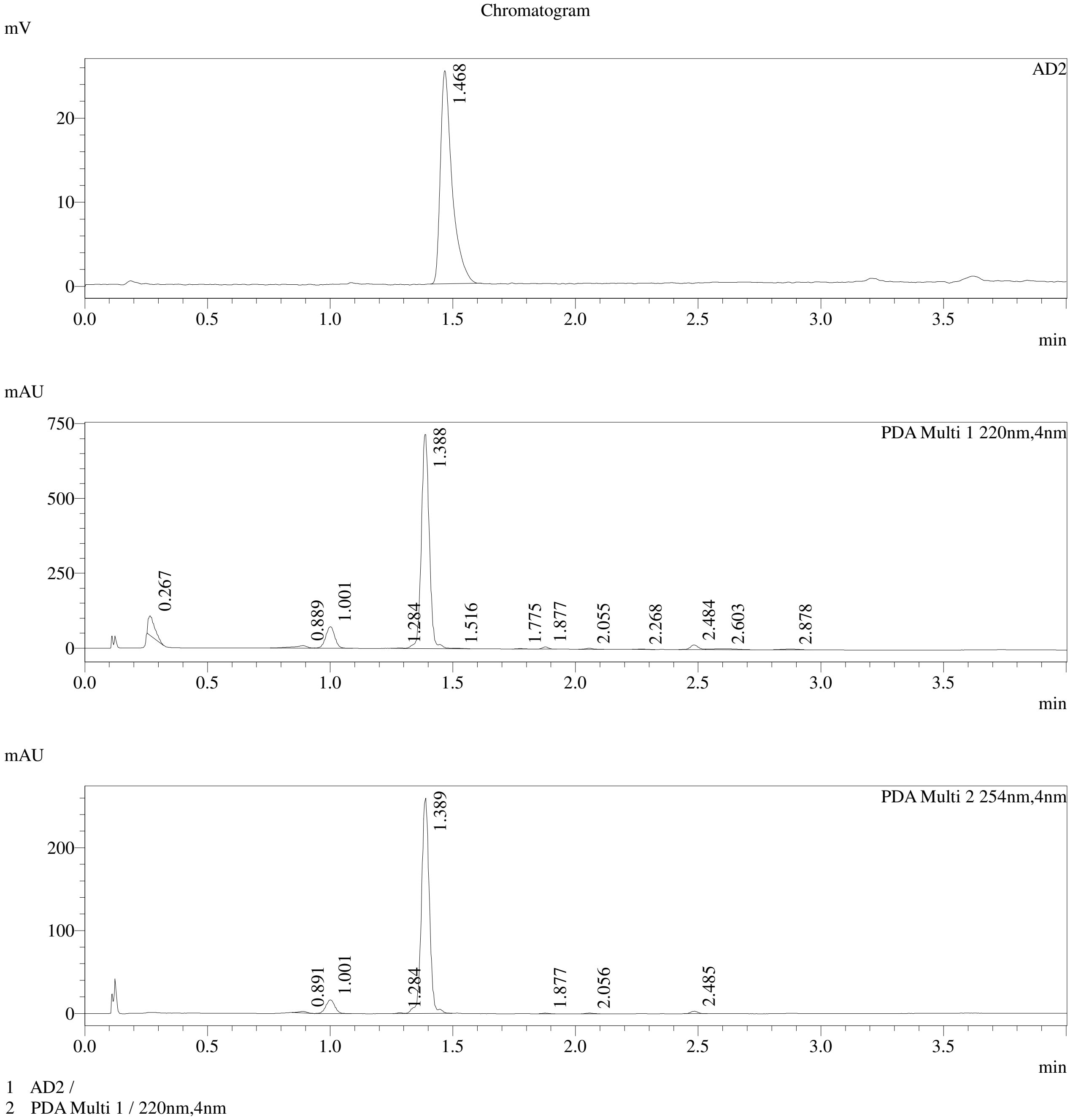

1 AD2 /
2 PDA Multi 1 / 220nm,4nm
3 PDA Multi 2 / 254nm,4nm

MS Chromatogram

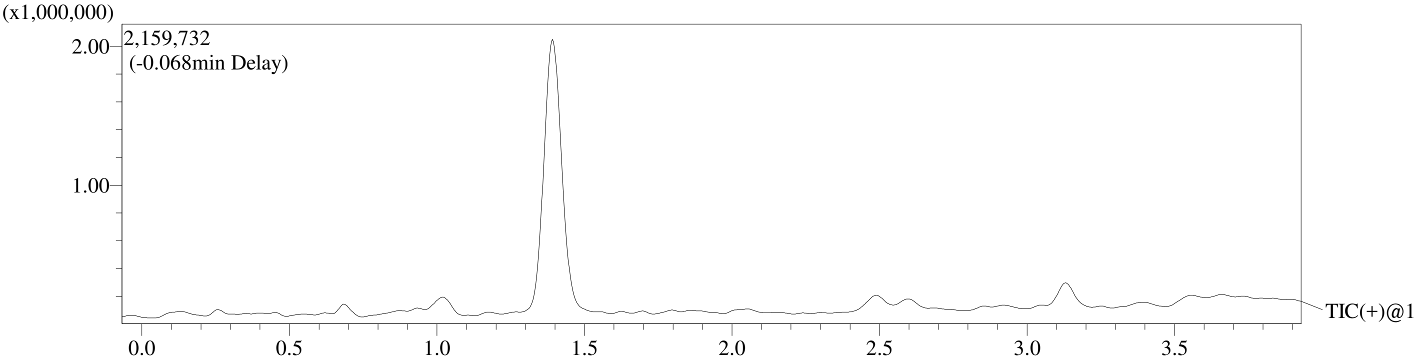

Integration Result

Peak Table

AD2

Peak#	Ret. Time	Height	Height%	USP Width	Area	Area%
1	1.468	25272	100.000	0.082	84353	100.000

PDA Ch1 220nm

Peak#	Ret. Time	Height	Height%	USP Width	Area	Area%
1	0.267	65118	7.246	0.057	122036	6.007
2	0.889	8443	0.939	0.088	34746	1.710
3	1.001	72467	8.064	0.070	184732	9.093
4	1.284	1775	0.197	0.051	2937	0.145
5	1.388	715571	79.625	0.060	1592783	78.405
6	1.516	839	0.093	0.041	1009	0.050
7	1.775	1360	0.151	0.062	3038	0.150
8	1.877	7375	0.821	0.049	12385	0.610
9	2.055	3325	0.370	0.057	6563	0.323
10	2.268	1149	0.128	0.067	2824	0.139
11	2.484	15667	1.743	0.059	35259	1.736
12	2.603	3089	0.344	0.207	21772	1.072
13	2.878	2505	0.279	0.127	11406	0.561

PDA Ch2 254nm

Peak#	Ret. Time	Height	Height%	USP Width	Area	Area%
1	0.891	2037	0.717	0.061	4258	0.673
2	1.001	16443	5.790	0.070	41907	6.620
3	1.284	932	0.328	0.046	1499	0.237
4	1.389	259916	91.519	0.061	576244	91.030
5	1.877	796	0.280	0.048	1302	0.206
6	2.056	936	0.330	0.056	1799	0.284
7	2.485	2942	1.036	0.056	6019	0.951

Operator:______________

Date:__________________

Mass Spectrum

RetTime: 0.265 Datafile: D:\DATA\2020\2009\200924\EB1773-69-P1G.lcd

Intensity

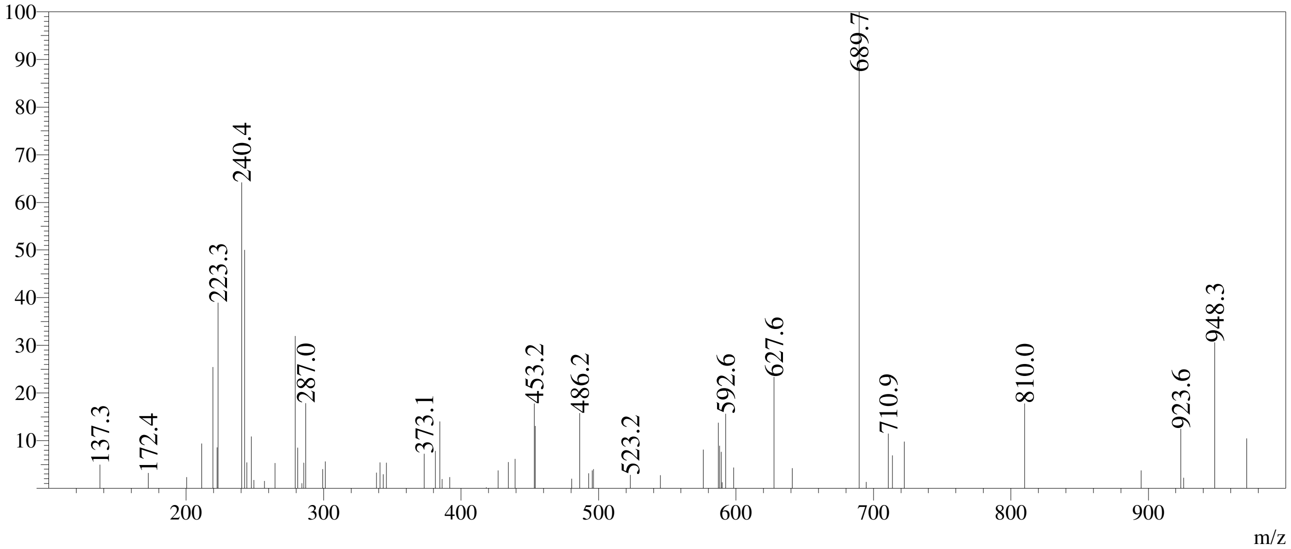

RetTime: 1.002 Datafile: D:\DATA\2020\2009\200924\EB1773-69-P1G.lcd

Intensity

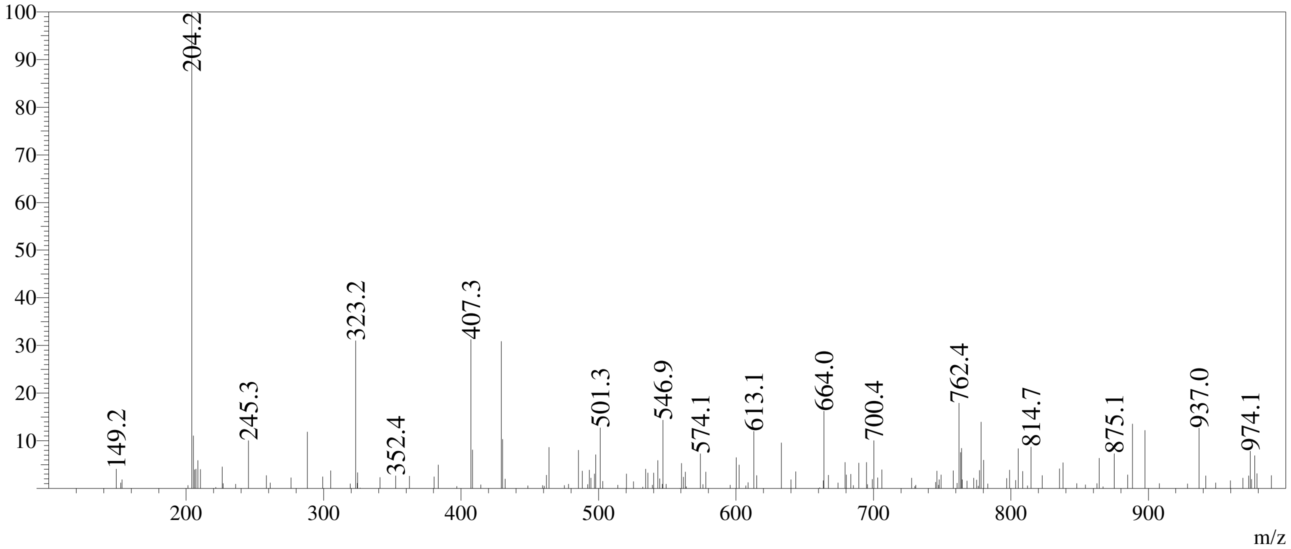

RetTime: 1.388 Datafile: D:\DATA\2020\2009\200924\EB1773-69-P1G.lcd

Intensity

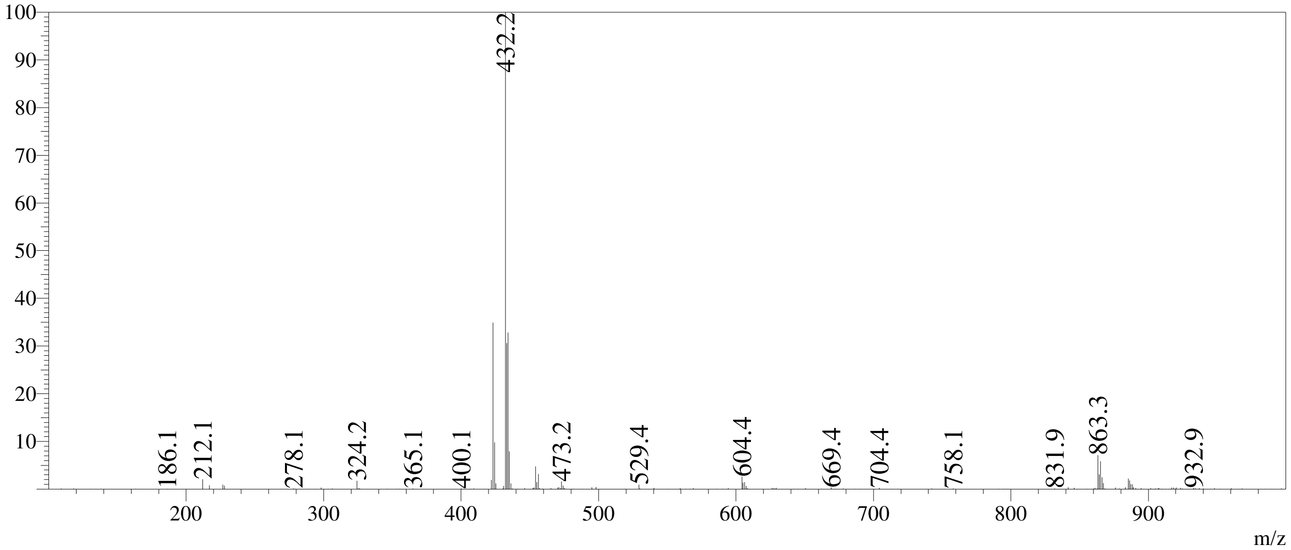

Supplement: CC-057-D1CC00050K-s031 [file CC-057-D1CC00050K-s031.pdf]
