# Supplementary material for: Discovery of SARS-CoV-2 main protease inhibitors using a synthesis-directed de novo design model
Source: Chem Commun (Camb). 2021 May 6;57(48):5909–12. doi: 10.1039/d1cc00050k (PMC8204246; doi:10.1039/d1cc00050k)

| | |
|---|---|
| Document Number: | EB2224-83B |
| Title: | EB2224-83-P1B1.pdf |
| Chemist: | ZHOU,PENG |
| Created Date: | Aug.20.2020 |
| Last Modified Date: | Aug.21.2020 |
| Witness: | Witnessed by CHEN, HUI on Aug.21.2020 |
| Print Date: | Nov.09.2020 |
| Copyright: | WuXi AppTec |
| Classifications: | Confidential, Vital Integrity |

[EB2224-83B] EB2224-83-P1B1.pdf 3

Confidential. For research information only.

# LCMS REPORT

Print time : 08/20/2020 18:01:57
Compound ID :1
Sample ID : EB2224-83-P1B1
Injection Date :2020/8/20 18:00:48
Injection Vol : 6ul
Location :tray1 vail40
Acq Method :5-95AB_1min_220&254_Shimadzu.lcm
Org Data File :D:\DATA\2020\2008\200820\EB2224-83-P1B1.lcd
Instrument & column:LCMS_05 1-2402
AB method: Chromolith @ Flash RP-18e,25-3mm
CD method: Xbridge shield RP 18, 5um,2.1*50mm

Chromatogram

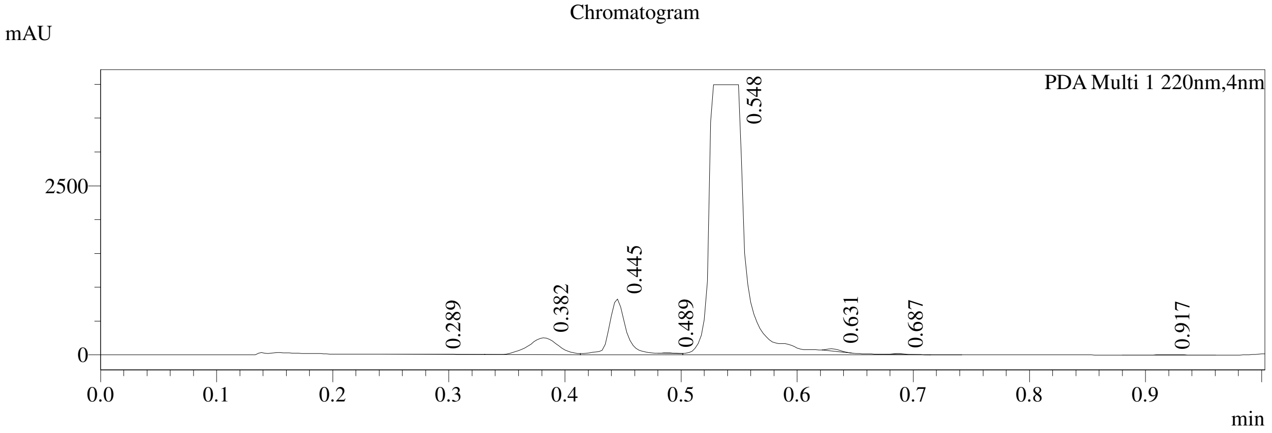


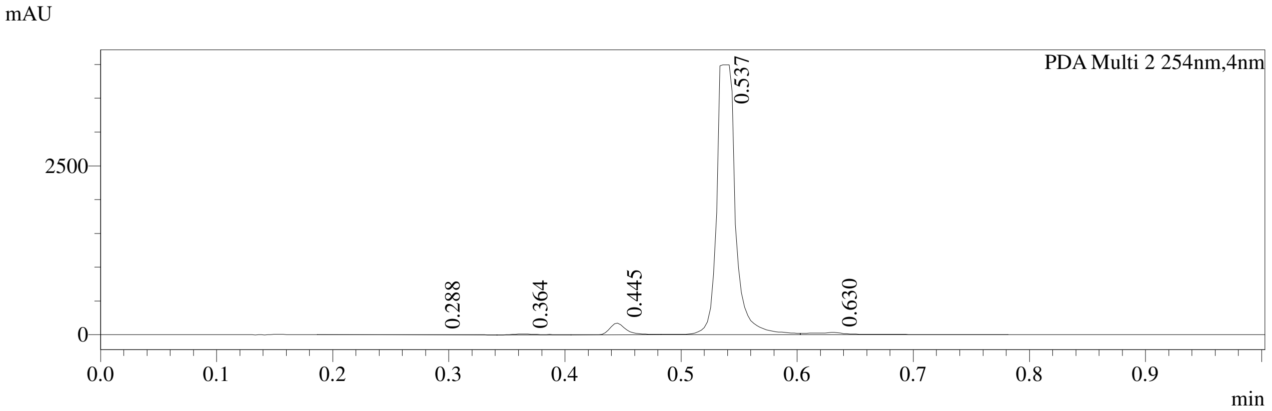


1 PDA Multi 1 / 220nm,4nm
2 PDA Multi 2 / 254nm,4nm

MS Chromatogram

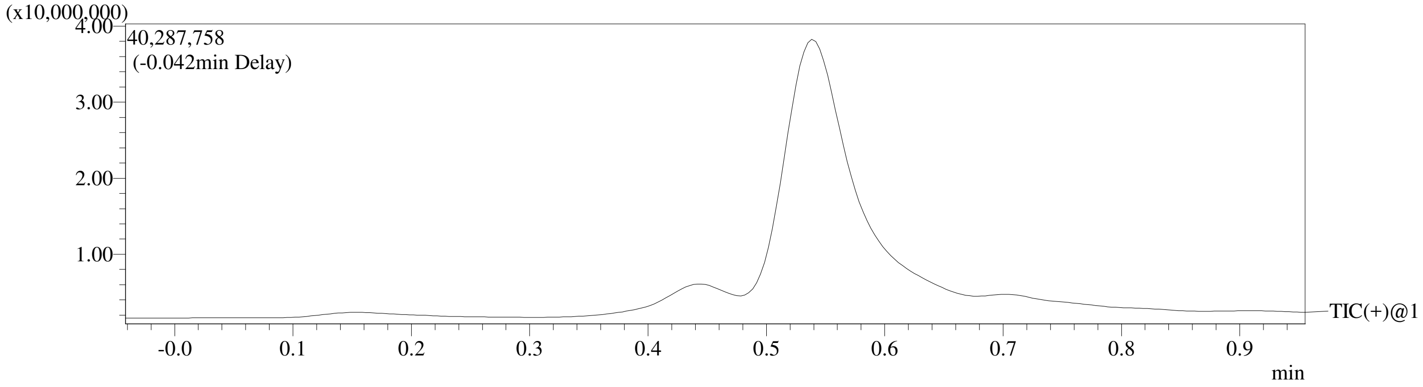


=====================================================================

Integration Result

=====================================================================

Peak Table

PDA Ch1 220nm

| Peak# | Ret. Time | Height | Height% | USP Width | Area | Area% |
|---|---|---|---|---|---|---|
| 1 | 0.289 | 5020 | 0.098 | 0.073 | 12222 | 0.125 |
| 2 | 0.382 | 248806 | 4.861 | 0.052 | 447313 | 4.571 |
| 3 | 0.445 | 821575 | 16.050 | 0.028 | 850010 | 8.686 |
| 4 | 0.489 | 5753 | 0.112 | 0.021 | 3654 | 0.037 |
| 5 | 0.548 | 3989803 | 77.943 | 0.040 | 8439220 | 86.236 |
| 6 | 0.631 | 33455 | 0.654 | 0.023 | 21241 | 0.217 |
| 7 | 0.687 | 13580 | 0.265 | 0.023 | 10331 | 0.106 |
| 8 | 0.917 | 854 | 0.017 | 0.073 | 2193 | 0.022 |

PDA Ch2 254nm

| Peak# | Ret. Time | Height | Height% | USP Width | Area | Area% |
|---|---|---|---|---|---|---|
| 1 | 0.288 | 3222 | 0.076 | 0.143 | 16983 | 0.353 |
| 2 | 0.364 | 13249 | 0.314 | 0.039 | 20134 | 0.418 |
| 3 | 0.445 | 173176 | 4.108 | 0.029 | 171896 | 3.569 |
| 4 | 0.537 | 3992825 | 94.710 | 0.029 | 4527750 | 94.001 |
| 5 | 0.630 | 33354 | 0.791 | 0.089 | 79965 | 1.660 |

Operator:______________

Date:__________________

Mass Spectrum

RetTime: 0.382  Datafile: D:\DATA\2020\2008\200820\EB2224-83-P1B1.lcd

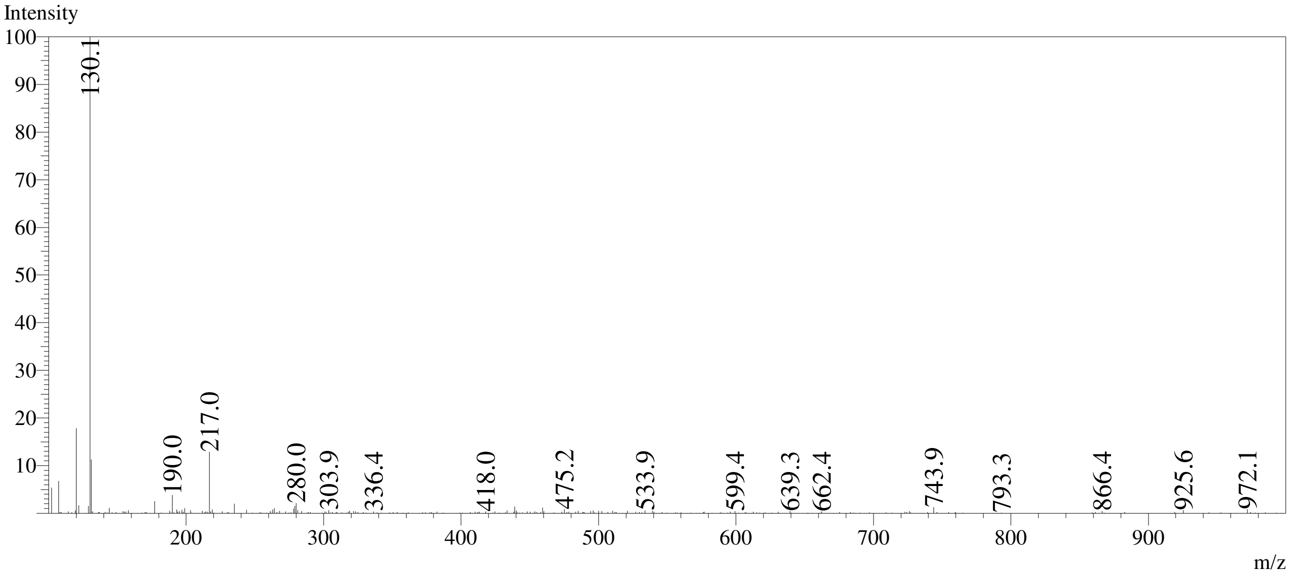


RetTime: 0.445  Datafile: D:\DATA\2020\2008\200820\EB2224-83-P1B1.lcd

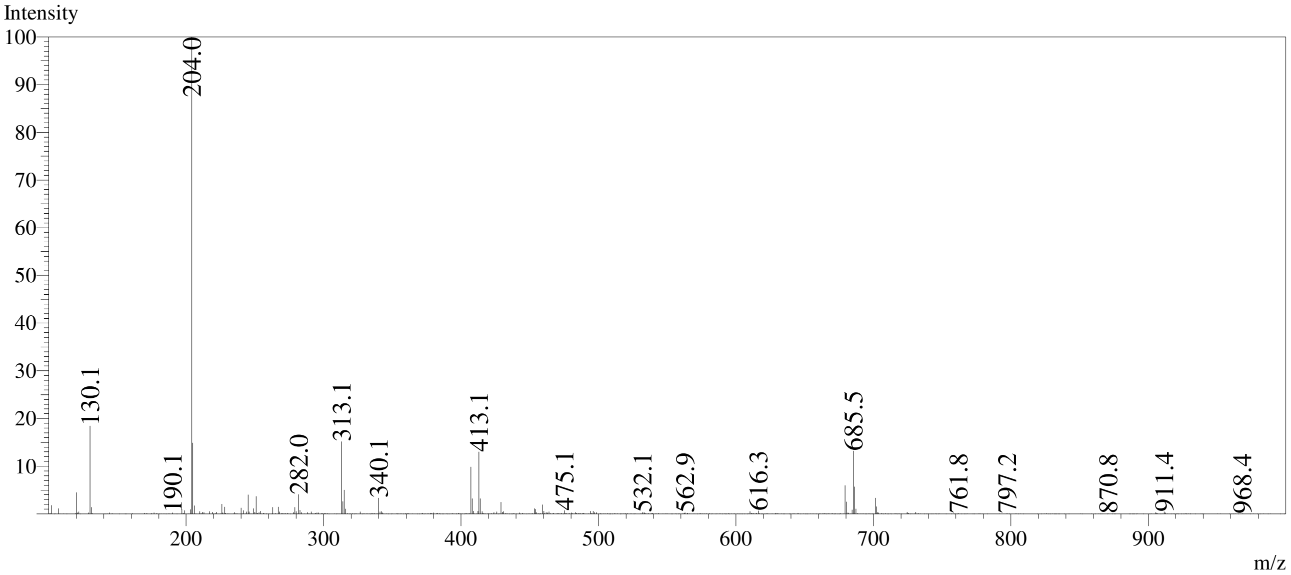


RetTime: 0.548  Datafile: D:\DATA\2020\2008\200820\EB2224-83-P1B1.lcd

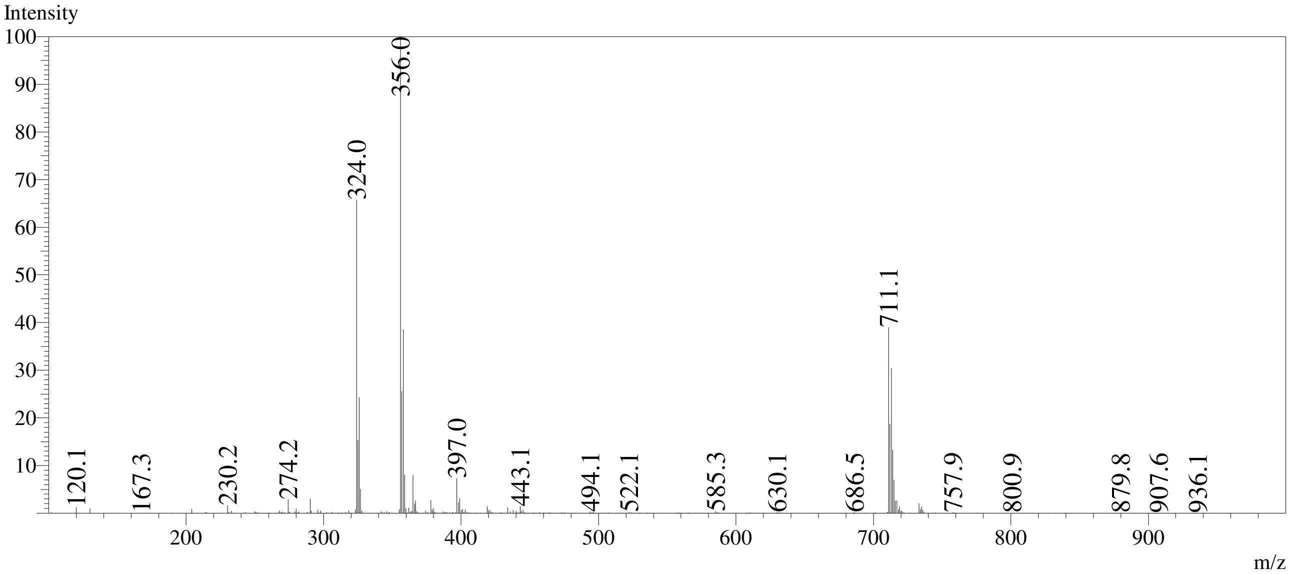

Supplement: CC-057-D1CC00050K-s033 [file CC-057-D1CC00050K-s033.pdf]
