# Supplementary material for: Discovery of SARS-CoV-2 main protease inhibitors using a synthesis-directed de novo design model
Source: Chem Commun (Camb). 2021 May 6;57(48):5909–12. doi: 10.1039/d1cc00050k (PMC8204246; doi:10.1039/d1cc00050k)
Supplement: CC-057-D1CC00050K-s037 [file CC-057-D1CC00050K-s037.pdf]

Document Number:	EB2224-108C
Title:	EB2224-108-P1A.pdf
Chemist:	ZHOU,PENG
Created Date:	Aug.28.2020
Last Modified Date:	Aug.31.2020
Witness:	Witnessed by CHEN, HUI on Aug.31.2020
Print Date:	Nov.09.2020
Copyright:	WuXi AppTec
Classifications:	Confidential, Vital Integrity

[EB2224-108C] EB2224-108-P1A.pdf 3

EB2224-108-P1A CDCl3 Bruker_NT-C_400MHz

WuXi AppTec
An Integrated R&D Service Company

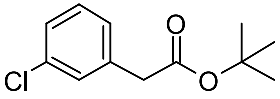

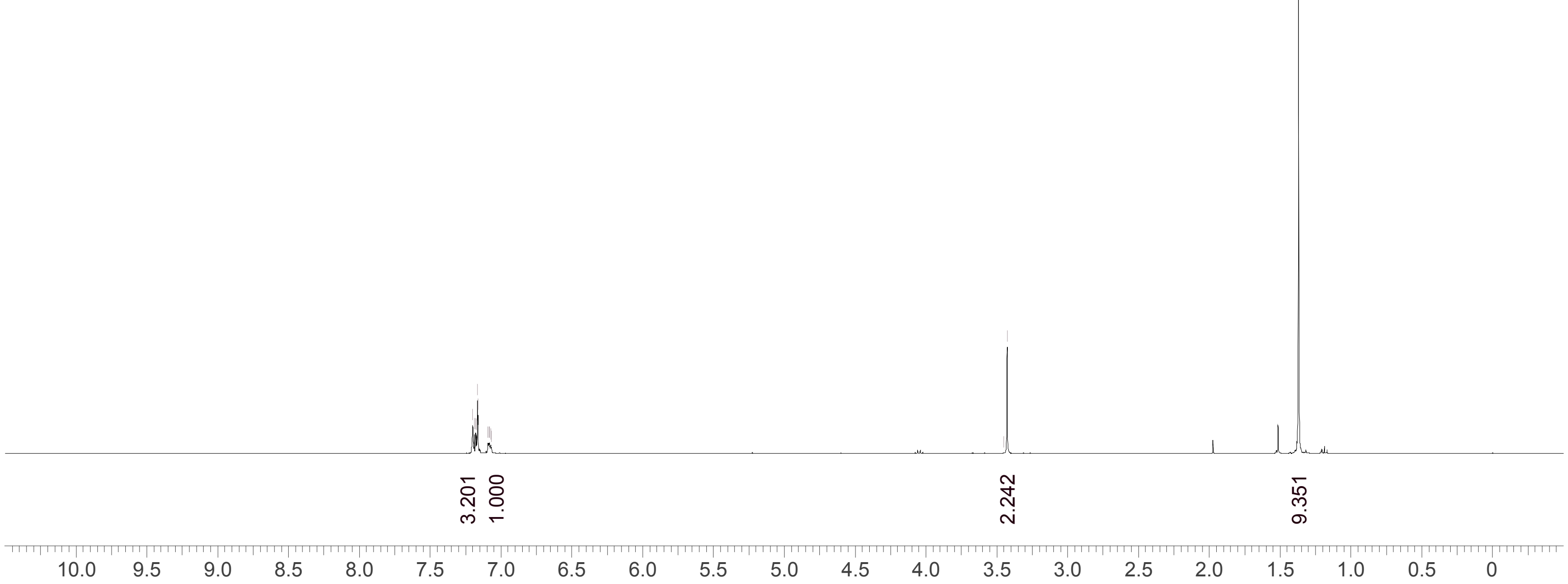

Supervisor: Jane Wang

Acquisition Time (sec)	1.9999
Comment	EB2224-108-P1A CDCl3 Bruker_NT-C_400MHz
Date	28 Aug 2020 08:33:59
Frequency (MHz)	400.1400
Nucleus	1H
Number of Transients	8
Origin	Avance
Original Points Count	16393
Owner	nmrsu
Points Count	65536
Pulse Sequence	zg30
Receiver Gain	101.00
SW(cyclical) (Hz)	8196.72
Solvent	CHLOROFORM-d
Spectrum Offset (Hz)	2362.7681
Spectrum Type	standard
Sweep Width (Hz)	8196.60
Temperature (degree C)	22.421

^{1}H NMR (400MHz, CHLOROFORM-d) δ = 7.22 - 7.16 (m, 3H), 7.10 - 7.06 (m, 1H), 3.45 - 3.41 (m, 2H), 1.37 (s, 9H)

Confidential. For research only Not for regulatory filing

Operator:

Date:
