# Supplementary material for: Discovery of SARS-CoV-2 main protease inhibitors using a synthesis-directed de novo design model
Source: Chem Commun (Camb). 2021 May 6;57(48):5909–12. doi: 10.1039/d1cc00050k (PMC8204246; doi:10.1039/d1cc00050k)

Document Number:	EB2224-124A
Title:	EB2224-124-P1A.pdf
Chemist:	ZHOU,PENG
Created Date:	Sep.03.2020
Last Modified Date:	Sep.04.2020
Witness:	Witnessed by CHEN, HUI on Sep.04.2020
Print Date:	Nov.10.2020
Copyright:	WuXi AppTec
Classifications:	Confidential, Vital Integrity

[EB2224-124A] EB2224-124-P1A.pdf 3

Confidential. For research information only.

LCMS REPORT

Print time : 09/03/2020 09:13:23
Compound ID :1
Sample ID : EB2224-124-P1A
Injection Date :2020/9/3 9:09:13
Injection Vol : 6ul
Location :tray1 vail3
Acq Method :0-30CD_4min_Pos_220&254_Shimadzu.lcm
Org Data File :D:\DATA\2020\2009\200903\EB2224-124-P1A.lcd
Instrument & column:LCMS_02 1-2402
XBridge Shield RP18, 5um,2.1*50mm

Chromatogram

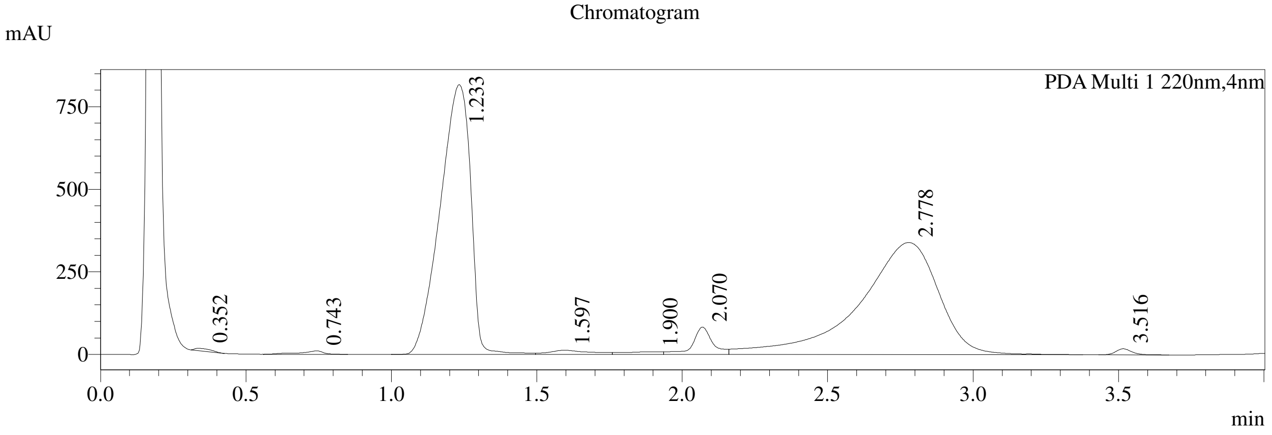

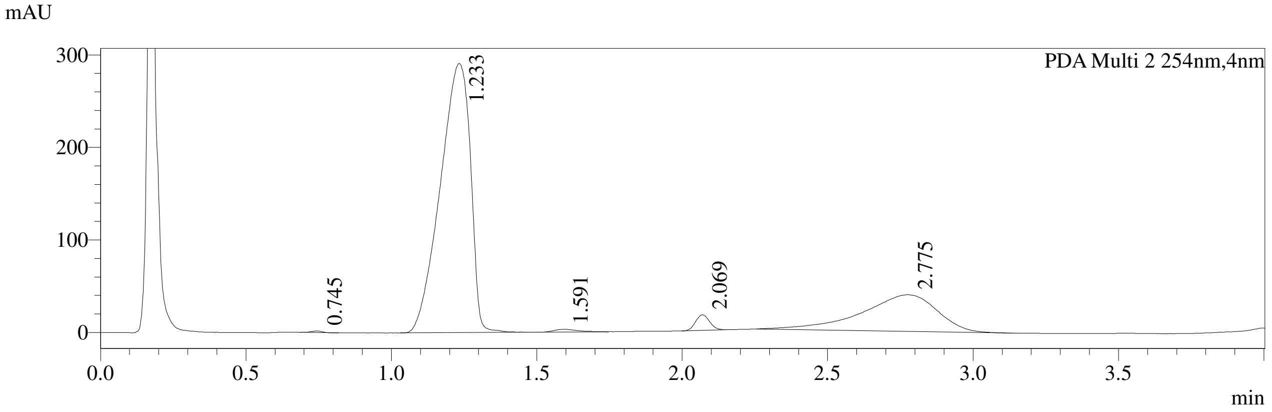

1 PDA Multi 1 / 220nm,4nm
2 PDA Multi 2 / 254nm,4nm

MS Chromatogram

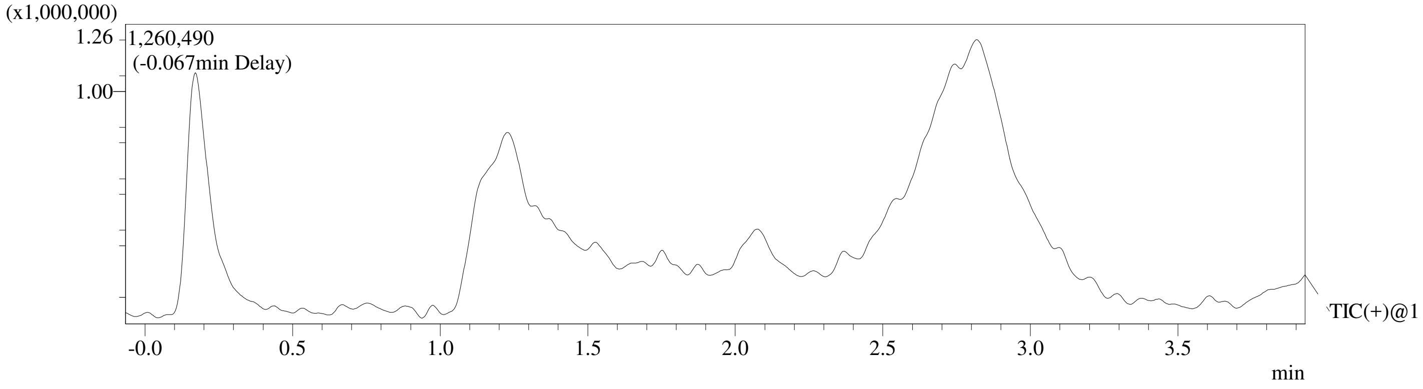

Integration Result

PDA Ch1 220nm

Peak#	Ret. Time	Height	Height%	USP Width	Area	Area%
1	0.352	7382	0.571	0.099	27113	0.206
2	0.743	10633	0.823	0.110	58510	0.444
3	1.233	813548	62.980	0.195	5970901	45.329
4	1.597	12710	0.984	0.375	124934	0.948
5	1.900	7841	0.607	10.807	73746	0.560
6	2.070	82532	6.389	0.104	392379	2.979
7	2.778	339106	26.252	0.459	6456975	49.019
8	3.516	18003	1.394	0.100	67870	0.515

PDA Ch2 254nm

Peak#	Ret. Time	Height	Height%	USP Width	Area	Area%
1	0.745	1732	0.493	0.072	4560	0.157
2	1.233	289979	82.634	0.194	2106967	72.701
3	1.591	2876	0.820	0.144	16518	0.570
4	2.069	16687	4.755	0.091	56215	1.940
5	2.775	39647	11.298	0.471	713879	24.632

Operator:______________

Date:__________________

Mass Spectrum

RetTime: 1.233 Datafile: D:\DATA\2020\2009\200903\EB2224-124-P1A.lcd

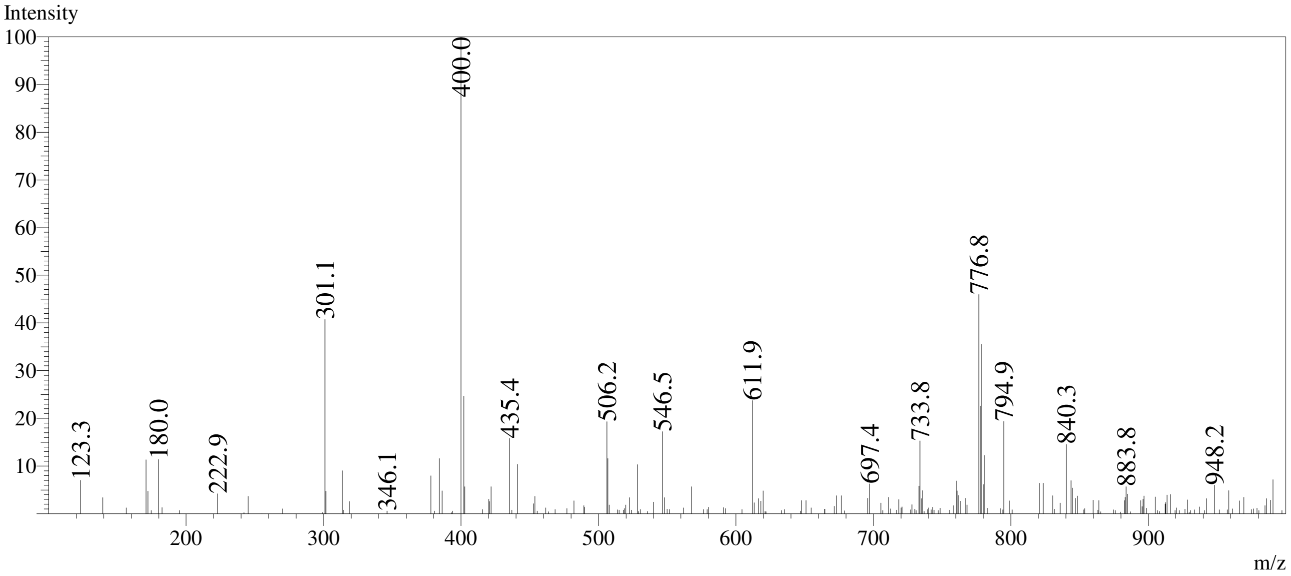

RetTime: 2.070 Datafile: D:\DATA\2020\2009\200903\EB2224-124-P1A.lcd

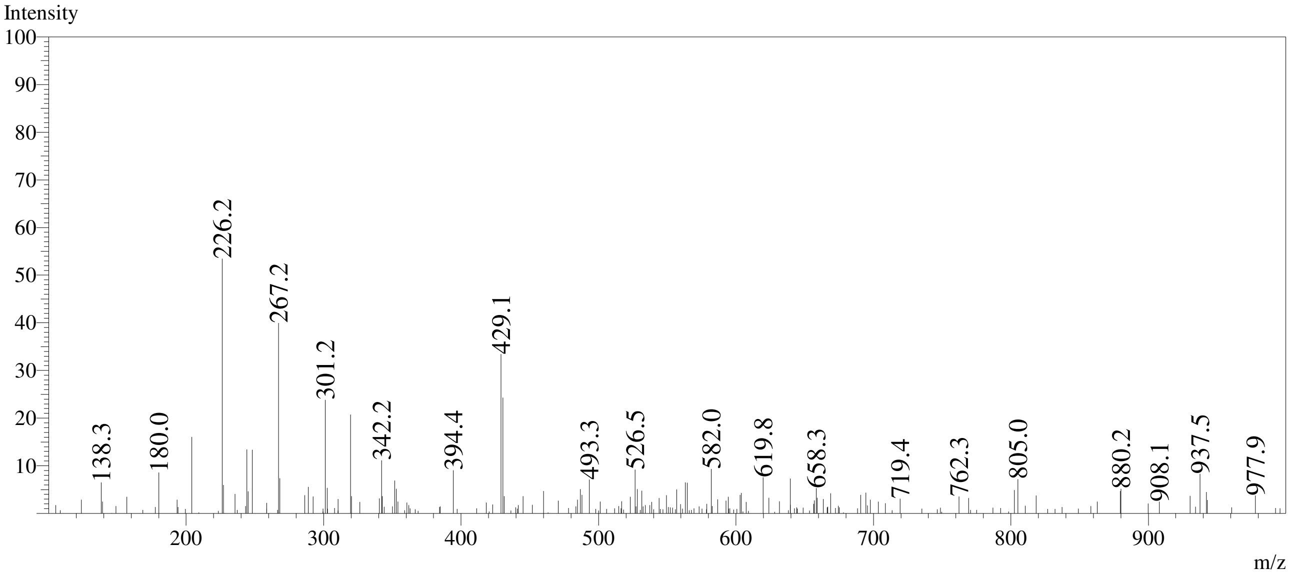

RetTime: 2.777 Datafile: D:\DATA\2020\2009\200903\EB2224-124-P1A.lcd

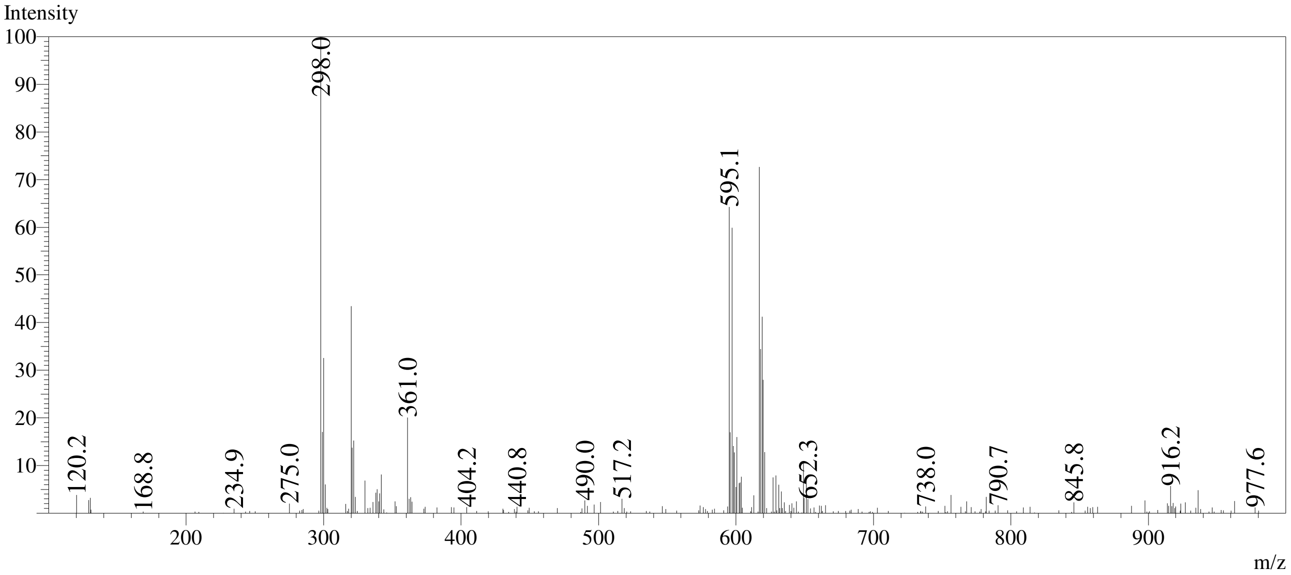

Supplement: CC-057-D1CC00050K-s039 [file CC-057-D1CC00050K-s039.pdf]
