# Supplementary material for: Discovery of SARS-CoV-2 main protease inhibitors using a synthesis-directed de novo design model
Source: Chem Commun (Camb). 2021 May 6;57(48):5909–12. doi: 10.1039/d1cc00050k (PMC8204246; doi:10.1039/d1cc00050k)

Document Number:	EB2224-120A
Title:	EB2224-120-P1B.pdf
Chemist:	ZHOU,PENG
Created Date:	Sep.03.2020
Last Modified Date:	Sep.07.2020
Witness:	Witnessed by CHEN, HUI on Sep.07.2020
Print Date:	Nov.10.2020
Copyright:	WuXi AppTec
Classifications:	Confidential, Vital Integrity

[EB2224-120A] EB2224-120-P1B.pdf 3

Confidential. For research information only.

LCMS REPORT

Print time : 09/03/2020 09:08:51
Compound ID :1
Sample ID : EB2224-120-P1B
Injection Date :2020/9/3 9:04:41
Injection Vol : 6ul
Location :tray1 vail2
Acq Method :0-30CD_4min_Pos_220&254_Shimadzu.lcm
Org Data File :D:\DATA\2020\2009\200903\EB2224-120-P1B.lcd
Instrument & column:LCMS_02 1-2402
XBridge Shield RP18, 5um,2.1*50mm

Chromatogram

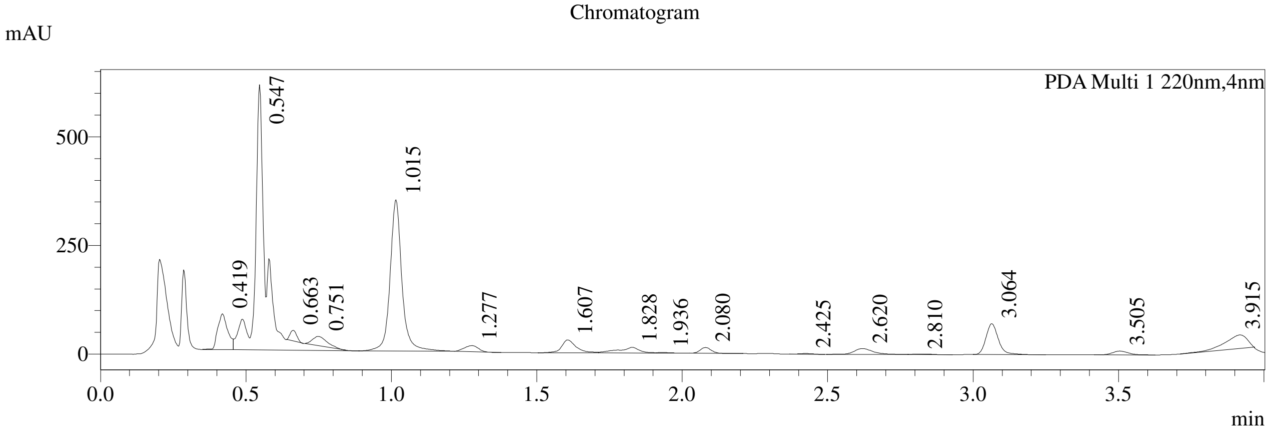

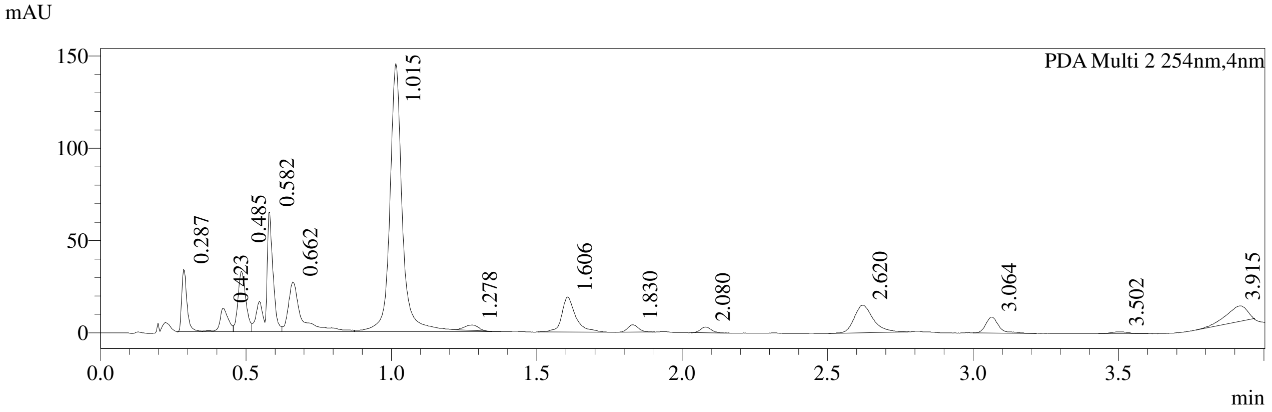

1 PDA Multi 1 / 220nm,4nm
2 PDA Multi 2 / 254nm,4nm

MS Chromatogram

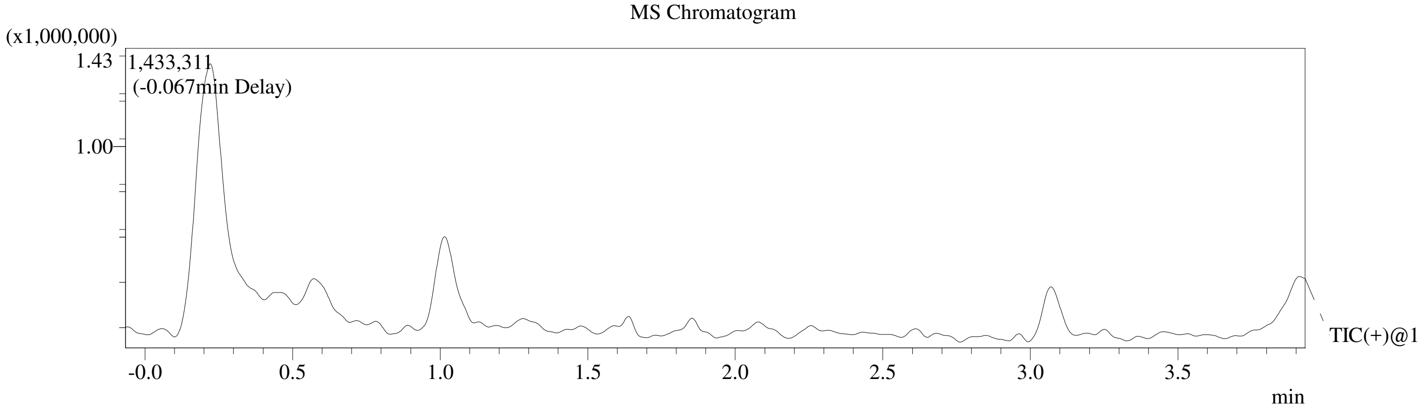

==

Integration Result

==

PDA Ch1 220nm

Peak#	Ret. Time	Height	Height%	USP Width	Area	Area%
1	0.419	80426	6.500	0.070	199757	5.471
2	0.547	572489	46.266	0.047	1615850	44.257
3	0.663	24209	1.956	0.046	37695	1.032
4	0.751	21526	1.740	0.093	73198	2.005
5	1.015	342178	27.653	0.072	985339	26.988
6	1.277	13902	1.123	0.093	45583	1.248
7	1.607	29591	2.391	0.087	105323	2.885
8	1.828	12807	1.035	0.098	65850	1.804
9	1.936	1260	0.102	0.097	3319	0.091
10	2.080	13040	1.054	0.072	34227	0.937
11	2.425	1586	0.128	0.085	4922	0.135
12	2.620	13469	1.089	0.115	61081	1.673
13	2.810	1110	0.090	0.098	4109	0.113
14	3.064	69779	5.639	0.079	210781	5.773
15	3.505	8706	0.704	0.101	33087	0.906
16	3.915	31320	2.531	0.143	170933	4.682

PDA Ch2 254nm

Peak#	Ret. Time	Height	Height%	USP Width	Area	Area%
1	0.287	32145	8.677	0.036	42169	4.217
2	0.423	12564	3.391	0.058	25845	2.584
3	0.485	32164	8.682	0.054	62266	6.226
4	0.582	62614	16.901	0.040	114199	11.419
5	0.662	26573	7.173	0.064	85338	8.533
6	1.015	142714	38.523	0.072	435627	43.559
7	1.278	2955	0.798	0.093	9805	0.980
8	1.606	18812	5.078	0.082	63637	6.363
9	1.830	3989	1.077	0.070	10278	1.028
10	2.080	3085	0.833	0.072	8212	0.821
11	2.620	14866	4.013	0.117	67847	6.784
12	3.064	8527	2.302	0.079	26618	2.662
13	3.502	933	0.252	0.105	3746	0.375
14	3.915	8527	2.302	0.142	44507	4.450

Operator:______________

Date:_________________

Mass Spectrum

RetTime: 0.420 Datafile: D:\DATA\2020\2009\200903\EB2224-120-P1B.lcd

Intensity

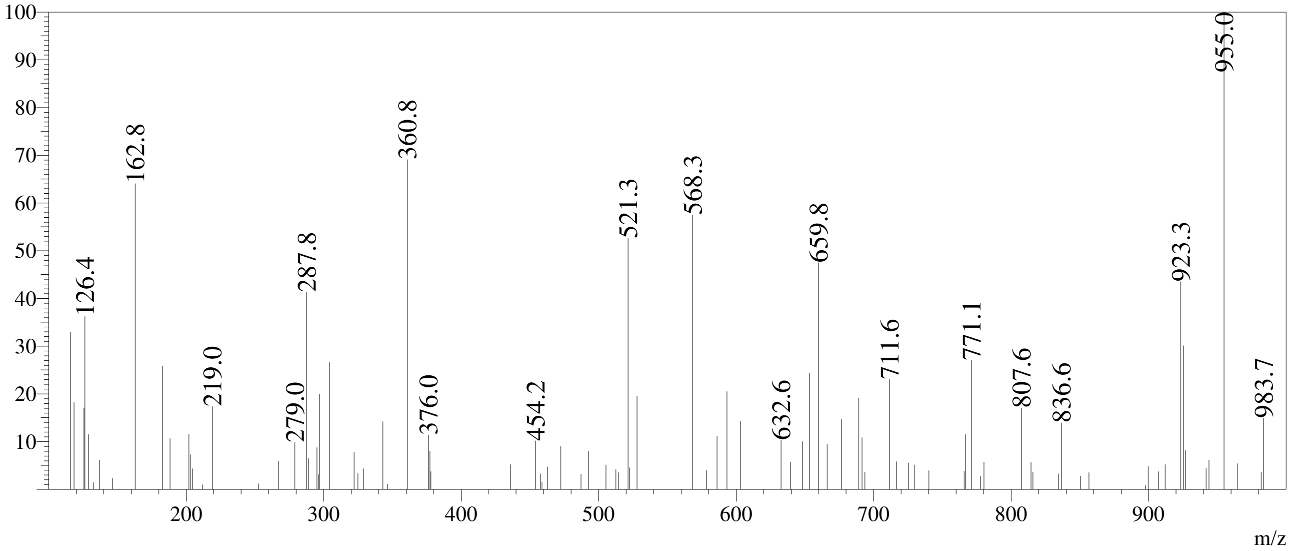

RetTime: 0.547 Datafile: D:\DATA\2020\2009\200903\EB2224-120-P1B.lcd

Intensity

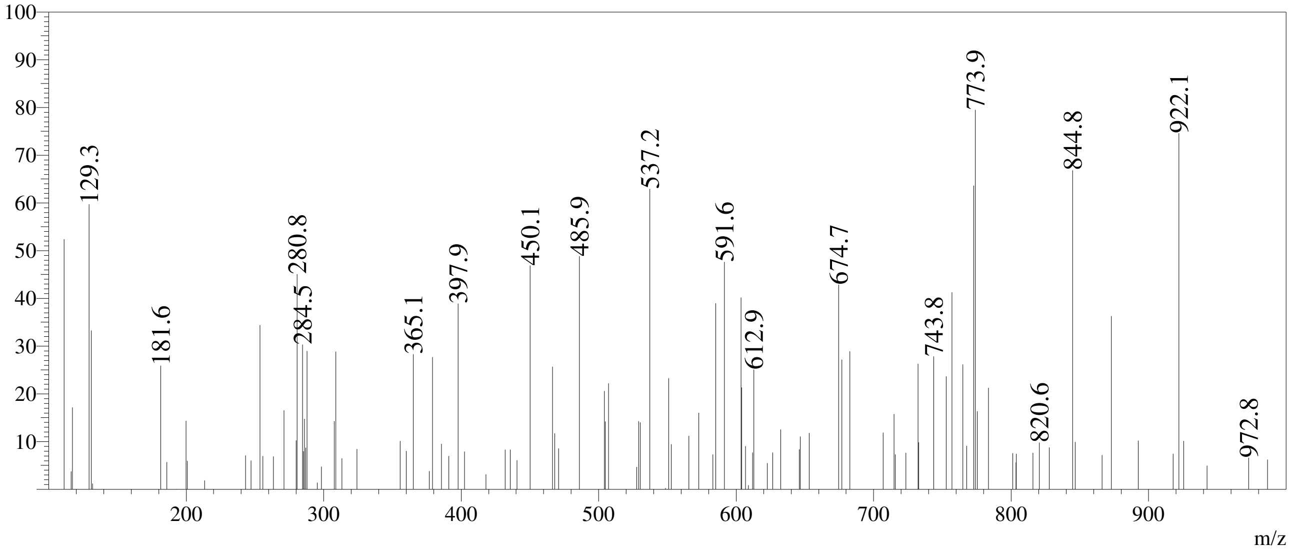

RetTime: 0.750 Datafile: D:\DATA\2020\2009\200903\EB2224-120-P1B.lcd

Intensity

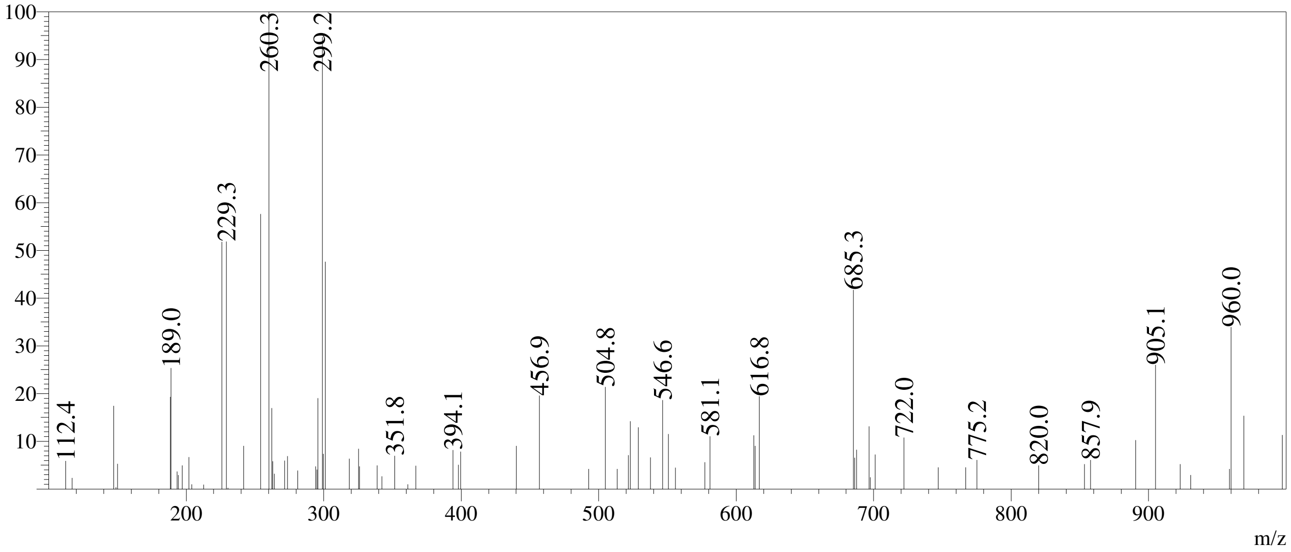

RetTime: 1.017 Datafile: D:\DATA\2020\2009\200903\EB2224-120-P1B.lcd

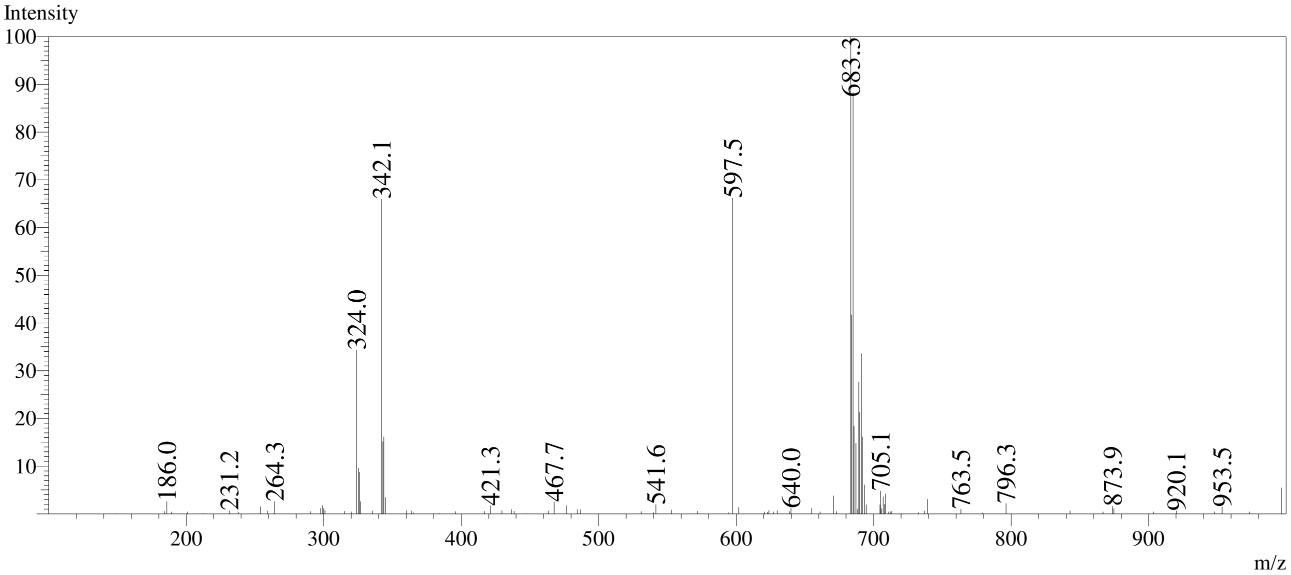

RetTime: 1.607 Datafile: D:\DATA\2020\2009\200903\EB2224-120-P1B.lcd

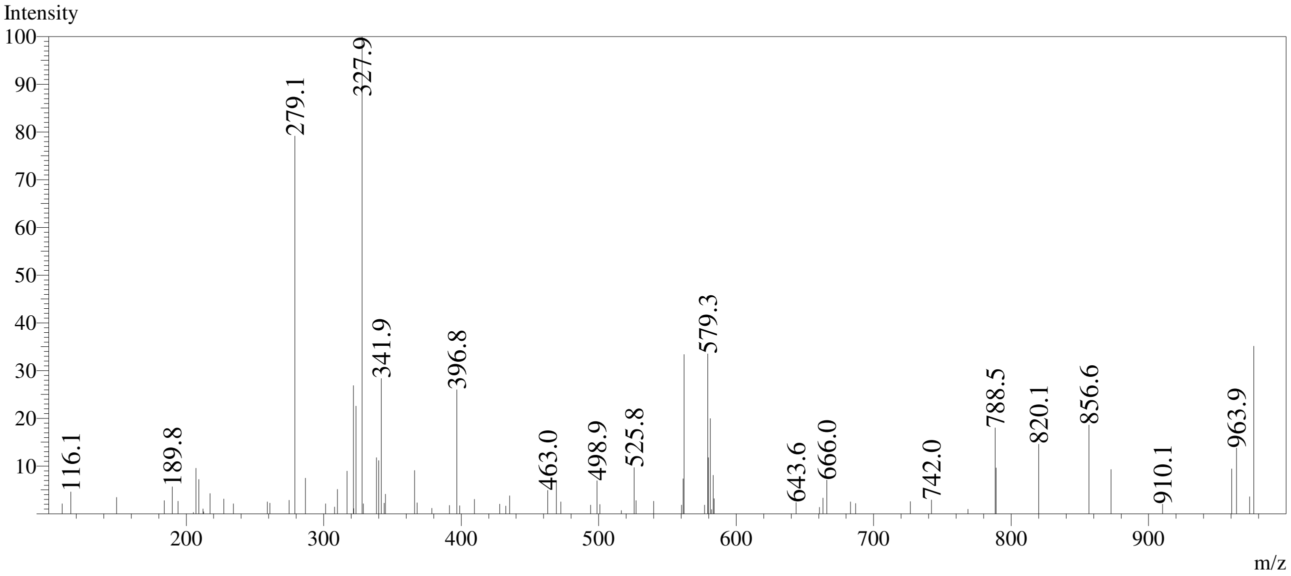

RetTime: 3.063 Datafile: D:\DATA\2020\2009\200903\EB2224-120-P1B.lcd

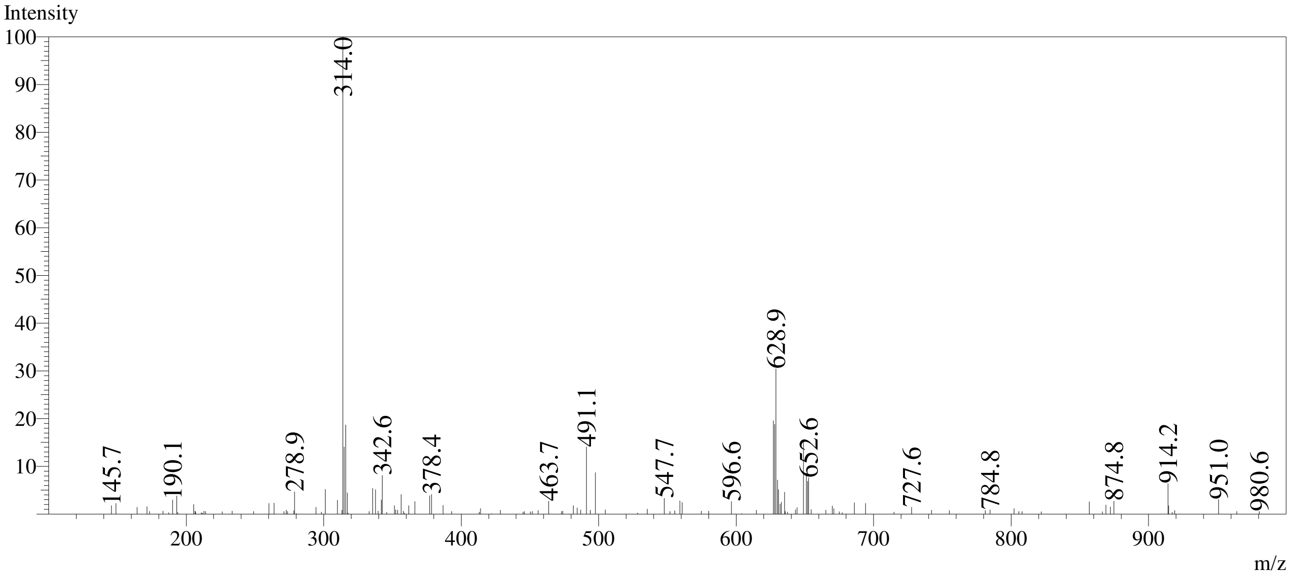

RetTime: 3.917 Datafile: D:\DATA\2020\2009\200903\EB2224-120-P1B.lcd

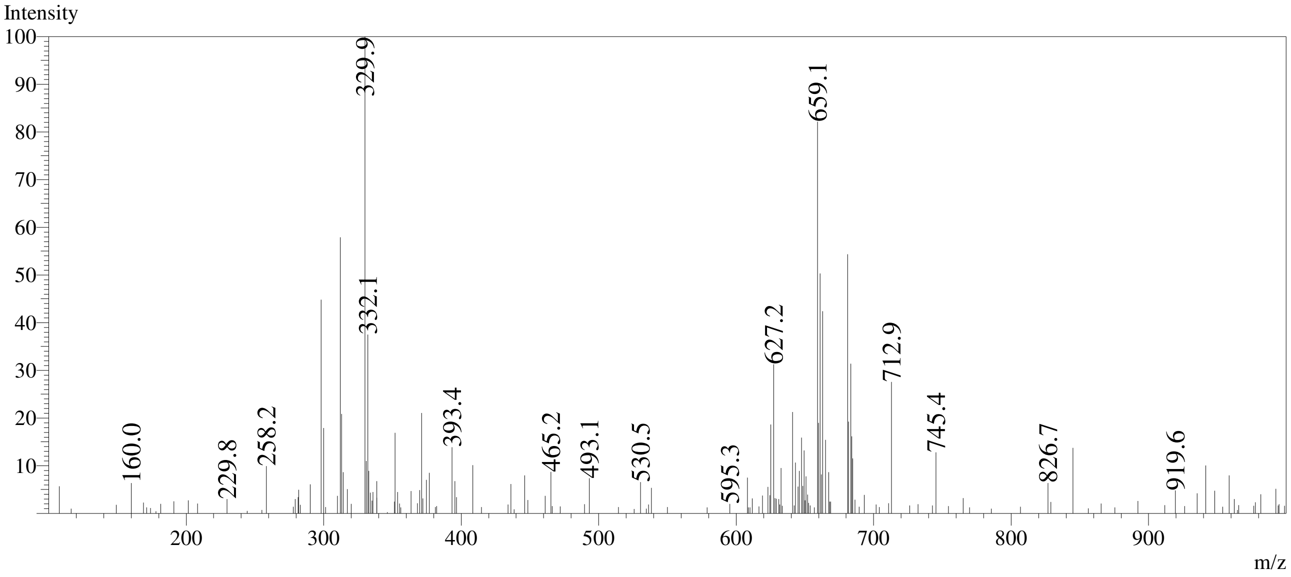

Supplement: CC-057-D1CC00050K-s041 [file CC-057-D1CC00050K-s041.pdf]
