# Supplementary material for: Discovery of SARS-CoV-2 main protease inhibitors using a synthesis-directed de novo design model
Source: Chem Commun (Camb). 2021 May 6;57(48):5909–12. doi: 10.1039/d1cc00050k (PMC8204246; doi:10.1039/d1cc00050k)
Supplement: CC-057-D1CC00050K-s043 [file CC-057-D1CC00050K-s043.pdf]

Document Number:	EB2224-114C
Title:	EB2224-114-P1A.pdf
Chemist:	ZHOU,PENG
Created Date:	Sep.01.2020
Last Modified Date:	Sep.03.2020
Witness:	Witnessed by CHEN, HUI on Sep.03.2020
Print Date:	Nov.10.2020
Copyright:	WuXi AppTec
Classifications:	Confidential, Vital Integrity

[EB2224-114C] EB2224-114-P1A.pdf 3

Compound ID: 00000000

EB2224-114-P1A DMSO Bruker_NT-C_400MHz

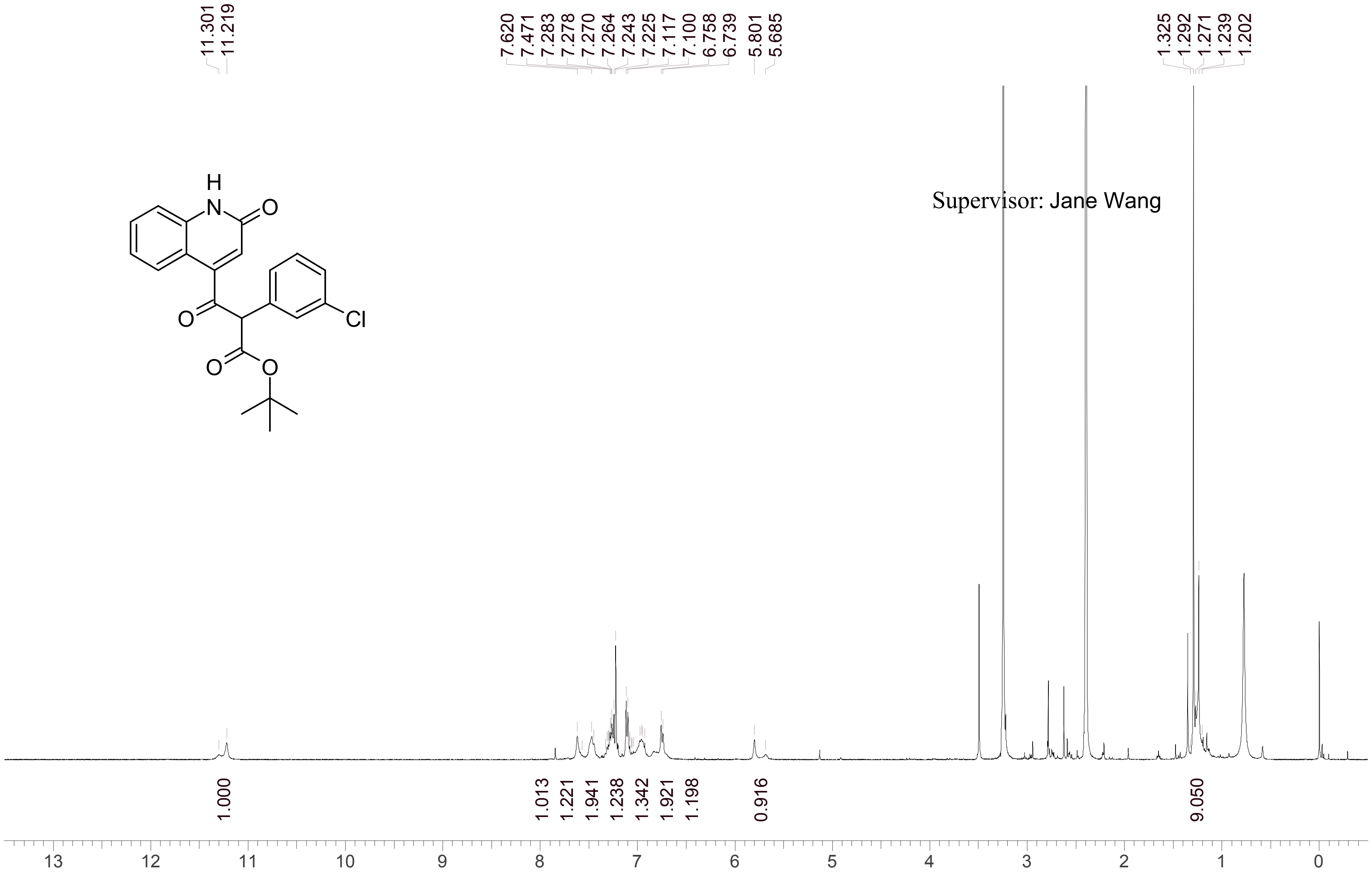

Acquisition Time (sec)	1.9999
Comment	EB2224-114-P1A DMSO Bruker_NT-C_400MHz
Date	01 Sep 2020 05:11:34
Frequency (MHz)	400.1400
Nucleus	1H
Number of Transients	8
Origin	Avance
Original Points Count	16393
Owner	nmrsu
Points Count	65536
Pulse Sequence	zg30
Receiver Gain	101.00
SW(cyclical) (Hz)	8196.72
Solvent	DMSO-d6
Spectrum Offset (Hz)	2355.9458
Spectrum Type	standard
Sweep Width (Hz)	8196.60
Temperature (degree C)	21.672

^{1}H NMR (400MHz, DMSO-d_6) δ = 11.40 - 11.11 (m, 1H), 7.67 - 7.53 (m, 1H), 7.46 (br d, *J*=8.5 Hz, 1H), 7.34 - 7.24 (m, 2H), 7.23 (s, 1H), 7.13 - 7.07 (m, 1H), 7.06 - 6.90 (m, 2H), 6.75 (br d, *J*=7.8 Hz, 1H), 5.88 - 5.61 (m, 1H), 1.33 - 1.20 (m, 9H)

Confidential. For research only Not for regulatory filing

Operator:

Date:
