# Supplementary material for: Discovery of SARS-CoV-2 main protease inhibitors using a synthesis-directed de novo design model
Source: Chem Commun (Camb). 2021 May 6;57(48):5909–12. doi: 10.1039/d1cc00050k (PMC8204246; doi:10.1039/d1cc00050k)

Compound ID: ALP-POS-ddb41b15-3

EB2224-194-P1A DMSO Bruker_NT-C_400MHz

药明康德 WuXi AppTec
An Integrated R&D Service Company

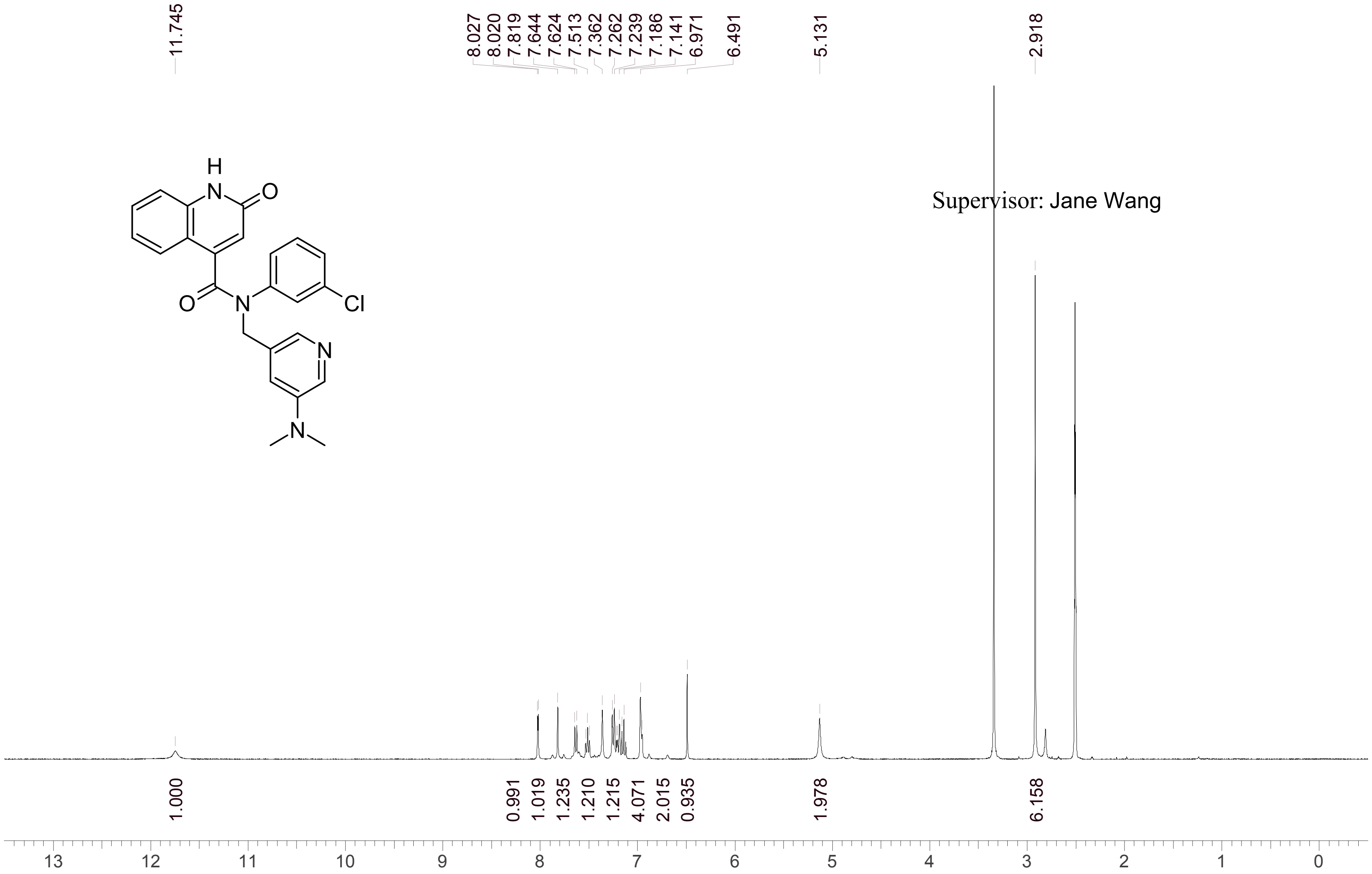

Acquisition Time (sec)	1.9999
Comment	EB2224-194-P1A DMSO Bruker_NT-C_400MHz
Date	30 Oct 2020 01:26:16
Frequency (MHz)	400.1400
Nucleus	1H
Number of Transients	8
Origin	Avance
Original Points Count	16393
Owner	nmrsu
Points Count	65536
Pulse Sequence	zg30
Receiver Gain	101.00
SW(cyclical) (Hz)	8196.72
Solvent	DMSO-d6
Spectrum Offset (Hz)	2400.8411
Spectrum Type	standard
Sweep Width (Hz)	8196.60
Temperature (degree C)	22.764

^{1}H NMR (400MHz, DMSO-d_6) δ = 11.74 (br s, 1H), 8.02 (d, *J*=2.8 Hz, 1H), 7.82 (s, 1H), 7.63 (d, *J*=8.0 Hz, 1H), 7.51 (t, *J*=7.4 Hz, 1H), 7.36 (s, 1H), 7.28 - 7.12 (m, 4H), 7.00 - 6.93 (m, 2H), 6.49 (s, 1H), 5.13 (br s, 2H), 2.92 (s, 6H)

Confidential. For research only Not for regulatory filing

Operator:

Date:

Confidential. For research information only.

LCMS REPORT

Print time : 10/29/2020 18:18:43
Compound ID :ALP-POS-ddb41b15-3
Sample ID : EB2224-194-P1B2
Injection Date :2020/10/29 18:14:28
Injection Vol : 1ul
Location :tray1 vail12
Acq Method :10-80CD_4min_Pos_220&254_Shimadzu.lcm
Org Data File :D:\DATA\2020\2010\201029\EB2224-194-P1B2.lcd
Instrument & column:LCMS_02 1-2402
XBridge Shield RP18, 5um,2.1*50mm

Chromatogram

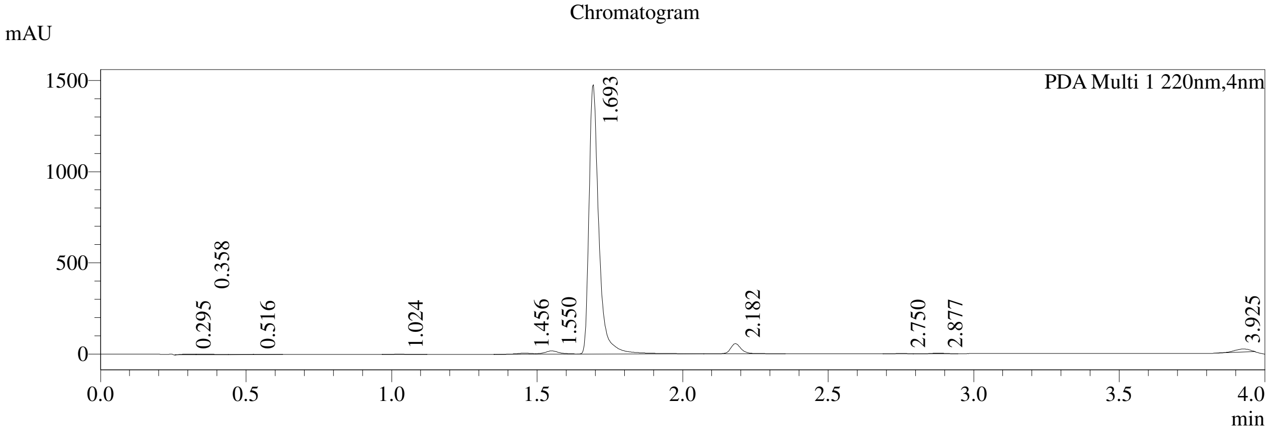

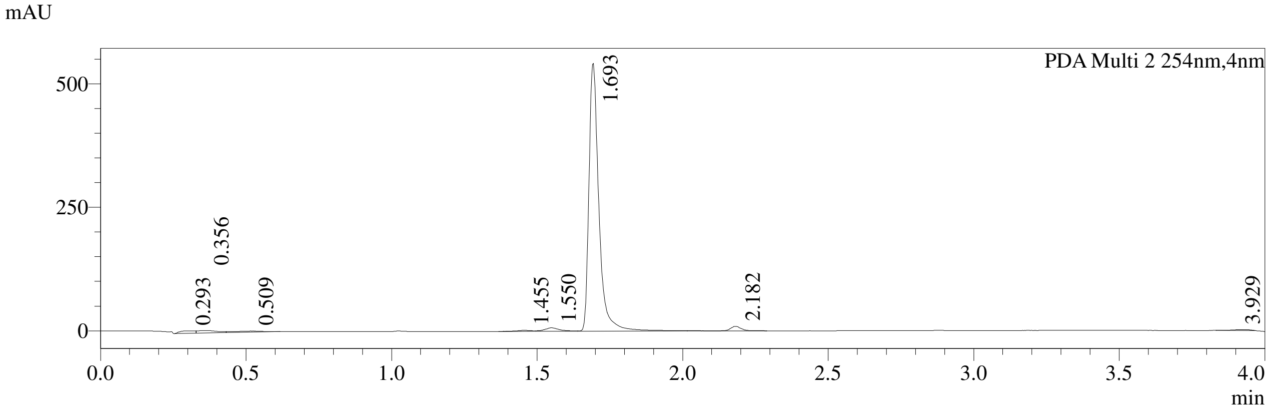

1 PDA Multi 1 / 220nm,4nm
2 PDA Multi 2 / 254nm,4nm

MS Chromatogram

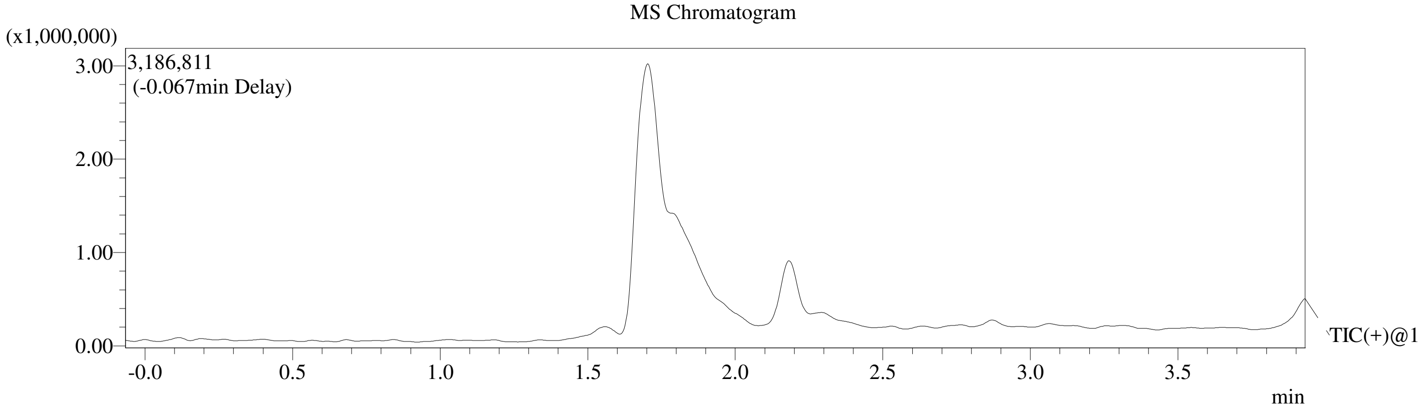

Integration Result

PDA Ch1 220nm

Peak#	Ret. Time	Height	Height%	USP Width	Area	Area%
1	0.295	3459	0.220	0.271	11704	0.304
2	0.358	3930	0.251	0.218	17915	0.466
3	0.516	1837	0.117	0.396	11620	0.302
4	1.024	2573	0.164	0.061	6823	0.177
5	1.456	6449	0.411	0.103	23216	0.603
6	1.550	18245	1.163	0.090	65605	1.705
7	1.693	1455765	92.789	0.062	3488328	90.667
8	2.182	55029	3.507	0.068	146903	3.818
9	2.750	956	0.061	0.062	2205	0.057
10	2.877	3733	0.238	0.069	9303	0.242
11	3.925	16917	1.078	0.100	63793	1.658

PDA Ch2 254nm

Peak#	Ret. Time	Height	Height%	USP Width	Area	Area%
1	0.293	5005	0.881	0.294	17922	1.263
2	0.356	5233	0.921	0.278	24221	1.707
3	0.509	2378	0.419	0.427	17034	1.200
4	1.455	2044	0.360	0.116	7688	0.542
5	1.550	7051	1.242	0.091	25676	1.809
6	1.693	535285	94.252	0.062	1293487	91.132
7	2.182	9066	1.596	0.069	25815	1.819
8	3.929	1866	0.328	0.105	7506	0.529

Operator:_______________

Date:___________________

Mass Spectrum

RetTime: 1.693 Datafile: D:\DATA\2020\2010\201029\EB2224-194-P1B2.lcd

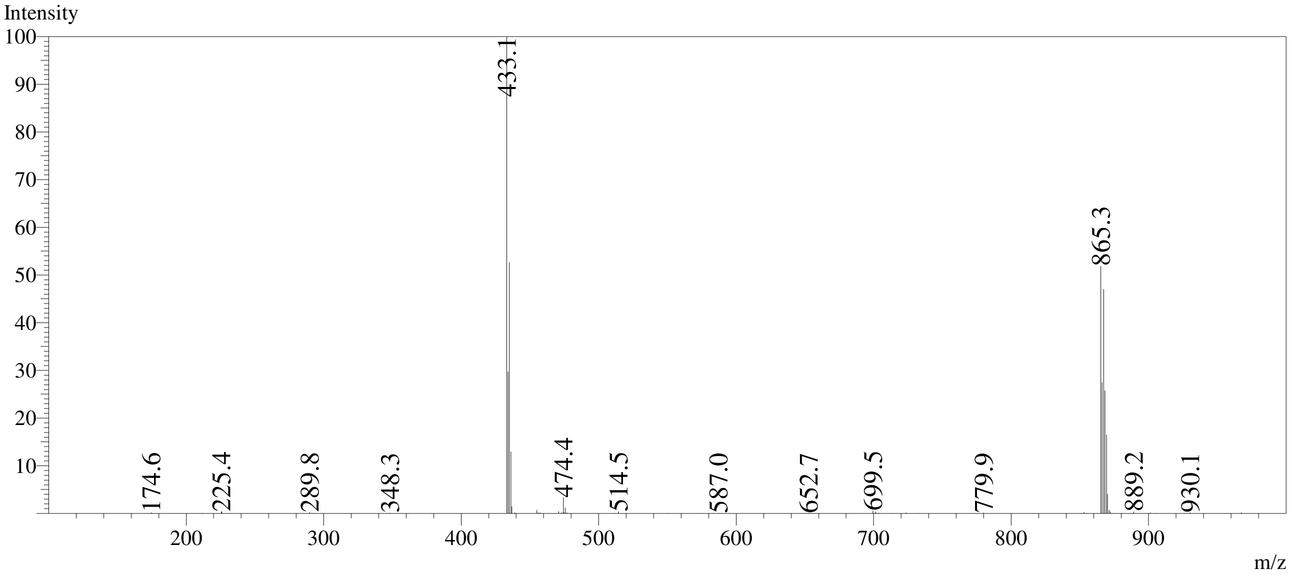

RetTime: 2.180 Datafile: D:\DATA\2020\2010\201029\EB2224-194-P1B2.lcd

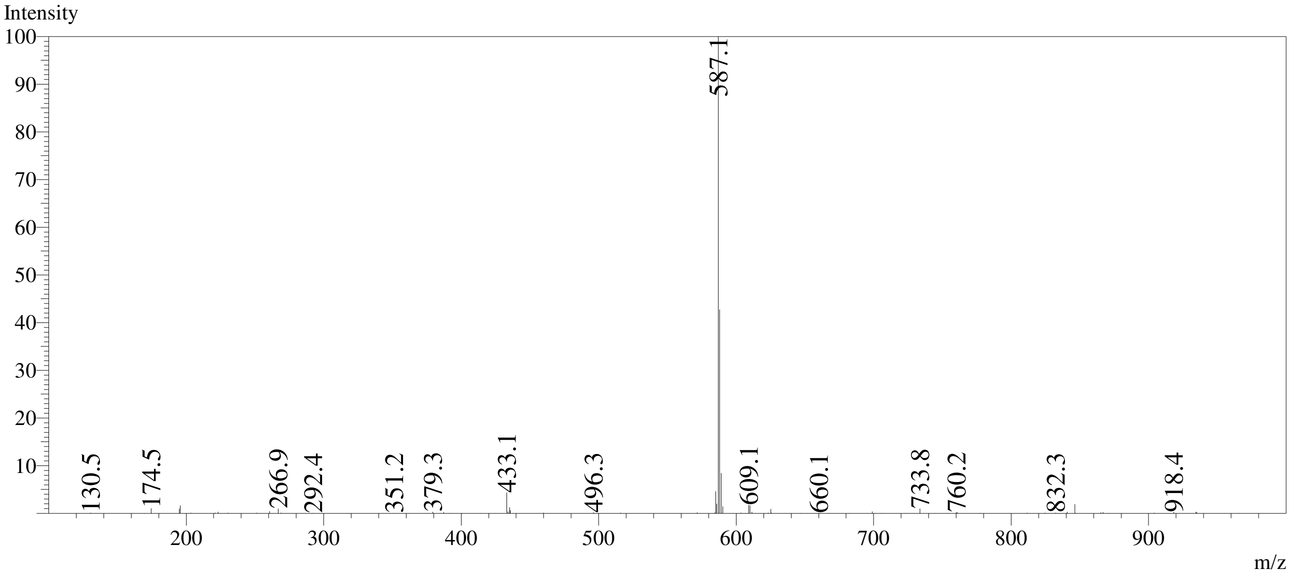

Supplement: CC-057-D1CC00050K-s045 [file CC-057-D1CC00050K-s045.pdf]
