# Supplementary material for: Discovery of SARS-CoV-2 main protease inhibitors using a synthesis-directed de novo design model
Source: Chem Commun (Camb). 2021 May 6;57(48):5909–12. doi: 10.1039/d1cc00050k (PMC8204246; doi:10.1039/d1cc00050k)
Supplement: CC-057-D1CC00050K-s053 [file CC-057-D1CC00050K-s053.pdf]

Compound ID: 00000000

EB2224-140-P1A CDCl3 Bruker_NT-C_400MHz

WuXi AppTec
An Integrated R&D Service Company

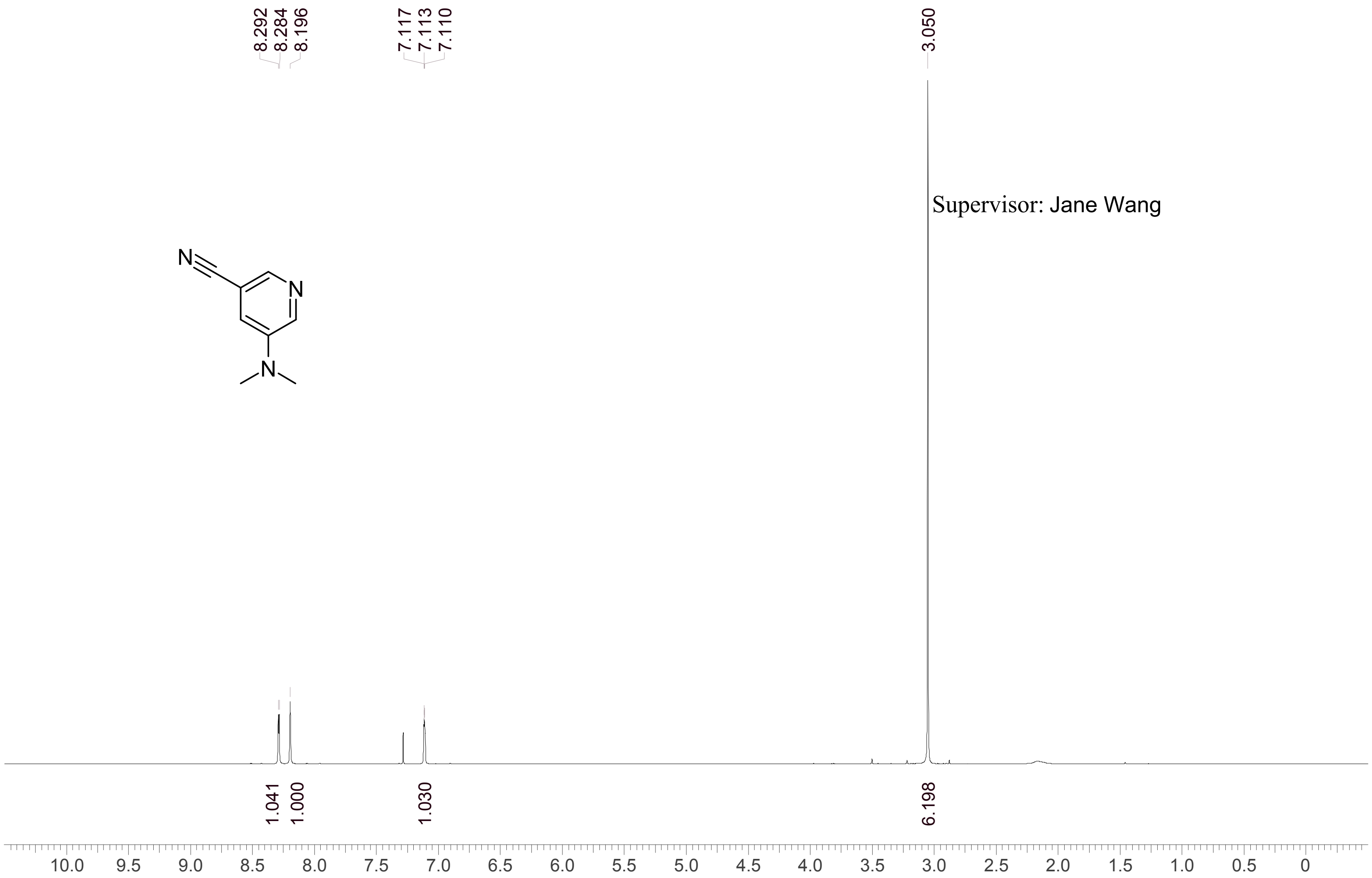

Acquisition Time (sec)	1.9999
Comment	EB2224-140-P1A CDCl3 Bruker_NT-C_400MHz
Date	23 Sep 2020 12:06:33
Frequency (MHz)	400.1400
Nucleus	1H
Number of Transients	8
Origin	Avance
Original Points Count	16393
Owner	nmrsu
Points Count	65536
Pulse Sequence	zg30
Receiver Gain	101.00
SW(cyclical) (Hz)	8196.72
Solvent	CHLOROFORM-d
Spectrum Offset (Hz)	2400.8411
Spectrum Type	standard
Sweep Width (Hz)	8196.60
Temperature (degree C)	23.386

^{1}H NMR (400MHz, CHLOROFORM-d) δ = 8.29 (d, *J*=3.0 Hz, 1H), 8.20 (s, 1H), 7.11 (dd, *J*=1.6, 2.8 Hz, 1H), 3.05 (s, 6H)

Confidential. For research only Not for regulatory filing

Operator:

Date:
