# Supplementary material for: Discovery of SARS-CoV-2 main protease inhibitors using a synthesis-directed de novo design model
Source: Chem Commun (Camb). 2021 May 6;57(48):5909–12. doi: 10.1039/d1cc00050k (PMC8204246; doi:10.1039/d1cc00050k)
Supplement: CC-057-D1CC00050K-s055 [file CC-057-D1CC00050K-s055.pdf]

Compound ID: 00000000

EB2224-187-P1A DMSO Bruker_NT-C_400MHz

WuXi AppTec
An Integrated R&D Service Company

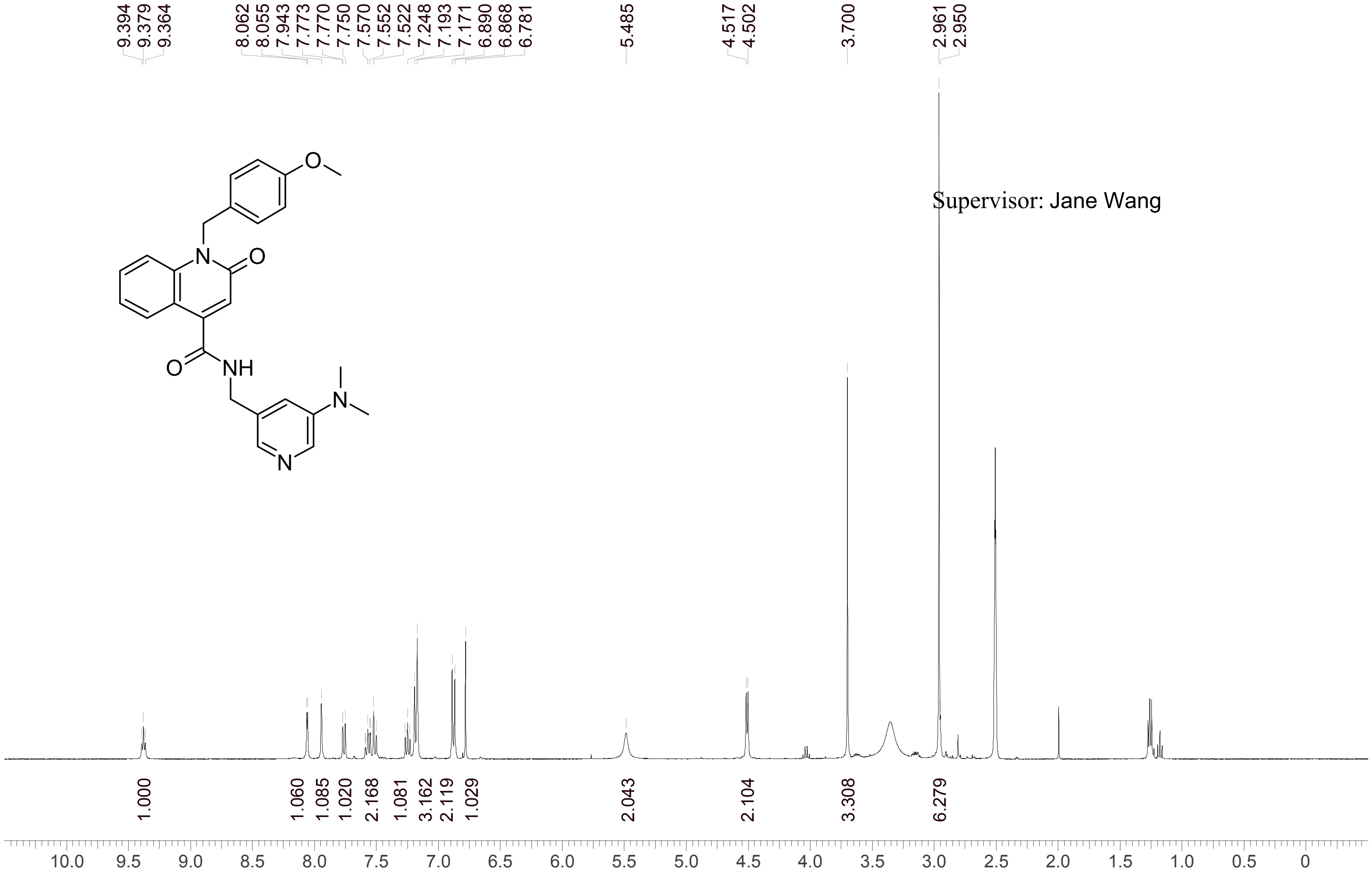

Acquisition Time (sec)	1.9999
Comment	EB2224-187-P1A DMSO Bruker_NT-C_400MHz
Date	23 Oct 2020 09:22:09
Frequency (MHz)	400.1400
Nucleus	1H
Number of Transients	8
Origin	Avance
Original Points Count	16393
Owner	nmrsu
Points Count	65536
Pulse Sequence	zg30
Receiver Gain	101.00
SW(cyclical) (Hz)	8196.72
Solvent	DMSO-d6
Spectrum Offset (Hz)	2400.8411
Spectrum Type	standard
Sweep Width (Hz)	8196.60
Temperature (degree C)	21.981

^{1}H NMR (400MHz, DMSO-d_6) δ = 9.38 (t, *J*=5.9 Hz, 1H), 8.06 (d, *J*=2.8 Hz, 1H), 7.94 (s, 1H), 7.76 (dd, *J*=1.1, 8.1 Hz, 1H), 7.60 - 7.49 (m, 2H), 7.25 (t, *J*=7.4 Hz, 1H), 7.20 - 7.15 (m, 3H), 6.88 (d, *J*=8.8 Hz, 2H), 6.78 (s, 1H), 5.49 (br s, 2H), 4.51 (d, *J*=5.9 Hz, 2H), 3.70 (s, 3H), 2.96 (s, 6H)

Confidential. For research only Not for regulatory filing

Operator:

Date:
