# Supplementary material for: Discovery of SARS-CoV-2 main protease inhibitors using a synthesis-directed de novo design model
Source: Chem Commun (Camb). 2021 May 6;57(48):5909–12. doi: 10.1039/d1cc00050k (PMC8204246; doi:10.1039/d1cc00050k)
Supplement: CC-057-D1CC00050K-s059 [file CC-057-D1CC00050K-s059.pdf]

Compound ID: 00000000

EB2224-191-P1A DMSO Bruker_NT-A_400MHZ

WuXi AppTec
An Integrated R&D Service Company

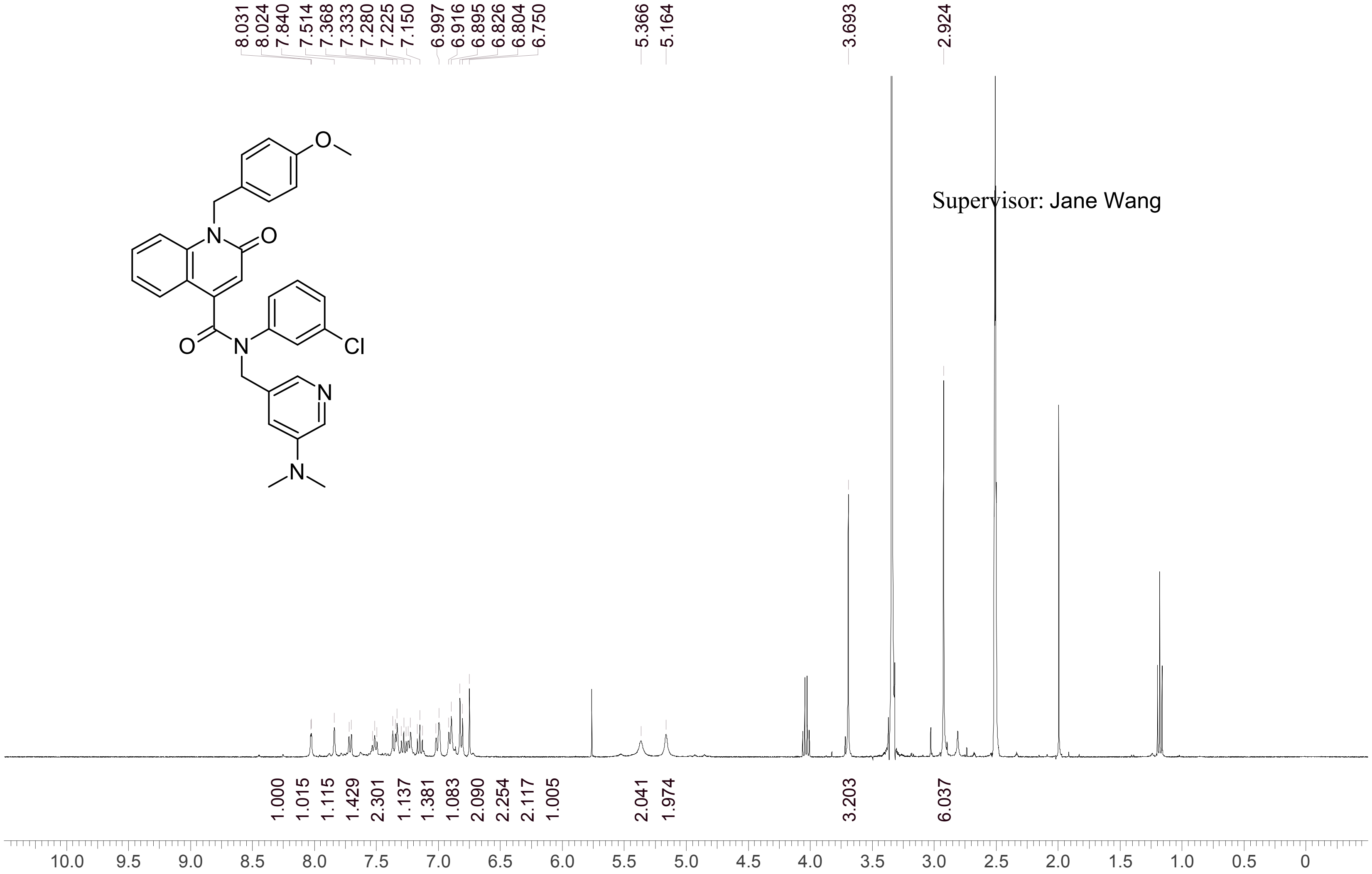

Acquisition Time (sec)	1.9999
Comment	EB2224-191-P1A DMSO Bruker_NT-A_400MHZ
Date	27 Oct 2020 05:06:17
Frequency (MHz)	400.1500
Nucleus	1H
Number of Transients	8
Origin	Avance
Original Points Count	16393
Owner	nmrsu
Points Count	65536
Pulse Sequence	zg30
Receiver Gain	101.00
SW(cyclical) (Hz)	8196.72
Solvent	DMSO-d6
Spectrum Offset (Hz)	2471.2373
Spectrum Type	standard
Sweep Width (Hz)	8196.60
Temperature (degree C)	24.192

^{1}H NMR (400MHz, DMSO-d_6) δ = 8.03 (d, *J*=2.6 Hz, 1H), 7.84 (s, 1H), 7.71 (d, *J*=7.0 Hz, 1H), 7.54 - 7.48 (m, 1H), 7.38 - 7.32 (m, 2H), 7.28 (t, *J*=7.8 Hz, 1H), 7.25 - 7.21 (m, 1H), 7.15 (t, *J*=8.0 Hz, 1H), 7.18 - 7.13 (m, 1H), 7.04 - 6.97 (m, 2H), 6.93 - 6.88 (m, 2H), 6.85 - 6.79 (m, 2H), 6.75 (s, 1H), 5.37 (br s, 2H), 5.16 (br s, 2H), 3.69 (s, 3H), 2.92 (s, 6H)

Confidential. For research only Not for regulatory filing

Operator:

Date:
