# Supplementary material for: Discovery of SARS-CoV-2 main protease inhibitors using a synthesis-directed de novo design model
Source: Chem Commun (Camb). 2021 May 6;57(48):5909–12. doi: 10.1039/d1cc00050k (PMC8204246; doi:10.1039/d1cc00050k)

Confidential. For research information only.

# LCMS REPORT

Print time : 07/21/2020 12:30:46

Compound ID :1
Sample ID : EB2257-9-P1A1
Injection Data : 12:28:24
Injection Vol : 2ul
Location :tray1 vail32
Acq Method :D:\method\10-80AB_2min_220&254_Shimadzu.lcm
Org DataFile :D:\DATA\2020\2007\200721\EB2257-9-P1A1.lcd
Instrument & column:LCMS-SAP 1-2402
Xtimate C18, 3um,2.1*30mm

Chromatogram

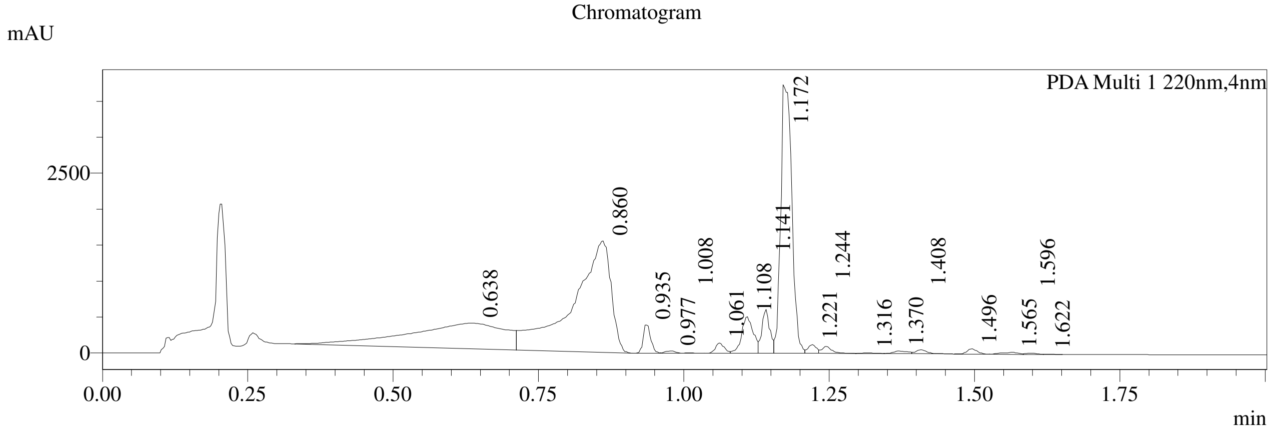


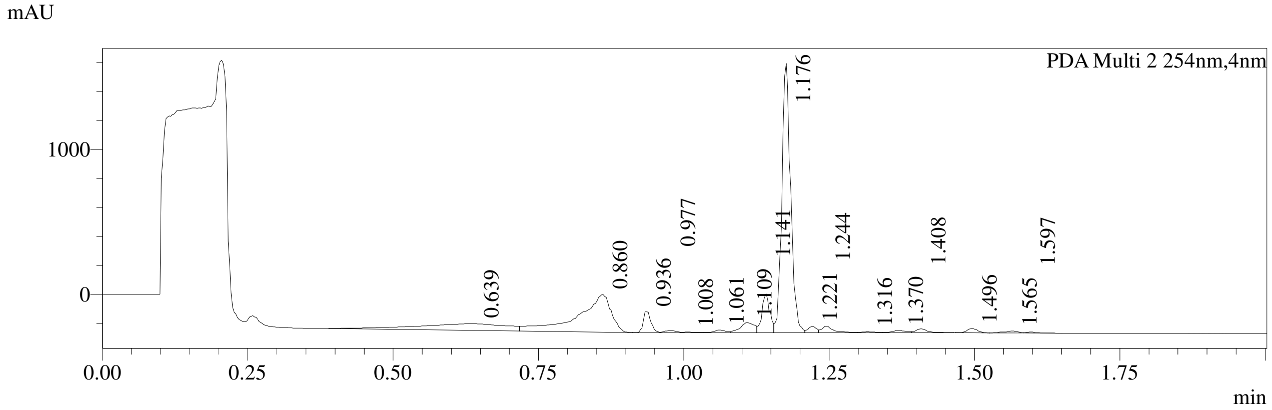


1 PDA Multi 1 / 220nm,4nm
2 PDA Multi 2 / 254nm,4nm

MS Chromatogram

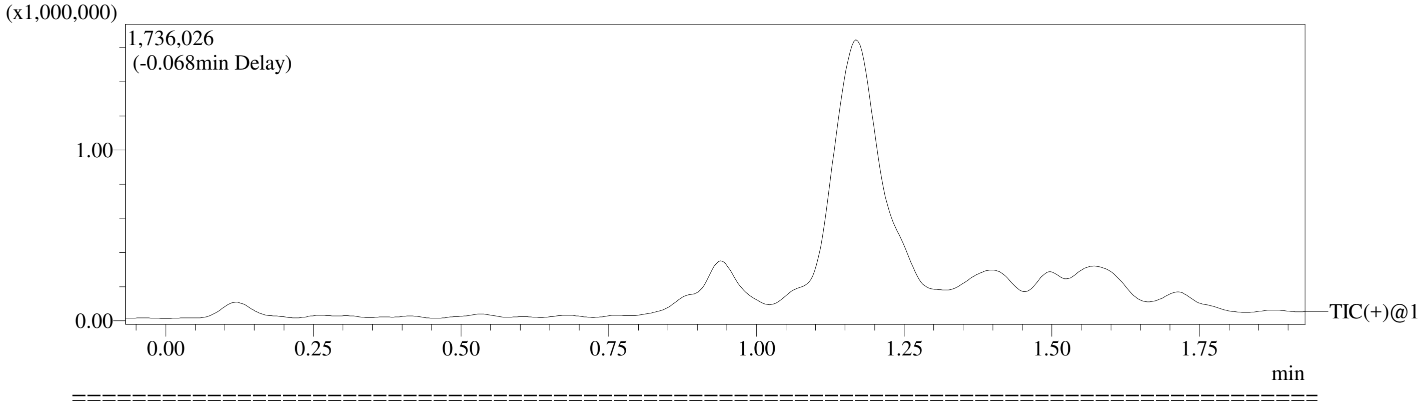


## Integration Result

AD2

PDA Ch1 220nm

| Peak# | Ret. Time | Height | Height% | USP Width | Area | Area% |
|---|---|---|---|---|---|---|
| 1 | 0.638 | 355545 | 4.572 | 0.418 | 4099902 | 22.137 |
| 2 | 0.860 | 1548819 | 19.917 | 0.109 | 7060834 | 38.125 |
| 3 | 0.935 | 392469 | 5.047 | 0.026 | 354161 | 1.912 |
| 4 | 0.977 | 30171 | 0.388 | 0.036 | 38843 | 0.210 |
| 5 | 1.008 | 6277 | 0.081 | 0.037 | 7378 | 0.040 |
| 6 | 1.061 | 143086 | 1.840 | 0.033 | 156259 | 0.844 |
| 7 | 1.108 | 509594 | 6.553 | 0.044 | 720276 | 3.889 |
| 8 | 1.141 | 613605 | 7.891 | 0.027 | 581743 | 3.141 |
| 9 | 1.172 | 3733111 | 48.006 | 0.033 | 4950343 | 26.729 |
| 10 | 1.221 | 123328 | 1.586 | 0.037 | 120882 | 0.653 |
| 11 | 1.244 | 95042 | 1.222 | 0.038 | 112421 | 0.607 |
| 12 | 1.316 | 7522 | 0.097 | 0.037 | 8754 | 0.047 |
| 13 | 1.370 | 34375 | 0.442 | 0.057 | 58736 | 0.317 |
| 14 | 1.408 | 57640 | 0.741 | 0.041 | 75330 | 0.407 |
| 15 | 1.496 | 73402 | 0.944 | 0.037 | 89965 | 0.486 |
| 16 | 1.565 | 28834 | 0.371 | 0.059 | 56329 | 0.304 |
| 17 | 1.596 | 17441 | 0.224 | 0.042 | 22731 | 0.123 |
| 18 | 1.622 | 6028 | 0.078 | 0.043 | 5428 | 0.029 |

PDA Ch2 254nm

| Peak# | Ret. Time | Height | Height% | USP Width | Area | Area% |
|---|---|---|---|---|---|---|
| 1 | 0.639 | 46291 | 1.607 | 0.362 | 484095 | 11.300 |
| 2 | 0.860 | 260057 | 9.028 | 0.082 | 1028018 | 23.996 |
| 3 | 0.936 | 144706 | 5.023 | 0.026 | 133420 | 3.114 |
| 4 | 0.977 | 13955 | 0.484 | 0.039 | 19229 | 0.449 |
| 5 | 1.008 | 4399 | 0.153 | 0.061 | 7546 | 0.176 |
| 6 | 1.061 | 19200 | 0.667 | 0.040 | 25384 | 0.593 |
| 7 | 1.109 | 70596 | 2.451 | 0.056 | 118748 | 2.772 |
| 8 | 1.141 | 270126 | 9.377 | 0.028 | 252414 | 5.892 |
| 9 | 1.176 | 1856779 | 64.456 | 0.031 | 1955518 | 45.646 |
| 10 | 1.221 | 44691 | 1.551 | 0.038 | 44438 | 1.037 |
| 11 | 1.244 | 45836 | 1.591 | 0.038 | 63885 | 1.491 |
| 12 | 1.316 | 6651 | 0.231 | 0.069 | 12067 | 0.282 |
| 13 | 1.370 | 17310 | 0.601 | 0.044 | 29659 | 0.692 |
| 14 | 1.408 | 27890 | 0.968 | 0.040 | 37785 | 0.882 |
| 15 | 1.496 | 31197 | 1.083 | 0.037 | 37727 | 0.881 |
| 16 | 1.565 | 13154 | 0.457 | 0.046 | 22290 | 0.520 |
| 17 | 1.597 | 7865 | 0.273 | 0.038 | 11851 | 0.277 |

Operator:______________

Date:_________________

Mass Spectrum

RetTime: 0.638  Datafile: D:\DATA\2020\2007\200721\EB2257-9-P1A1.lcd

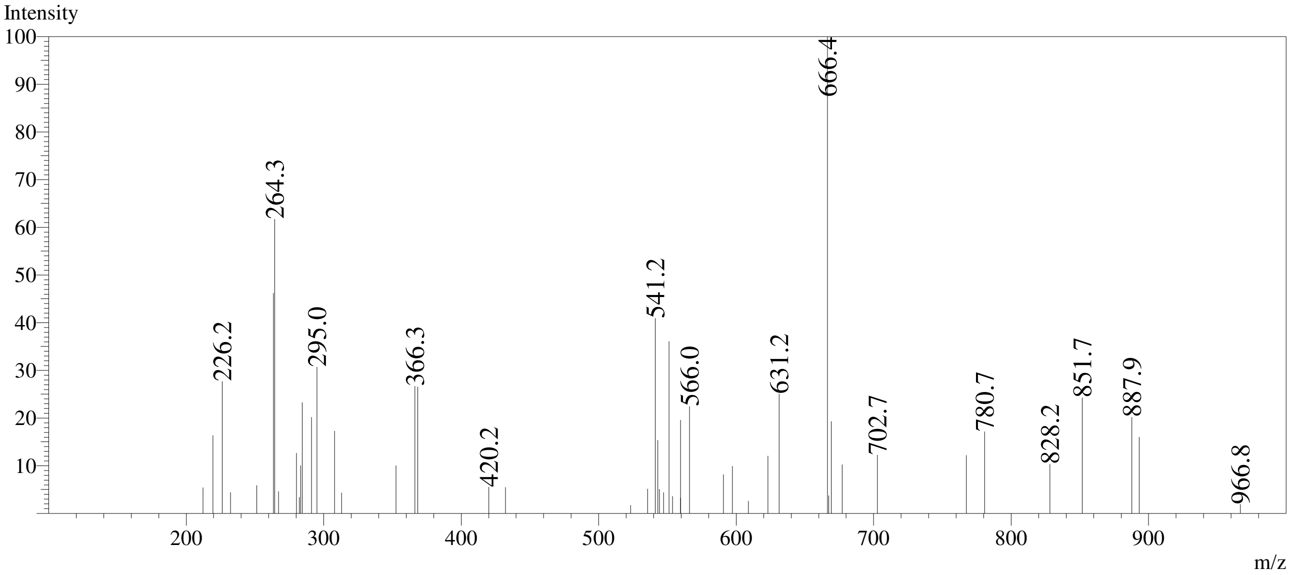


RetTime: 0.862  Datafile: D:\DATA\2020\2007\200721\EB2257-9-P1A1.lcd

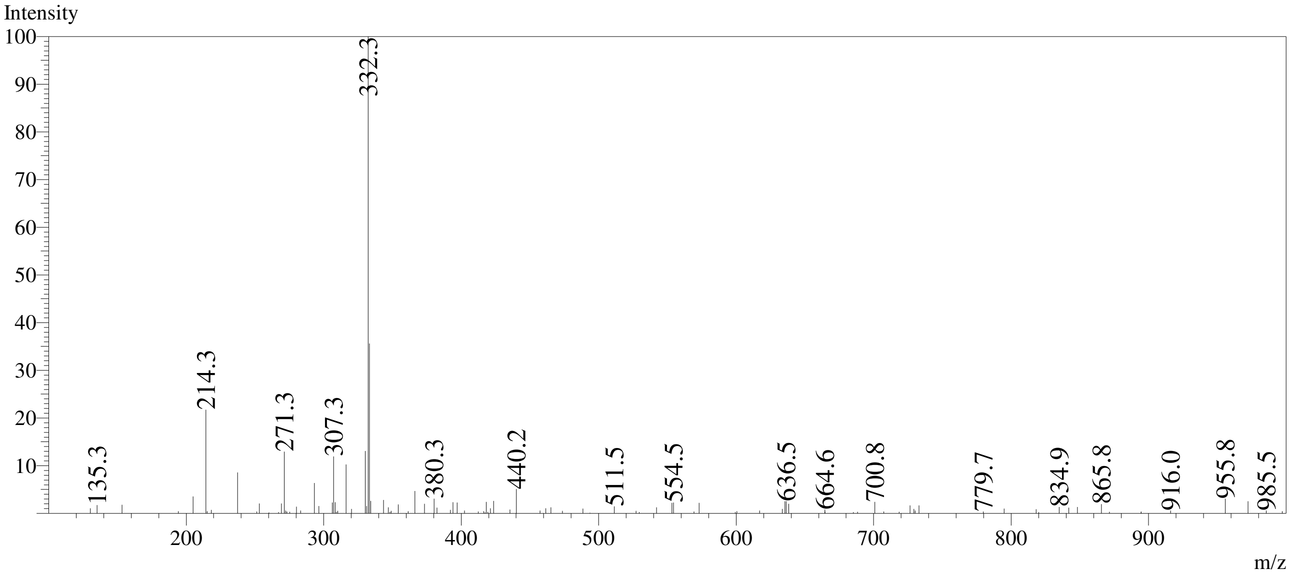


RetTime: 1.108  Datafile: D:\DATA\2020\2007\200721\EB2257-9-P1A1.lcd

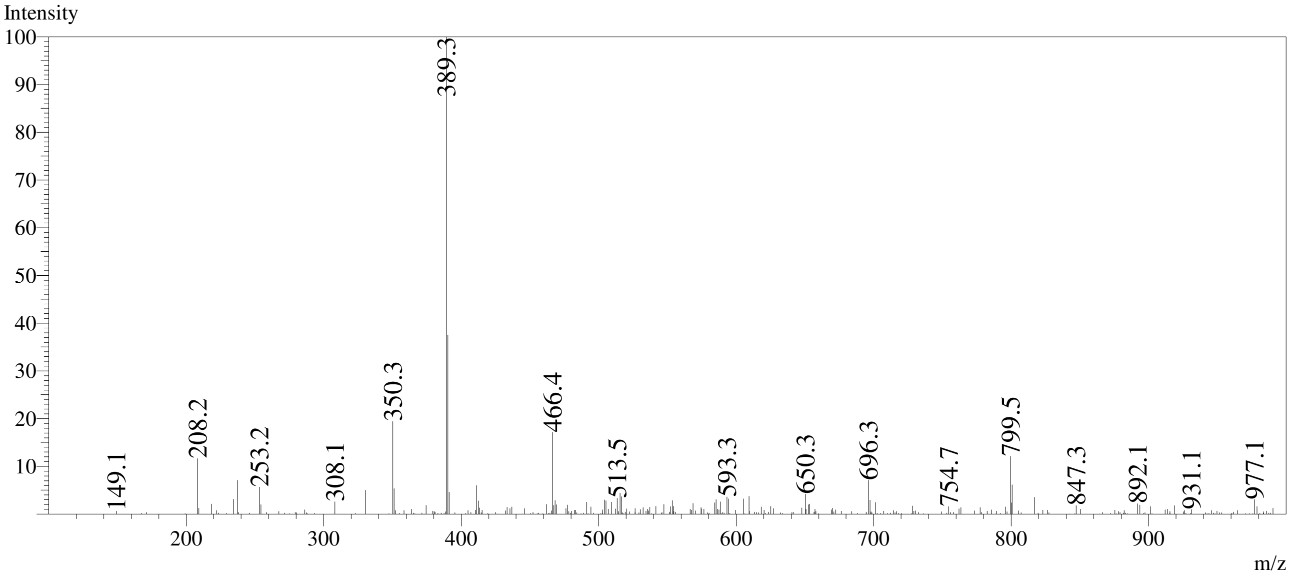

RetTime: 1.142  Datafile: D:\DATA\2020\2007\200721\EB2257-9-P1A1.lcd

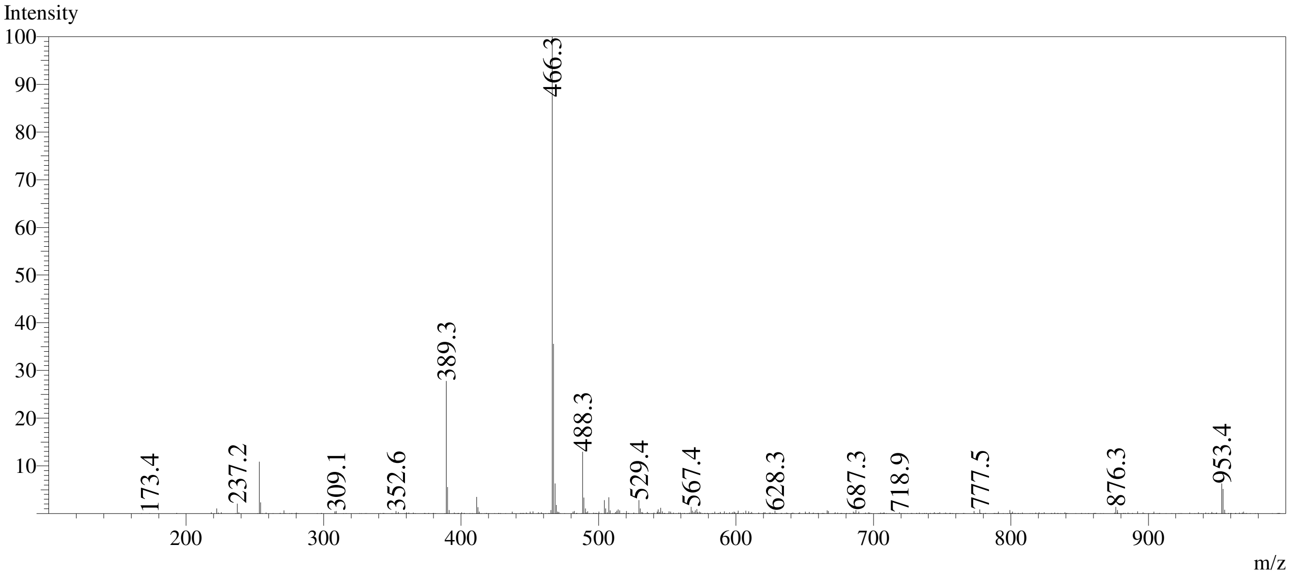


RetTime: 1.172  Datafile: D:\DATA\2020\2007\200721\EB2257-9-P1A1.lcd

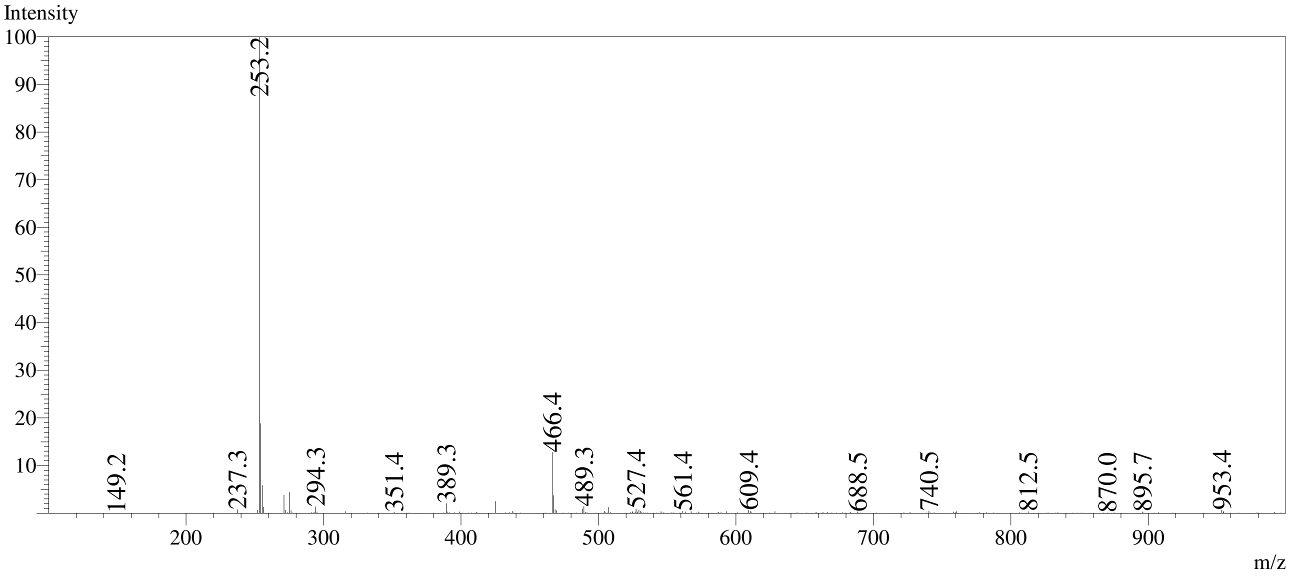

Supplement: CC-057-D1CC00050K-s063 [file CC-057-D1CC00050K-s063.pdf]
