# Supplementary material for: Discovery of SARS-CoV-2 main protease inhibitors using a synthesis-directed de novo design model
Source: Chem Commun (Camb). 2021 May 6;57(48):5909–12. doi: 10.1039/d1cc00050k (PMC8204246; doi:10.1039/d1cc00050k)
Supplement: CC-057-D1CC00050K-s067 [file CC-057-D1CC00050K-s067.pdf]

Compound ID: 00000000

EB2257-12-P1A MeOD Bruker_NT-A_400MHZ

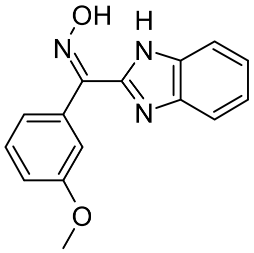

Supervisor: Jane Wang

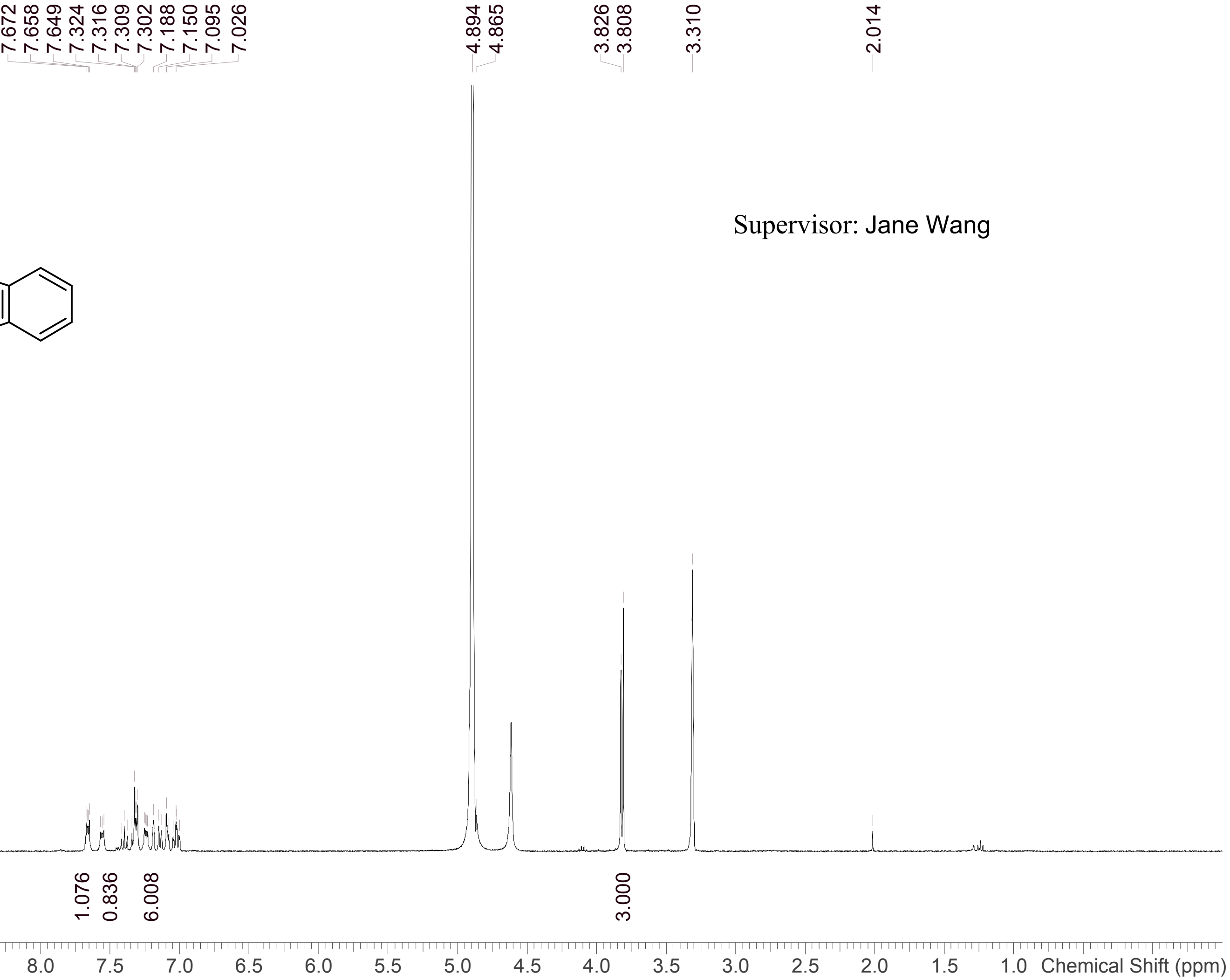

Acquisition Time (sec)	1.9999
Comment	EB2257-12-P1A MeOD Bruker_NT-A_400MHZ
Date	23 Jul 2020 05:11:32
Frequency (MHz)	400.1500
Nucleus	1H
Number of Transients	8
Origin	Avance
Original Points Count	16393
Owner	nmrsu
Points Count	65536
Pulse Sequence	zg30
Receiver Gain	101.00
SW(cyclical) (Hz)	8196.72
Solvent	METHANOL-d4
Spectrum Offset (Hz)	2463.7144
Spectrum Type	standard
Sweep Width (Hz)	8196.60
Temperature (degree C)	-273.000

^{1}H NMR (400MHz, METHANOL-d_4) δ = 7.71 - 7.62 (m, 1H), 7.60 - 7.51 (m, 1H), 7.44 - 6.98 (m, 6H), 3.82 (d, *J*=7.3 Hz, 3H)

Confidential. For research only Not for regulatory filing

Operator:

Date:
