# Supplementary material for: Discovery of SARS-CoV-2 main protease inhibitors using a synthesis-directed de novo design model
Source: Chem Commun (Camb). 2021 May 6;57(48):5909–12. doi: 10.1039/d1cc00050k (PMC8204246; doi:10.1039/d1cc00050k)

Confidential. For research information only.

LCMS REPORT

Print time : 07/30/2020 14:22:01
Compound ID :1
Sample ID : EB2257-29-P1A
Injection Date :2020/7/30 14:17:50
Injection Vol : 2ul
Location :tray1 vail61
Acq Method :10-80AB_4min_220&254_Shimadzu.lcm
Org Data File :D:\DATA\2020\2007\200730\EB2257-29-P1A.lcd
Instrument & column:LCMS_04 1-2402
Xtimate C18, 3um,2.1*30mm

Chromatogram

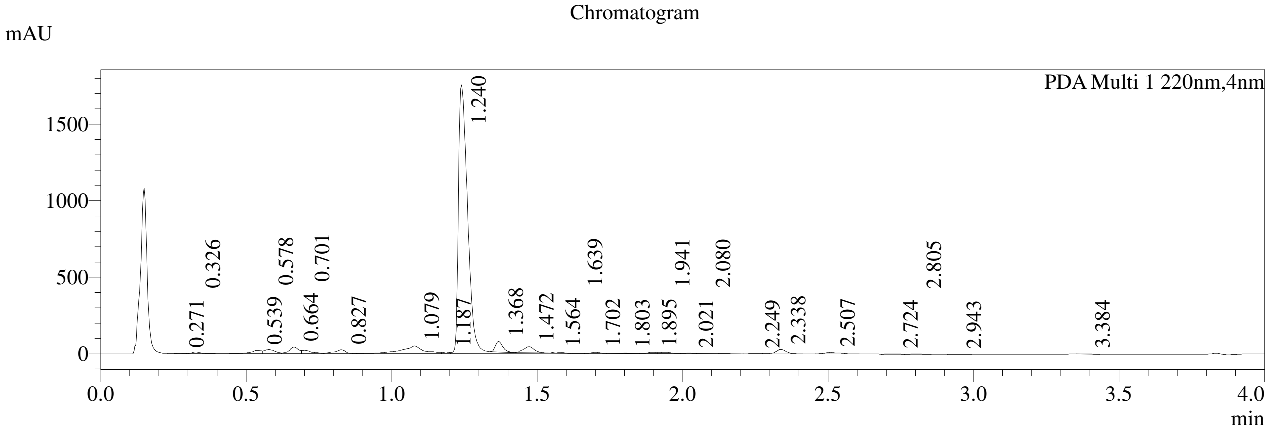

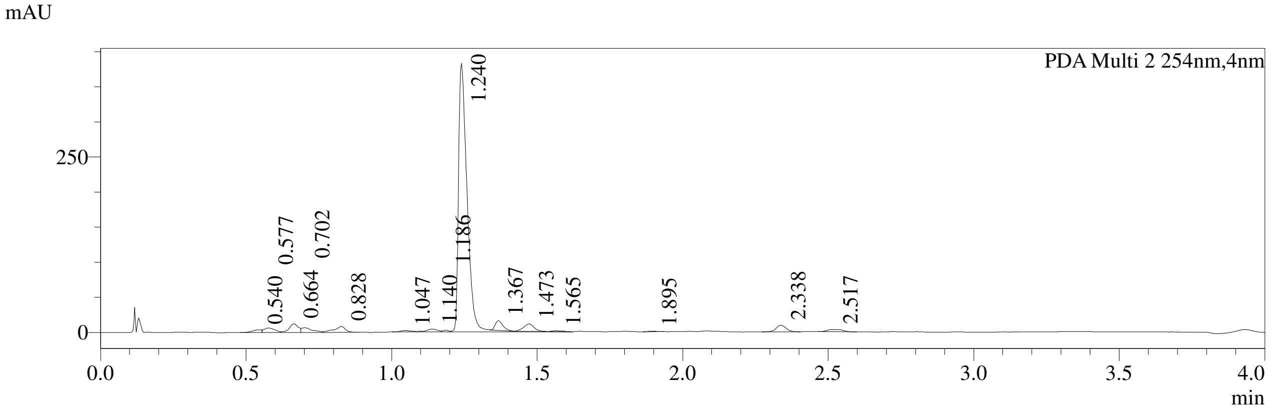

1 PDA Multi 1 / 220nm,4nm
2 PDA Multi 2 / 254nm,4nm

MS Chromatogram

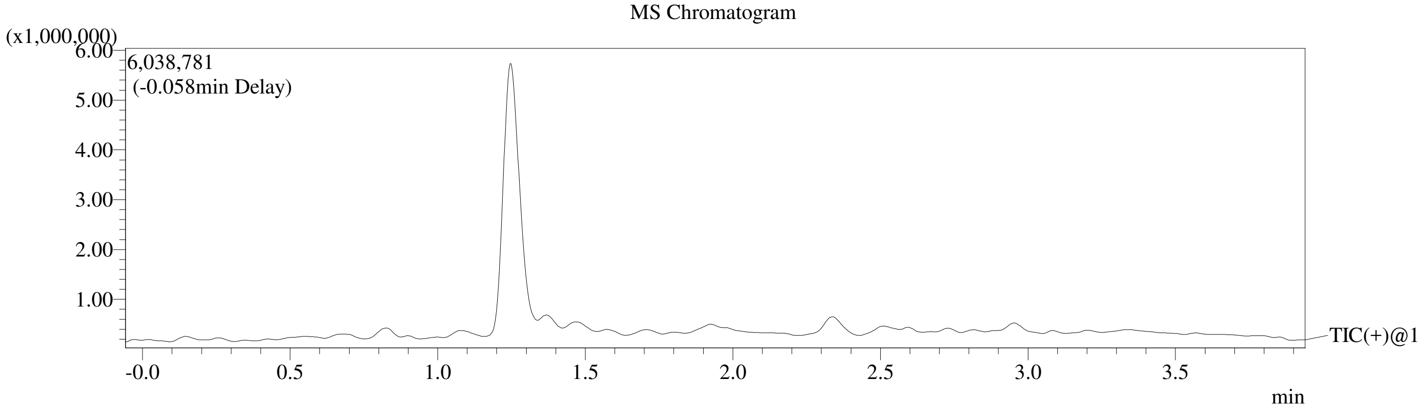

==

Integration Result

==

PDA Ch1 220nm

Peak#	Ret. Time	Height	Height%	USP Width	Area	Area%
1	0.271	1035	0.048	0.038	1337	0.026
2	0.326	10115	0.466	0.055	19562	0.380
3	0.539	22270	1.026	0.104	55335	1.075
4	0.578	27132	1.250	0.099	71007	1.379
5	0.664	44095	2.032	0.069	99958	1.942
6	0.701	22481	1.036	0.170	51743	1.005
7	0.827	26026	1.199	0.066	70852	1.376
8	1.079	50984	2.349	0.103	281037	5.460
9	1.187	10737	0.495	0.086	17253	0.335
10	1.240	1756406	80.929	0.061	4020229	78.098
11	1.368	69796	3.216	0.050	122280	2.375
12	1.472	39711	1.830	0.074	103067	2.002
13	1.564	6995	0.322	0.069	16410	0.319
14	1.639	1182	0.054	0.046	1686	0.033
15	1.702	7088	0.327	0.060	14800	0.288
16	1.803	2614	0.120	0.093	9746	0.189
17	1.895	8507	0.392	0.092	18866	0.366
18	1.941	9273	0.427	0.098	26300	0.511
19	2.021	4654	0.214	0.081	10883	0.211
20	2.080	4009	0.185	0.129	17050	0.331
21	2.249	2252	0.104	0.075	5202	0.101
22	2.338	29222	1.346	0.066	71450	1.388
23	2.507	9291	0.428	0.099	32014	0.622
24	2.724	1029	0.047	0.067	2501	0.049
25	2.805	1584	0.073	0.055	3263	0.063
26	2.943	774	0.036	0.060	1669	0.032
27	3.384	1043	0.048	0.061	2165	0.042

PDA Ch2 254nm

Peak#	Ret. Time	Height	Height%	USP Width	Area	Area%
1	0.540	3877	0.828	0.157	8595	0.863
2	0.577	6427	1.373	0.089	16255	1.632
3	0.664	12397	2.648	0.069	26996	2.710
4	0.702	6673	1.425	0.172	16639	1.670
5	0.828	8425	1.799	0.064	22485	2.257
6	1.047	2173	0.464	0.076	5731	0.575
7	1.140	4019	0.858	0.075	10899	1.094
8	1.186	2214	0.473	0.061	3198	0.321
9	1.240	382641	81.719	0.057	793955	79.695
10	1.367	13895	2.968	0.052	24899	2.499
11	1.473	10461	2.234	0.070	25946	2.604
12	1.565	1331	0.284	0.070	3439	0.345
13	1.895	920	0.196	0.059	1915	0.192
14	2.338	9504	2.030	0.065	22989	2.308
15	2.517	3281	0.701	0.092	12296	1.234

Operator:______________

Date:_________________

Mass Spectrum

RetTime: 1.078 Datafile: D:\DATA\2020\2007\200730\EB2257-29-P1A.lcd

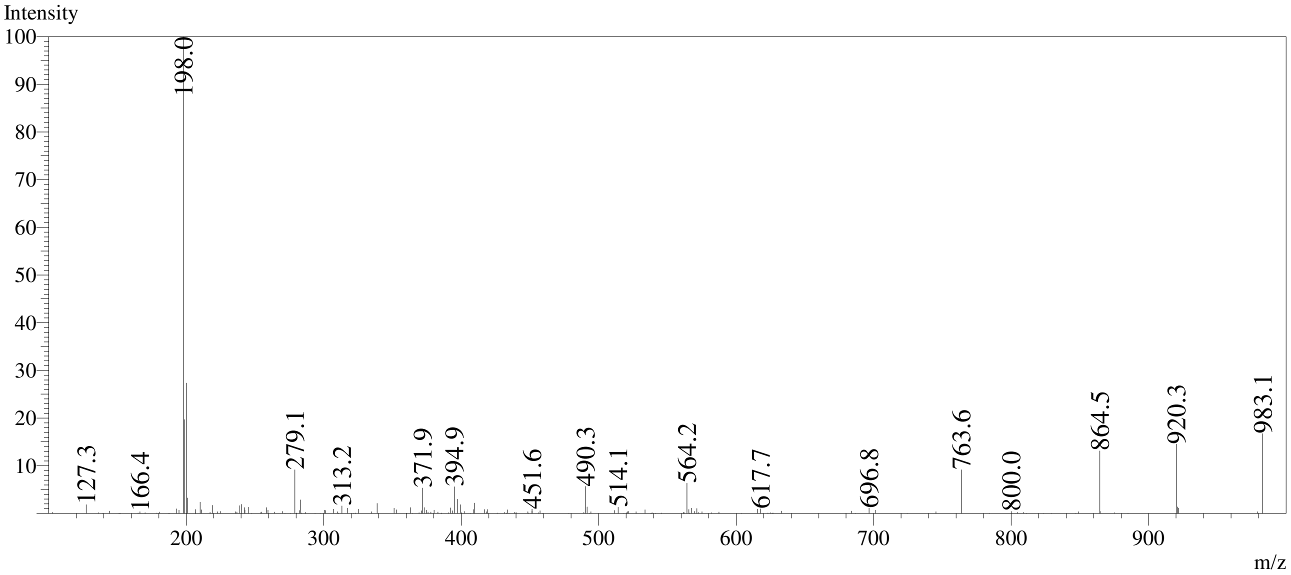

RetTime: 1.238 Datafile: D:\DATA\2020\2007\200730\EB2257-29-P1A.lcd

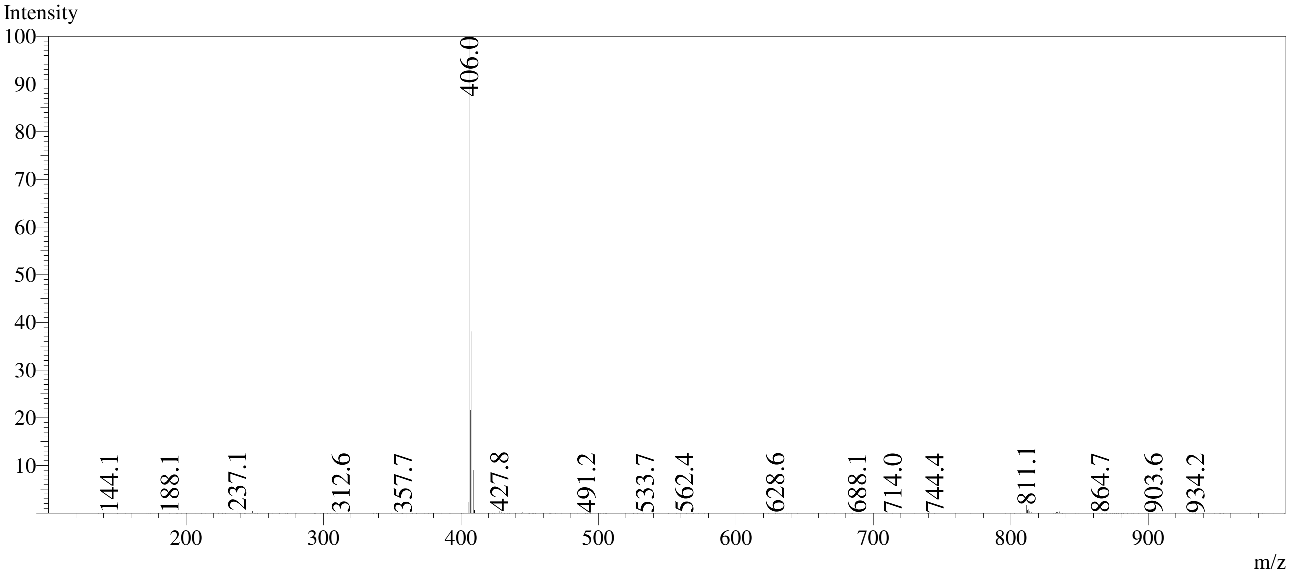

RetTime: 1.368 Datafile: D:\DATA\2020\2007\200730\EB2257-29-P1A.lcd

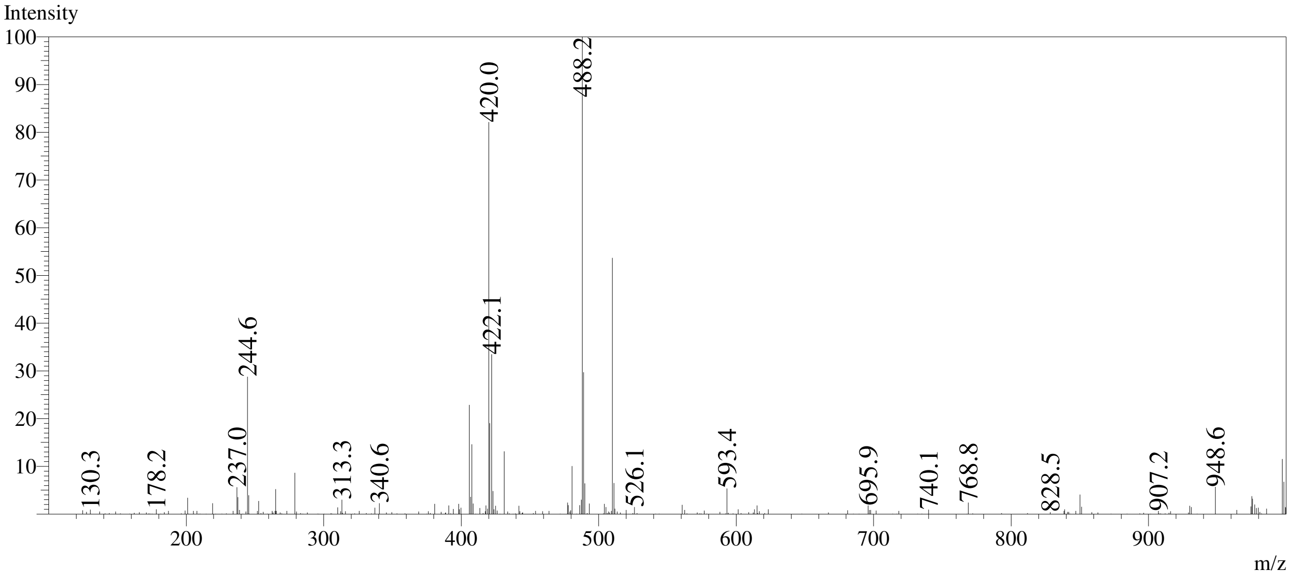

RetTime: 1.472 Datafile: D:\DATA\2020\2007\200730\EB2257-29-P1A.lcd

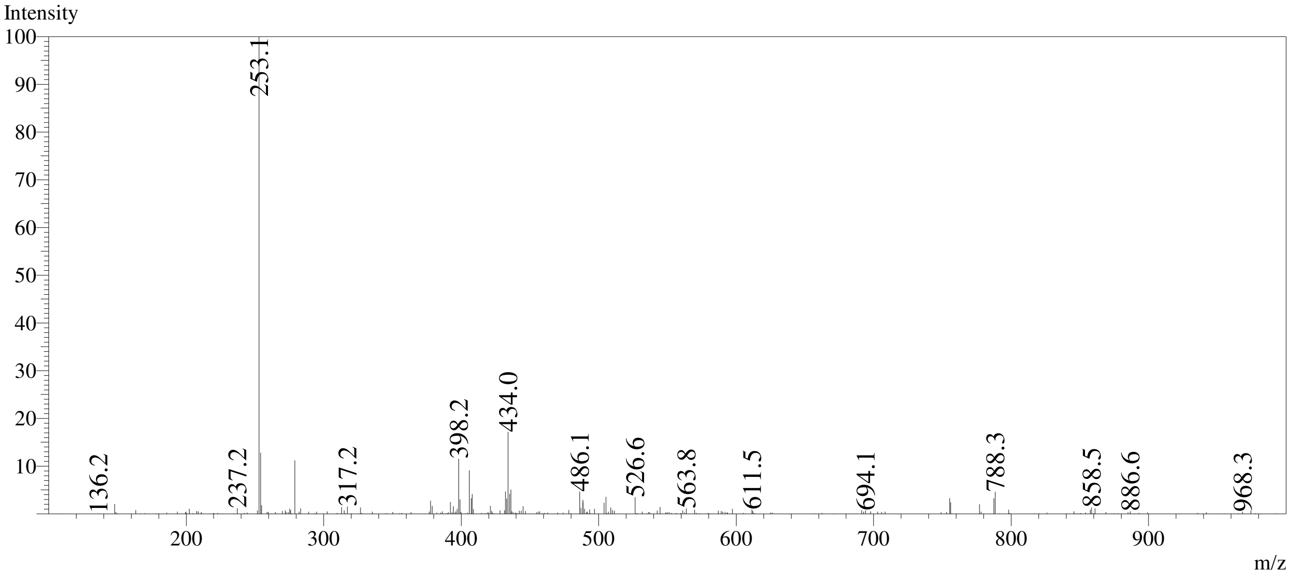

Supplement: CC-057-D1CC00050K-s069 [file CC-057-D1CC00050K-s069.pdf]
