# Supplementary material for: Discovery of SARS-CoV-2 main protease inhibitors using a synthesis-directed de novo design model
Source: Chem Commun (Camb). 2021 May 6;57(48):5909–12. doi: 10.1039/d1cc00050k (PMC8204246; doi:10.1039/d1cc00050k)

Compound ID: 00000000

EB2257-29-P1B CDCl3 Bruker_NT-C_400MHz

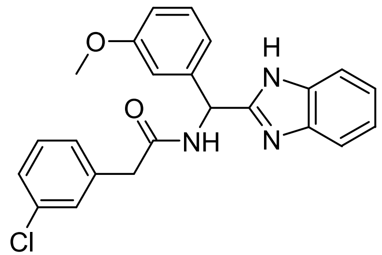

Chemical Formula: $C_{23}H_{20}ClN_3O_2$

Molecular Weight: 405.88

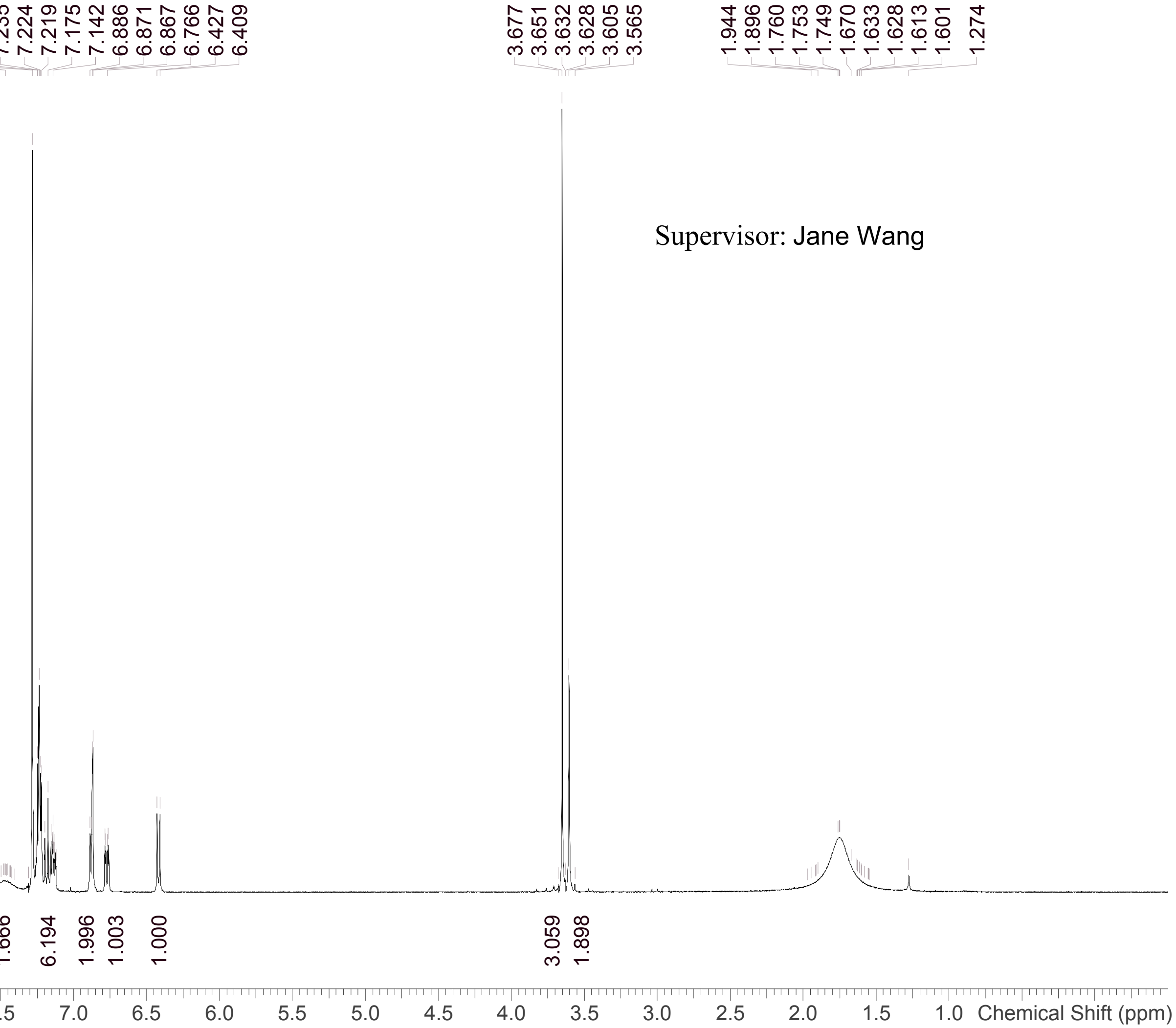

Acquisition Time (sec)	1.9999
Comment	EB2257-29-P1B CDCl3 Bruker_NT-C_400MHz
Date	03 Aug 2020 03:43:53
Frequency (MHz)	400.1400
Nucleus	1H
Number of Transients	8
Origin	Avance
Original Points Count	16393
Owner	nmrsu
Points Count	65536
Pulse Sequence	zg30
Receiver Gain	101.00
SW(cyclical) (Hz)	8196.72
Solvent	CHLOROFORM-d
Spectrum Offset (Hz)	2400.8411
Spectrum Type	standard
Sweep Width (Hz)	8196.60
Temperature (degree C)	22.926

^{1}H NMR (400MHz, CHLOROFORM-d) δ = 7.87 (br d, *J*=7.5 Hz, 1H), 7.65 - 7.34 (m, 2H), 7.27 - 7.09 (m, 6H), 6.91 - 6.84 (m, 2H), 6.82 - 6.71 (m, 1H), 6.42 (d, *J*=7.5 Hz, 1H), 3.67 - 3.63 (m, 3H), 3.60 (s, 2H)

Confidential. For research only Not for regulatory filing

Operator:

Date:

Confidential. For research information only.

LCMS REPORT

Print time	: 07/31/2020 10:38:12
Compound ID	: 1
Sample ID	: EB2257-29-P1J
Injection Date	:7/31/2020 10:34:02 AM
Injection Vol	: 2ul
Location	:tray1 vail42
Acq Method	:10-80AB_4min_220&254_Shimadzu.lcm
Org Data File	:D:\DATA\2020\2007\200731\EB2257-29-P1J.lcd
Instrument	:LCMS_01 1-2402 Shimadzu LCMS-2020
Column	:Xtimate C18,3um,2.1*30mm
Mobile phase	:A) 0.04% TFA in H2O B) 0.02% TFA in ACN
Gradient	:10% B increase to 80% B within 3min; hold at 80% B for 0.5min; then back to 10% B at 3.51min and hold for 0.49min. Flow rate 1.0 mL/min

Chromatogram

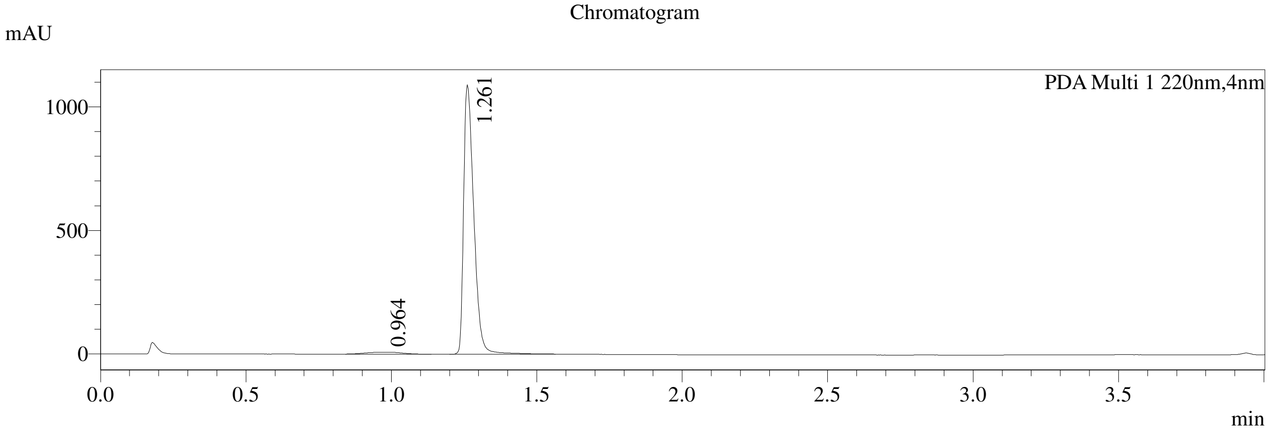

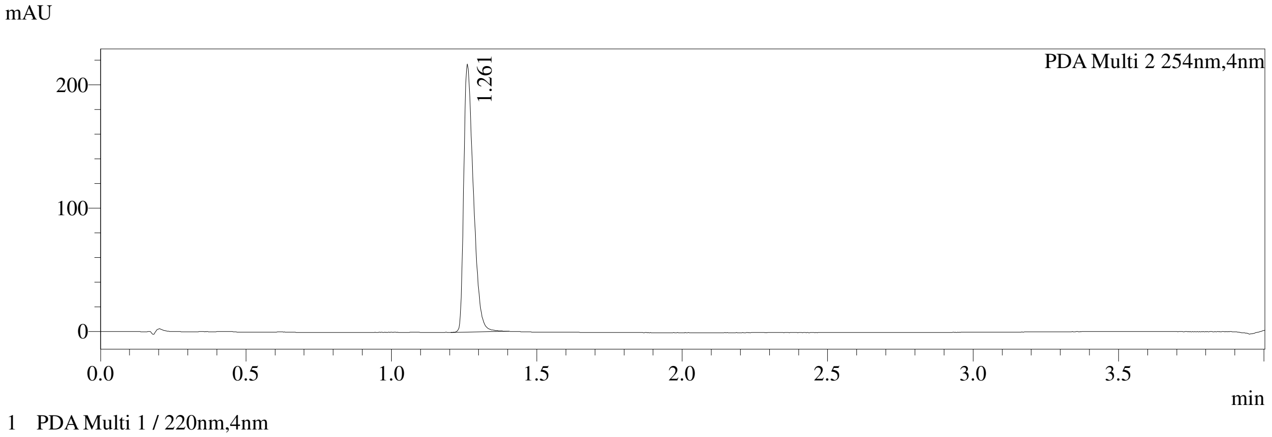

1 PDA Multi 1 / 220nm,4nm
2 PDA Multi 2 / 254nm,4nm

MS Chromatogram

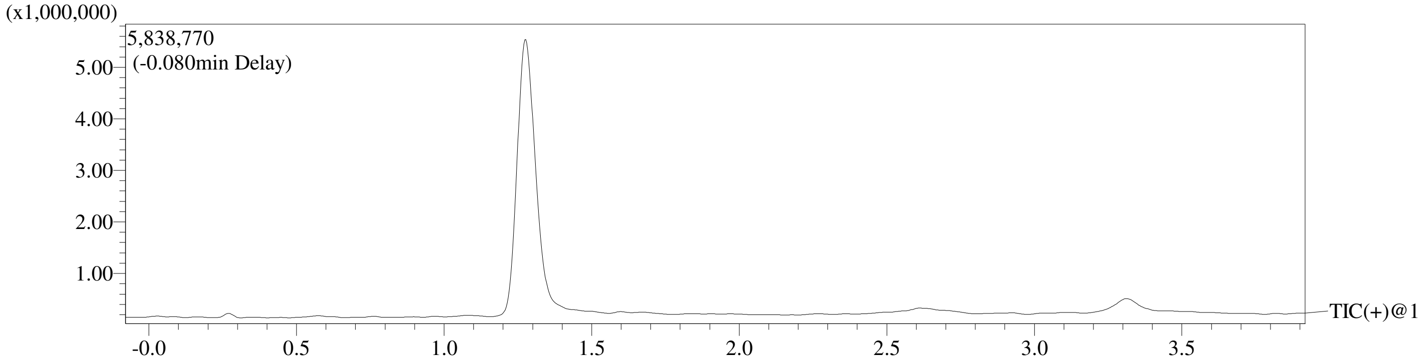

==

Integration Result

==

Peak Table

AD2

PDA Ch1 220nm

Peak#	Ret. Time	Height	Height%	USP Width	Area	Area%
1	0.964	7555	0.688	0.215	67159	2.475
2	1.261	1090429	99.312	0.066	2646582	97.525

PDA Ch2 254nm

Peak#	Ret. Time	Height	Height%	USP Width	Area	Area%
1	1.261	217177	100.000	0.064	500360	100.000

Operator:______________

Date:__________________

Mass Spectrum

RetTime: 0.963 Datafile: D:\DATA\2020\2007\200731\EB2257-29-P1J.lcd

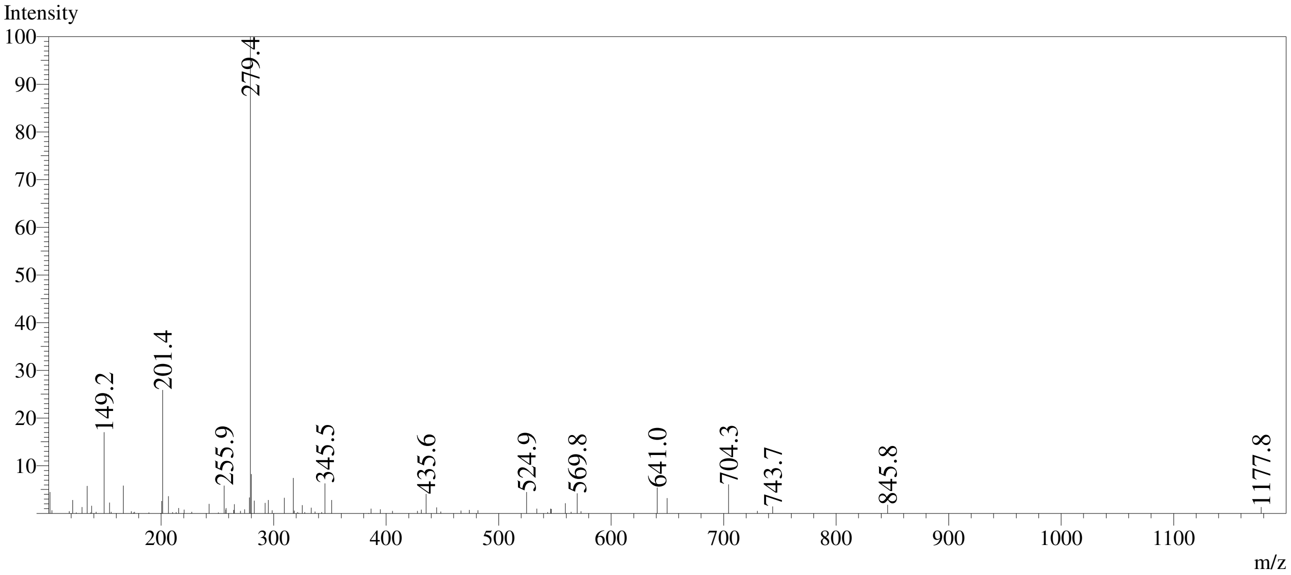

RetTime: 1.260 Datafile: D:\DATA\2020\2007\200731\EB2257-29-P1J.lcd

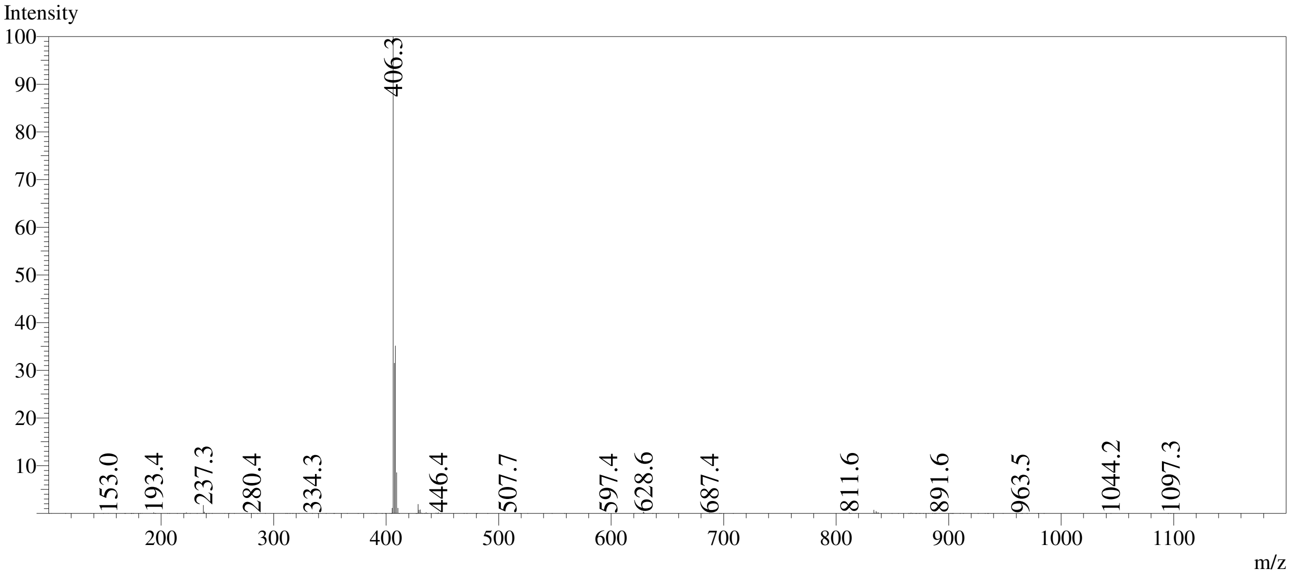

Supplement: CC-057-D1CC00050K-s071 [file CC-057-D1CC00050K-s071.pdf]
