# Supplementary material for: Discovery of SARS-CoV-2 main protease inhibitors using a synthesis-directed de novo design model
Source: Chem Commun (Camb). 2021 May 6;57(48):5909–12. doi: 10.1039/d1cc00050k (PMC8204246; doi:10.1039/d1cc00050k)
Supplement: CC-057-D1CC00050K-s073 [file CC-057-D1CC00050K-s073.pdf]

Compound ID: 00000000

EB2257-9-P1A CDCl3 Bruker_NT-C_400MHz

WuXi AppTec
An Integrated R&D Service Company

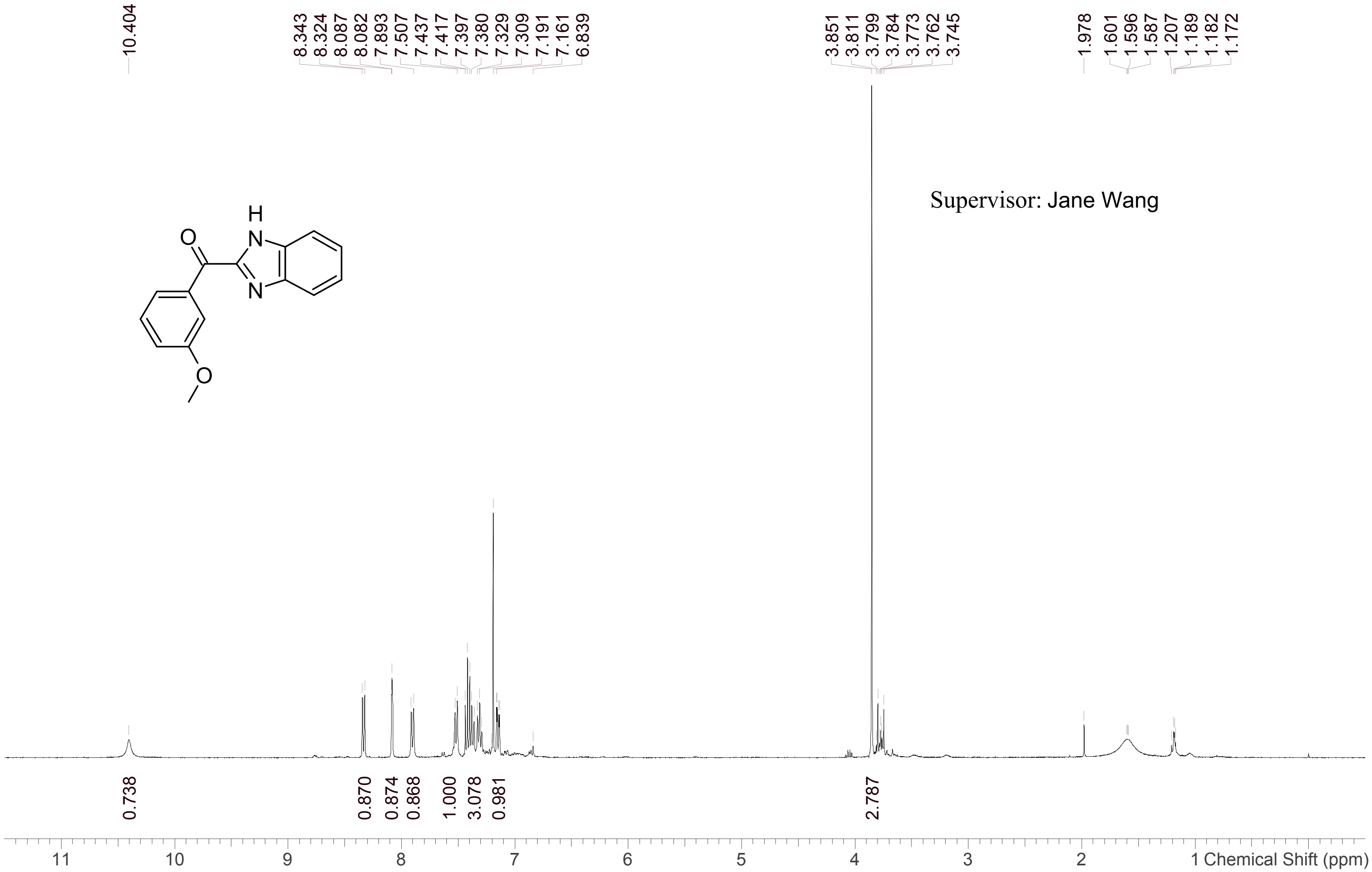

Supervisor: Jane Wang

Acquisition Time (sec)	1.9999
Comment	EB2257-9-P1A CDCl3 Bruker_NT-C_400MHz
Date	21 Jul 2020 10:19:40
Frequency (MHz)	400.1400
Nucleus	1H
Number of Transients	8
Origin	Avance
Original Points Count	16393
Owner	nmrsu
Points Count	65536
Pulse Sequence	zg30
Receiver Gain	101.00
SW(cyclical) (Hz)	8196.72
Solvent	CHLOROFORM-d
Spectrum Offset (Hz)	2363.5244
Spectrum Type	standard
Sweep Width (Hz)	8196.60
Temperature (degree C)	22.783

^{1}H NMR (400MHz, CHLOROFORM-d) δ = 10.40 (br s, 1H), 8.33 (d, *J*=7.8 Hz, 1H), 8.08 (d, *J*=1.9 Hz, 1H), 7.90 (d, *J*=8.1 Hz, 1H), 7.55 - 7.49 (m, 1H), 7.44 - 7.28 (m, 3H), 7.15 (dd, *J*=2.6, 8.3 Hz, 1H), 3.85 (s, 3H)

Confidential. For research only Not for regulatory filing

Operator:

Date:
