# Supplementary material for: Discovery of SARS-CoV-2 main protease inhibitors using a synthesis-directed de novo design model
Source: Chem Commun (Camb). 2021 May 6;57(48):5909–12. doi: 10.1039/d1cc00050k (PMC8204246; doi:10.1039/d1cc00050k)
Supplement: CC-057-D1CC00050K-s079 [file CC-057-D1CC00050K-s079.pdf]

Electronic Supplementary Material: Discovery of SARS-CoV-2 main protease inhibitors using a synthesis-directed *de novo* design model

Aaron Morris,[1,*] William McCorkindale,[2,*] The COVID Moonshot Consortium,[3] Nir Drayman,[4] John D. Chodera,[5] Savaş Tay,[4] Nir London,[6] and Alpha A. Lee[1,†]

[1]*PostEra Inc, 2 Embarcadero Centre, San Franciso, CA 94111, United States of America*

[2]*Department of Physics, University of Cambridge, CB3 0HE, United Kingdom*

[3]*The COVID Moonshot Consortium,* `www.postera.ai/covid`

[4]*The Pritzker School for Molecular Engineering, The University of Chicago, Chicago, IL, USA*

[5]*Computational and Systems Biology Program Sloan Kettering Institute, Memorial Sloan Kettering Cancer Center, New York, NY 10065, USA*

[6]*Department of Organic Chemistry, The Weizmann Institute of Science, 76100, Rehovot, Israel*

[*] AM and WJM contributed equally.

[†] alpha.lee@postera.ai

I. FLUORESCENCE MPRO INHIBITION ASSAY

Compounds were seeded into assay-ready plates (Greiner 384 low volume 784900) using an Echo 555 acoustic dispenser, and DMSO was back-filled for a uniform concentration in assay plates (maximum 1%). Screening assays were performed in duplicate at 20 μM and 50 μM. Hits of greater than 50 % inhibition at 50 μM were confirmed by dose response assays. Reagents for Mpro assay reagents were dispensed into the assay plate in 10 µl volumes for a final volume of 20 μL. Final reaction concentrations were 20 mM HEPES pH 7.3, 1 mM TCEP, 50 mM NaCl, 0.01% Tween-20, 10% glycerol, 5nM Mpro, 375nM fluorogenic peptide substrate ([5-FAM]-AVLQSGFR-[Lys(Dabcyl)]-K-amide). Mpro was pre-incubated for 15 minutes at room temperature with compound before addition of substrate. Protease reaction was measured continuously in a BMG Pherastar FS with a 480/520 ex/em filter set. Data analysis was performed with Collaborative Drug Discovery (CDD).

II. OC43 ANTIVIRAL ASSAY

A549 expressing H2B-mRuby were seeded in 384 well plates (4,000 cells per well) in DMEM+2% FCS in a total volume of 30ul. One day later, 20ul of OC43 were added to the wells for a final MOI of 0.3. one hour after viral addition, the drug (or DMSO as control) was added to the cells. Drugs were added at a volume of 50nl, in a final dose range of 0.3-20mM. Cells were incubated at 33C, 5% CO2 for 2 days, fixed with paraformaldehyde and stained for the presence of the viral nucleoprotein. Images were captured and quantified using the Incucyte machine and software. 3 biological repeated were performed.

III. COMPOUND SYNTHESIS

Compounds 1-5 were sourced from Wuxi AppTec and used as received. Synthesis routes reported by Wuxi AppTec is appended in the ESI.

IV. LEARNING-TO-RANK

Our learning-to-rank methods converts ranking into binary classification of whether a compound is more/less active than another compound. This allows us to assimilate both

coarse (active/inactive) and fine (quantitative potency measurements) into a single model. All inactive compounds are less active than active compounds, and compounds with potency measurements are ranked by their potency.

To represent a molecule, we concatenate 3 fingerprint representations implemented in `rdkit`, all 512 bits each into one 1536 representation: Morgan, Atom, TopologicalTorsion. The fingerprint is projected onto 20 dimensions using Principal Component Analysis. The input to the model is the difference in fingerprint between two molecules, $f_A - f_B$, and the output is the whether the molecule A is more or less potent that molecule B – i.e. a classification problem. Note that this creates a balanced classification dataset.

The classifier we employ is the FastAI tabular model, a general machine learning package for processing classification problems.

Source code of our method can be found in: `https://github.com/wjm41/mpro-rank-gen/tree/main/rank_model`.

V. COMPOUND GENERATION

To generate new compounds, we: (a)introduce linker and chemotype swaps, e.g. amide to retroamide, amide to urea, swapping N-aryl groups; (b) fragment compounds along synthetically accessible bonds into building blocks, e.g. amide to carboxylic acid and amine; (c) reconnect the fragments to form a library of virtual compounds. These operations are defined using SMARTS rules.

The virtual library of compounds is then scored against the top 4 compound in the training set using the learning-to-rank framework. Compounds predicted to have higher activity is then fed into our synthetic route predictor [1, 2]. 5 molecules with <4 predicted steps were synthesised and assayed.

VI. DOCKING WORKFLOW

As a baseline comparison, we docked the training and test sets of our machine learning model against x2908 structure reported by Diamond XChem [3]. We use the "Classic OEDocking" floe v0.7.2 as implemented in the Orion 2020.3.1 Academic Stack (OpenEye Scientific). Omega was used to enumerate conformations (and expand stereochemistry) with

up to 500 conformations. `FRED` was used for docking in `HYBRID` mode using the x2908 bound ligand.

VII. RETROSPECTIVE PERFORMANCE ANALYSIS

We compare the performance of our model in predicting the pairwise ranking of compounds against the baseline model of simply training a regression model on bioactivities. As a baseline model, we trained a random forest model with the package `scikit-learn` [4], `RandomForestRegressor` function, with default hyperparameters. Likewise, for the learning-to-rank model we take the FastAI tabular model with default hyperparameters, as discussed in the main text.

---

[1] P. Schwaller, T. Laino, T. Gaudin, P. Bolgar, C. A. Hunter, C. Bekas, and A. A. Lee, ACS central science **5**, 1572 (2019).

[2] A. A. Lee, Q. Yang, V. Sresht, P. Bolgar, X. Hou, J. L. Klug-McLeod, C. R. Butler, *et al.*, Chemical Communications **55**, 12152 (2019).

[3] A. Douangamath, D. Fearon, P. Gehrtz, T. Krojer, P. Lukacik, C. D. Owen, E. Resnick, C. Strain-Damerell, A. Aimon, P. Ábrányi-Balogh, *et al.*, Nature communications **11**, 1 (2020).

[4] F. Pedregosa, G. Varoquaux, A. Gramfort, V. Michel, B. Thirion, O. Grisel, M. Blondel, P. Prettenhofer, R. Weiss, V. Dubourg, J. Vanderplas, A. Passos, D. Cournapeau, M. Brucher, M. Perrot, and E. Duchesnay, Journal of Machine Learning Research **12**, 2825 (2011).
