# Supplementary material for: Discovery of SARS-CoV-2 main protease inhibitors using a synthesis-directed de novo design model
Source: Chem Commun (Camb). 2021 May 6;57(48):5909–12. doi: 10.1039/d1cc00050k (PMC8204246; doi:10.1039/d1cc00050k)
Supplement: CC-057-D1CC00050K-s083 [file CC-057-D1CC00050K-s083.docx]

Experimental part for Compound 1 (ALP-POS-ddb41b15-4)

**Scheme:**

**Experimental part:**

1. Preparation of compound **2**

To a mixture of compound **1** (1 g, 6.57 mmol, 917.43 uL, 1 *eq*) in DCM (20 mL) was added Dess-Martin (3.34 g, 7.88 mmol, 2.44 mL, 1.2 *eq*) at 0 °C for one portion. The reaction mixture was stirred at 0 °C for 1 hr to give a yellow mixture. TLC （PE/EA 2:1） showed the reaction was complete and one major new spot was formed. The reaction mixture was poured into 30 mL sat.aq. Na_2_CO_3_ and extracted with EtOAc (40 mL*3). The combined organic phase dried over Na_2_SO_4_, filtered and concentrated to dryness in vacuum. The residue was purified by flash column (EtOAc 0% to 30% in PE) to give compound **2** (800 mg, 81% yield) as a yellow oil.

^1^H NMR (400MHz, CHLOROFORM-d) δ= 9.76 (t, J=2.4 Hz, 1H), 7.31 (t, J=7.9 Hz, 1H), 6.91 - 6.81 (m, 2H), 6.78 (d, J=1.5 Hz, 1H), 3.84 (s, 3H), 3.68 (d, J=2.4 Hz, 2H)

1. Preparation of compound **4**

To a mixture of compound **3** (500 mg, 3.92 mmol, 416.67 uL, 1 *eq*) in THF (10 mL) was added compound **2** (700 mg, 4.66 mmol, 1.19 *eq*) at 25 °C for one portion. The mixture was stirred at 25 °C for 30 min, then NaBH(OAc)_3_ (1.66 g, 7.84 mmol, 2 *eq*) was added to the mixture. The reaction mixture was stirred at 25 °C for another 2.5 hr to give a yellow mixture. The reaction mixture was poured into 10 mL brine and extracted with EtOAc (20 mL*2). The combined organic phase dried over Na_2_SO_4_, filtered and concentrated to dryness in vacuum. The residue was purified by flash column (EtOAc 0% to 10 % in PE) to give compound **4** (750 mg, 60% yield) as a colorless oil.

^1^H NMR (400MHz, CHLOROFORM-d) δ= 7.19 - 7.14 (m, 1H), 6.99 (t, J=8.1 Hz, 1H), 6.76 - 6.70 (m, 2H), 6.68 (s, 1H), 6.59 (d, J=7.8 Hz, 1H), 6.51 (t, J=2.1 Hz, 1H), 6.39 (dd, J=2.1, 8.1 Hz, 1H), 3.73 (s, 3H), 3.31 (t, J=6.9 Hz, 2H), 2.81 (t, J=6.9 Hz, 2H)

1. Preparation of compound **6**

To compound **5** (500 mg, 2.64 mmol, 1 *eq*) was added SOCl_2_ (8.20 g, 68.92 mmol, 5.00 mL, 26.08 *eq*) at 25 °C for one portion. The reaction mixture was stirred at 75 °C for 2 h to give a yellow mixture. LCMS showed compound **5** was consumed up and desired product (MeOH quenched for analysis) was formed as a major peak. The reaction mixture was concentrated to dryness in vacuum to give compound **6** (500 mg, 91% yield) as a yellow solid.

1. Preparation of compound **ALP-POS-ddb41b15-4**

To a mixture of compound **4** (550 mg, 2.10 mmol, 1 *eq*) and PYRIDINE (831.05 mg, 10.51 mmol, 848.01 uL, 5 *eq*) in DCM (12 mL) was added compound **6** (436.25 mg, 2.10 mmol, 1 *eq*) at 0 °C for one portion. The reaction mixture was stirred at 25 °C for 4 hr to give a yellow mixture. TLC （PE/EA 1:1） showed the reaction was complete. The reaction mixture was filtered and the filtrate was concentrated to dryness in vacuum. The residue was purified by prep-HPLC (Condition: water(10mM NH_4_HCO_3_)-ACN; Column: Phenomenex Gemini-NX C18 75*30 mm*3 um). The afford flows were combined and concentrated to remove most of MeCN and lyophilized to give compound **ALP-POS-ddb41b15-4** (267.7 mg, 29% yield) as a white solid.

^1^H NMR (400 MHz, METHANOL-*d*_4_) δ ppm 3.00 (br t, *J*=6.69 Hz, 2H), 3.82 (s, 3H), 4.26-4.66 (m, 2H), 6.27 (s, 1H), 6.88-6.95 (m, 4H), 7.01 (t, *J*=1.88 Hz, 1H), 7.11-7.32 (m, 6H), 7.56 (ddd, *J*=8.35, 7.04, 1.50 Hz, 1H).

^13^C NMR (400 MHz, DMSO-*d*_6_) δ ppm 33.6, 49.6, 55.4, 112.5, 114.9, 116.0, 116.5, 120.0, 121.7, 122.5, 126.1, 127.1, 128.3, 129.9, 130.9, 131.3, 133.5, 139.2, 140.4, 143.0, 146.6, 159.8, 161.1, 165.9

**THE END**

Experimental part for project Compound 2 (ALP-POS-ddb41b15-1)

**Scheme:**

**Experimental part:**

1. Preparation of compound **2**

To compound 1 (2 g, 10.57 mmol, 1 *eq*) was added SOCl_2_ (32.80 g, 275.70 mmol, 20.00 mL, 26.08 *eq*) at 25 °C for one portion. The reaction was stirred at ~80 °C for ~2 h. Then the reaction solution was cooled and concentrated in vacuum to give compound **2** (2.2 g, over 100% yield) as a yellow solid, which was direct used in the next step.

1. Preparation of compound **3**

To a mixture of LiHMDS (1 M, 19.27 mL, 4 *eq*)   in DMF (8 mL)  was added methyl 2-(3-chlorophenyl)acetate (889.25 mg, 4.82 mmol, 1 *eq*)  at 0 °C for one portion, the mixture was stirred at 0 °C for 1 h. Then compound 2 (1 g, 4.82 mmol, 1 *eq*) was added to the mixture and the reaction mixture was stirred at 25 °C for 15 h to give a yellow mixture. TLC (EA) showed the reaction was completed and one major new spot was formed. The reaction solution was poured into H_2_O (40 mL) and many solid was formed. The solid was collected, washed with water (10 mL*2) and dried in vacuum to give compound **3** (1.2 g, ~60% yield) as a yellow solid, ~85% purity by LC-MS.

1. Preparation of ALP-POS-ddb41b15-1

To a solution of compound 6 (120 mg, 1.12 mmol, 4 *eq*) in THF (2 mL) was added LiHMDS (1.1 mL, 4 eq) at RT, then stirred for ~5 mins. Compound **3** (100 mg, 0.28 mmol, 1 *eq*) at 0 °C for one portion. The reaction mixture was stirred at 60 °C for 16 h to give a yellow mixture. TLC（EA）showed the reaction was not complete and one small new spot was formed. The reaction mixture was poured into 10 mL brine and extracted with EtOAc (20 mL*3). The combined organic phase dried over Na_2_SO_4_, filtered and concentrated to dryness in vacuum. The residue was purified by prep.HPLC to give ALP-POS-ddb41b15-1 (1.8 mg) as a yellow solid.

^1^H NMR (400 MHz, CDCl3) δ ppm 2.01 (s, 3H), 3.94 (s, 1H), 6.37 (s, 1H), 6.78 (m, 1H), 7.06 (m, 1H), 7.13-7.16 (m, 1H), 7.21 (m, 1H), 7.48-7.45 (m, 2H), 7.78 (m, 2H), 7.8 (m, 1H), 8.25 (m, 1H), 8.27 (m, 1H), 8.95 (s, 1H), 10.63 (brs, 1H), 14.8 (brs, 1H).

1. Preparation of compound **7**

To a mixture of compound 6 (2 g, 11.72 mmol, 1 eq)  , t-BuOH (8.69 g, 117.24 mmol, 11.21 mL, 10 eq)  and PYRIDINE (4.64 g, 58.62 mmol, 4.73 mL, 5 eq)  in DCM (20 mL)  was added POCl_3_ (3.60 g, 23.45 mmol, 2.18 mL, 2 eq)  at 0 °C in portions. The reaction mixture was stirred at 25 °C for 2 h to give a yellow mixture. TLC (PE/EA 5:1) showed one major new spot was formed. The reaction solution was diluted with H_2_O (50 mL) and extracted with DCM (50 mL). The organic layer was dried and concentrated in vacuum to give the residue, which was purified by ComBi flash (PE/EA 0-20%) to give compound **7** (2 g, 75% yield) as yellow oil.

^1^H NMR (400MHz, CHLOROFORM-d) δ= 7.22 - 7.16 (m, 3H), 7.10 - 7.06 (m, 1H), 3.45 - 3.41 (m, 2H), 1.37 (s, 9H)

1. Preparation of compound **8**

To a mixture of LiHMDS (1 M, 26.01 mL, 3 *eq*)   in DMF (5 mL) was added compound 7 (1.97 g, 8.67 mmol, 1 *eq*)  at 0 °C for one portion, the mixture was stirred at 0 °C for 1 h. Then compound 2 (1.8 g, 8.67 mmol, 1 *eq*) was added to the mixture and the reaction mixture was stirred at 25 °C for 15 h to give a yellow mixture. TLC (EA) showed the reaction was completed and one major new spot was formed. The reaction solution was poured into H_2_O (40 mL) and many solid was formed. The solid was collected, washed with water (10 mL*2) and dried in vacuum to give compound **8** (1.7 g, ~49% yield) as a yellow solid, ~70% purity by 1HNMR.

^1^H NMR (400MHz, DMSO-d 6) δ= 11.40 - 11.11 (m, 1H), 7.67 - 7.53

(m, 1H), 7.46 (br d, J=8.5 Hz, 1H), 7.34 - 7.24 (m, 2H), 7.23 (s, 1H), 7.13 - 7.07 (m, 1H), 7.06 - 6.90 (m, 2H), 6.75 (br d, J=7.8 Hz, 1H), 5.88 - 5.61 (m, 1H), 1.33 - 1.20 (m, 9H)

1. Preparation of compound **9**

 To a solution of compound **8** (100 mg, 251.35 umol, 1 *eq*) in DCM (5 mL) was added TFA (1 mL). The reaction was stirred at 25 °C for ~16 h to give a yellow mixture. LC-MS showed the reaction was a bit messy, no SM was left and ~26% of d.p was detected.

1. Preparation of compound **9**

To a mixture of methyl compound 3 (100 mg, 281.08 umol, 1 *eq*)  in THF (1 mL)  , MeOH (1 mL)  and H_2_O (0.5 mL)  was added NaOH (67.45 mg, 1.69 mmol, 6 *eq*)  at 25 °C. The reaction solution was stirred at 25 °C for 16 h. LC-MS showed no SM was left, but no d.p was detected and ~49% of de-COOH was detected.

**THE END**

Experimental part for Compound 3 (ALP-POS-ddb41b15-3)

**Scheme:**

**Experimental part:**

1. Preparation of compound **2**

To a mixture of compound **1** (2 g, 16.79 mmol, 1 *eq*) in DMF (20 mL) was added NaH (1.48 g, 36.94 mmol, 60% purity, 2.2 *eq*) at 0 °C in portions, the mixture was stirred at 0 °C for 0.5 hr. Then, a solution of MeI (5.24 g, 36.94 mmol, 2.30 mL, 2.2 *eq*) in DMF (10 mL) was added to the mixture at 0 °C for one portion and the reaction mixture was stirred at 25 °C for another 2 h to give a yellow mixture. TLC showed compound **1** (EtOAc:PE 1 : 1, Rf ~ 0.3) was consumed up and compound **2** (Rf ~ 0.5) was formed. To the reaction mixture was added 50 mL ice water at 0 °C in portions. Then, the mixture was extracted with EtOAc (60 mL*3). The combined organic phase dried over Na_2_SO_4_, filtered and concentrated to dryness in vacuum. The residue was purified by flash column (EtOAc 0% to 60% in PE) to give compound **2** (1.3 g, 52% yield) as a yellow solid.

^1^H NMR (400MHz, CHLOROFORM-d) δ ppm 8.29 (d, J=3.0 Hz, 1H), 8.20 (s, 1H), 7.11 (dd, J=1.6, 2.8 Hz, 1H), 3.05 (s, 6H)

1. Preparation of compound **3**

To a mixture of compound 2 (1.2 g, 8.15 mmol, 1 *eq*) in NH_3_**^.^**H_2_O (9.10 g, 64.92 mmol, 10 mL, 25% purity, 7.96 *eq*) and MeOH (15 mL) was added Raney-Ni (250 mg, 2.92 mmol, 3.58e-1 *eq*) at 25 °C for one portion. The reaction mixture was stirred at 25 °C under H_2_ (50 Psi) for 4 hr to give a yellow mixture. TLC （PE/EA 1：1, Rf~0.5） showed the reaction was completed. The mixture was filtered and the filtrate was concentrated to dryness in vacuum to give compound **3** (900 mg, 73% yield) as a yellow solid.

1H NMR (400MHz, DMSO-d_6_) δ ppm 8.01 - 7.76 (m, 2H), 7.07 (br s, 1H), 3.87 - 3.53 (m, 2H), 2.92 (s, 6H)

1. Preparation of compound **4**

To a mixture of compound **8** (400 mg, 1.24 mmol, 1 *eq*) in THF (8 mL), MeOH (4 mL) and H_2_O (2 mL) was added LiOH**^.^**H_2_O (207.65 mg, 4.95 mmol, 4 *eq*) at 25 °C for one portion. The reaction mixture was stirred at 25 °C for 4 h to give a yellow mixture. TLC （PE/EA 1：1, Rf~0.6） showed the reaction was completed The pH of the reaction mixture was adjusted to 4-5 by 1*N* aq.HCl and extracted with EtOAc (20 mL*3). The combined organic phase dried over Na_2_SO_4_, filtered and concentrated to dryness in vacuum to give compound **4** (350 mg, 91% yield) as a white solid.

1H NMR (400MHz, DMSO-d_6_) δ ppm 8.27 - 8.19 (m, 1H), 7.67 - 7.52 (m, 2H), 7.38 - 7.31 (m, 1H), 7.23 (d, J=8.6 Hz, 2H), 7.11 (s, 1H), 6.95 - 6.90 (m, 2H), 5.54 (br s, 2H), 3.75 (s, 3H)

1. Preparation of compound **5**

To a mixture of compound **4** (1.02 g, 3.31 mmol, 1 *eq*) in DMF (2.5 mL) was added DIEA (1.71 g, 13.23 mmol, 2.30 mL, 4 *eq*), HATU (1.89 g, 4.96 mmol, 1.5 *eq*) and compound **3** (500 mg, 3.31 mmol, 1 *eq*) at 25 °C for one portion. The reaction mixture was stirred at 25 °C for 16 h to give a yellow mixture. TLC showed compound **3** (PE : EA 0:1, Rf~0.4) was consumed up and one major spot was formed. The mixture was poured into brine (30 mL) and extracted with EtOAc (35 mL*3). The combined organic phase dried over Na_2_SO_4_, filtered and concentrated to dryness in vacuum. The residue was purified by flash column (MeOH 0% to 5% in DCM) to give compound **5** (1.03 g, 463% yield) as a yellow solid.

^1^H NMR (400MHz, DMSO-d_6_) δ ppm 9.38 (t, J=5.9 Hz, 1H), 8.06 (d, J=2.8 Hz, 1H), 7.94 (s, 1H), 7.76 (dd, J=1.1, 8.1 Hz, 1H), 7.60 - 7.49 (m, 2H), 7.25 (t, J=7.4 Hz, 1H), 7.20 - 7.15 (m, 3H), 6.88 (d, J=8.8 Hz, 2H), 6.78 (s, 1H), 5.49 (br s, 2H), 4.51 (d, J=5.9 Hz, 2H), 3.70 (s, 3H), 2.96 (s, 6H)

1. Preparation of compound **6**

To a mixture of compound **5** (800 mg, 1.81 mmol, 1 *eq*) and compound **7** (862.19 mg, 3.62 mmol, 446.73 uL, 2 *eq*) in DMSO (24 mL) was added CuI (172.15 mg, 903.94 umol, 0.5 *eq*), K_2_CO_3_ (499.72 mg, 3.62 mmol, 2 *eq*) and 1,10-phenanthroline (162.89 mg, 903.94 umol, 0.5 *eq*) at 25 °C for one portion. The reaction mixture was stirred at 130 °C under N_2_ (15 Psi) for 16 hr to give a yellow mixture. The reaction mixture was poured into 50 mL brine and extracted with EtOAc (50 mL*4). The combined organic phase washed with water (80 mL*3) dried over Na_2_SO_4_, filtered and concentrated to dryness in vacuum. The residue was purified by flash column (EtOAc 0% to 100% in PE) to give compound **6** (350 mg, 34% yield) as a yellow oil.

1H NMR (400MHz, DMSO-d_6_) δ ppm 8.03 (d, J=2.6 Hz, 1H), 7.84 (s, 1H), 7.71 (d, J=7.0 Hz, 1H), 7.54 - 7.48 (m, 1H), 7.38 - 7.32 (m, 2H), 7.28 (t, J=7.8 Hz, 1H), 7.25 - 7.21 (m, 1H), 7.15 (t, J=8.0 Hz, 1H), 7.18 - 7.13 (m, 1H), 7.04 - 6.97 (m, 2H), 6.93 - 6.88 (m, 2H), 6.85 - 6.79 (m, 2H), 6.75 (s, 1H), 5.37 (br s, 2H), 5.16 (br s, 2H), 3.69 (s, 3H), 2.92 (s, 6H)

1. Preparation of compound **ALP-POS-ddb41b15-3**

To compound **6** (350 mg, 632.85 umol, 1 *eq*) was added TFA (8 mL) at 25 °C for one portion. The reaction mixture was stirred at 95 °C for 16 hr to give a yellow mixture. TLC showed part of compound **6** (PE/EA 0:1, Rf = 0.4) was remained and a major new spot was formed. The pH of the reaction mixture was adjusted to 7 by sat. aq.NaHCO_3_. The mixture was extracted with EtOAc (20 mL*3). The combined organic phase dried over Na_2_SO_4_, filtered and concentrated to dryness under reduced pressure. The residue was purified by prep-HPLC (column: Phenomenex Gemini-NX C18 75*30 mm*3 um;mobile phase: [water(10 mM NH_4_HCO_3_)-ACN];B%: 21%-61%,11min). The afford flows were combined and concentrated to remove most of MeCN and lyophilized to give compound **ALP-POS-ddb41b15-3** (75.6 mg, 158.34 umol, 25.02% yield, 90.667% purity) as a white solid.

^1^H NMR (400 MHz, DMSO-*d*_6_) δ ppm 2.92 (s, 6 H) 5.13 (br s, 2 H) 6.49 (s, 1 H) 6.93 - 7.00 (m, 2 H) 7.12 - 7.28 (m, 4 H) 7.36 (s, 1 H) 7.51 (t, *J*=7.38 Hz, 1 H) 7.63 (d, *J*=8.00 Hz, 1 H) 7.82 (s, 1 H) 8.02 (d, *J*=2.75 Hz, 1 H) 11.74 (br s, 1 H)

^13^C NMR (400 MHz, DMSO-*d*_6_) 41.0, 50.0, 116.2, 116.8, 118.5, 120.7, 122.7, 125.8, 127.3, 128.4, 130.9, 131.4, 132.1, 133.4, 134.5, 137.3, 139.3, 142.4, 146.1, 146.3, 161.1, 166.4

**THE END**

**Experimental part for Compound 4 (ALP-POS-ddb41b15-9)**

**Scheme**

***Experimental:***

1. ***Preparation procedure of compound 4***.

To a solution of compound 1 (1 g, 8.46 mmol) in pyridine (8 mL) was added Et_3_N (1.71 g, 16.93 mmol, 2.36 mL), then compound 3 (2.89 g, 16.93 mmol, 2.31 mL) was dropwised at 0 °C under N_2_. The mixture was stirred at 20 °C for 14 h. Then aq.NaOH (7 M, 1.81 mL) was added to the above solution, the resulting solution was stirred at 100 °C for 1 h, then cooled to RT. LC-MS showed compound 1 was consumed completely and ~26% of desired compound was detected. The reaction mixture was quenched by addition ice-water 20 mL, and then diluted with H_2_O (30 mL) and extracted with EA 100 mL (50 mL * 2). The combined organic layers were washed with brine (50 mL) dried over Na_2_SO_4_, filtered and concentrated under reduced pressure to give a residue. The residue was purified by flash silica gel chromatography (PE/EA 0~40%) to give compound 4 (900 mg, 42% yield) as a yellow solid.

^1^H NMR (400MHz, CHLOROFORM-d) δ ppm 10.40 (br s, 1H), 8.33 (d, J=7.8 Hz, 1H), 8.08 (d, J=1.9 Hz, 1H), 7.90 (d, J=8.1 Hz, 1H), 7.55 - 7.49 (m, 1H), 7.44 - 7.28 (m, 3H), 7.15 (dd, J=2.6, 8.3 Hz, 1H), 3.85 (s, 3H)

1. ***Preparation procedure of compound 5A***

A mixture of compound 4 (200 mg, 792.81 umol) and NH_2_OH.HCl (110.18 mg, 1.59 mmol) in pyridine (4 mL), and then the mixture was stirred at 70 °C for 2 h. TLC (EtOAc) showed the reaction was completed. The reaction mixture was concentrated under reduced pressure to remove pyridine. The residue was diluted with H_2_O (10 mL) and extracted with EA (10 mL * 3). The combined organic layers were washed with brine (10 mL), dried over Na_2_SO_4_, filtered and concentrated under reduced pressure to give a residue, which was purified by flash silica gel chromatography (DCM/MeOH 15:1 to 10:1)to give compound 5A (180 mg, 90% yield) as a white solid.

1H NMR (400MHz, METHANOL-d_4_) δ ppm 7.71 - 7.62 (m, 1H), 7.60 - 7.51 (m, 1H), 7.44 - 6.98 (m, 6H), 3.82 (d, J=7.3 Hz, 3H)

1. ***Preparation procedure of compound 5***.

To a solution of compound 5A (180 mg, 0.67 mmol) in THF (5 mL) was added dropwise 1M BH_3_.THF (2.7 mL, 2.7 mmol) at 0 °C, then the resulting solution was stirred at RT overnight. TLC (DCM/MeOH 10:1, a few drops NH_3_.H_2_O) showed the reaction was complete and a major new spot was detected. The reaction solution was quenched with H_2_O (10 mL) and extracted with DCM (10 mL * 3). The combined organic layers were dried over Na_2_SO_4_, filtered and concentrated under reduced pressure to give a residue, which was purified by flash silica gel chromatography (DCM/MeOH 10:1 to 5:1)to give compound 5 (~100 mg, 90% yield) as a colorless gum, which was directly used in the next step..

1. ***Preparation procedure of the final target***.

To a solution of compound 5 (65 mg, 256.61 umol) in DCM (1 mL) and DIEA (74.20 mg, 574.11 umol, 0.1 mL) was added compound 6 (50 mg, 264.50 umol). The mixture was stirred at 25 °C for 2 h. LC-MS showed reactant 1 was consumed completely and one major desired peak was detected. The reaction mixture was diluted with H_2_O 10 mL and extracted with DCM (10 mL * 3). The combined organic layers were dried over Na_2_SO_4_, filtered and concentrated under reduced pressure to give a residue. The residue was diluted with MeOH 2 mL and was purified by prep-HPLC (FA condition; Column: Phenomenex luna C18 100*40mm*3 um; Condition: water (0.225%FA)-ACN; Begin B:20; End B:50; Gradient Time(min):10; 100%B Hold Time(min):2) to give ALP-POS-ddb41b15-9 (16.85 mg, 15.8% yield) as a white solid.

^1^H NMR (400MHz, CHLOROFORM-d) δ ppm 7.87 (br d, J=7.5 Hz, 1H), 7.65 - 7.34 (m, 2H), 7.27 - 7.09 (m, 6H), 6.91 - 6.84 (m, 2H), 6.82 - 6.71 (m, 1H), 6.42 (d, J=7.5 Hz, 1H), 3.67 - 3.63 (m, 3H), 3.60 (s, 2H)

^13^C NMR (400MHz, DMSO-d_6_) δ ppm 41.7, 51.9, 55.5, 111.8, 113.3, 119.0, 119.9, 121.7, 122.6, 126.8, 128.3, 129.4, 130.0, 130.4, 133.2, 139.2, 141.8, 154.3, 159.7, 169.7

Experimental part for project Compound 5 (ALP-POS-ddb41b15-8)

**Scheme:**

**Experimental part:**

1. Preparation of compound **2**

To a mixture of compound **1** (1 g, 8.46 mmol, 1 *eq*) and Et_3_N (1.71 g, 16.93 mmol, 2.36 mL, 2 *eq*) in pyridine (8 mL) was added compound **2** (2.96 g, 16.93 mmol, 2.16 mL, 2 *eq*) at 0 °C in portions, the reaction was stirred at 25 °C for 16 h. Then aq.NaOH (7 M, 1.81 mL, 1.5 *eq*) was added to the mixture and the reaction was stirred at 100 °C for another 1 h. TLC （PE/EA 1:1） showed the reaction was complete and one major new spot was formed. The reaction mixture cooled and poured into 40 mL brine and extracted with EtOAc (20 mL*3). The combined organic phase dried over Na_2_SO_4_, filtered and concentrated to dryness in vacuum. The residue was purified by flash column (EtOAc 0% to 50% in PE) to give compound **3** (800 mg, 36% yield) as a yellow solid.

^1^H NMR (400MHz, DMSO-d_6_) δ ppm 13.59 (brs,1H),8.60 (t, J=1.8Hz, 1H), 8.47 (d, J=7.9 Hz, 1H), 7.90 (br s, 1H), 7.82 - 7.78 (m, 1H), 7.66 (t, J=7.9 Hz, 2H), 7.40 (br d, J=19.4 Hz, 2H)

1. Preparation of compound **4**

To a mixture of compound **3** (550 mg, 2.14 mmol, 1 *eq*) in DMF (10 mL) was added K_2_CO_3_ (444.20 mg, 3.21 mmol, 1.5 *eq*), the mixture was stirred at 25 °C for 0.5 h, then MeI (456.19 mg, 3.21 mmol, 200.08 uL, 1.5 *eq*) was added to the mixture and the reaction was stirred at 80 °C for another 2.5 h to give a yellow mixture. TLC （EA） showed the reaction was completed and one major new spot was formed. The reaction mixture was poured into 10 mL brine and extracted with EtOAc (10 mL*3). The combined organic phase washed with water (20 mL*3) dried over Na_2_SO_4_, filtered and concentrated to dryness in vacuum to give compound **4** (450 mg, 70% yield) as a yellow oil.

^1^H NMR (400MHz, DMSO-d_6_) δ ppm 8.30 (t, J=1.8 Hz, 1H), 8.22 (td, J=1.3, 7.8 Hz, 1H), 7.91 - 7.87 (m, 1H), 7.83 - 7.77 (m, 2H), 7.68 - 7.62 (m, 1H), 7.51 (ddd, J=1.1, 7.1, 8.3 Hz, 1H), 7.39 (ddd, J=1.1, 7.1, 8.2 Hz, 1H), 4.12 (s, 3H)

1. Preparation of compound 5

To a mixture of hydroxylamine;hydrochloride (179.69 mg, 2.59 mmol, 2 *eq*) in pyridine (8 mL) was added compound **4** (450 mg, 1.29 mmol, 1 *eq*) at 0 °C for one portion. The reaction mixture was stirred at 70 °C for 4 h to give a yellow mixture. TLC （EA） showed the reaction was completed and one major new spot was formed. The reaction mixture was poured into 15 mL brine and extracted with EtOAc (20 mL*4). The combined organic phase dried over Na_2_SO_4_, filtered and concentrated to dryness in vacuum. The residue was purified by flash column (EtOAc 0% to 40% in PE) to give compound **5** (400 mg, 83% yield) as a white solid.

1H NMR (400MHz, DMSO-d_6_) δ ppm 12.53 (s, 1H), 12.35 (s, 1H), 7.73 - 7.62 (m, 2H), 7.53 - 7.44 (m, 4H), 7.39 - 7.26 (m, 2H), 3.69 (s, 3H)

1. Preparation of compound **6**

To a mixture of compound **5** (300 mg, 1.05 mmol, 1 *eq*) in THF (1 mL) was added BH_3_**^.^**THF (1 M, 15.00 mL, 14.29 *eq*) at 0 °C for one portion. The reaction mixture was stirred at 40 °C for ~6 h. ~5 mL MeOH was added to the mixture at 0 °C in portions. Then diluted with H_2_O (10 mL), the mixture was extracted with EtOAc (20 mL*4). The combined organic phase dried over Na_2_SO_4_, filtered and concentrated to dryness in vacuum. The residue was purified by flash column (PE/EA 1:1 to 0:1) to give compound **6** (180 mg, 45% yield) as yellow oil.

1. Preparation of compound ALP-POS-ddb41b15-8

To a mixture of compound **7** (96.70 mg, 551.99 umol, 1 *eq*), HATU (314.82 mg, 827.98 umol, 1.5 *eq*) and DIEA (285.36 mg, 2.21 mmol, 384.59 uL, 4 *eq*) in DMF (5 mL) was added compound **6** (150 mg, 551.99 umol, 1 *eq*). The reaction mixture was stirred at 25 °C for 16 h. The reaction was diluted with H_2_O (~20 mL) and extracted with EA (10 mL *3). The organic layer was dried and concentrated. The residue was purified by prep-HPLC (column: Phenomenex Gemini-NX C18 75*30 mm*3 um; mobile phase: [water(10 mM NH_4_HCO_3_)-ACN];B%: 35%-75%,14 min). The afford flows were combined and concentrated to remove most of MeCN and lyophilized to give compound **ALP-POS-ddb41b15-8** (76.40 mg, 32% yield) as a yellow solid.

^1^H NMR (400 MHz, DMSO-*d*_6_) δ ppm 3.75 (s, 5H), 6.21 (s, 1H), 6.56 (d, *J*=8.13 Hz, 1H), 6.89-6.96 (m, 1H), 6.97-7.04 (m, 1H), 7.19-7.34 (m, 3H), 7.36-7.44 (m, 4H), 7.52-7.59 (m, 2H), 7.64 (d, *J*=7.50 Hz, 1H), 9.35 (d, *J*=8.00 Hz, 1H), 10.97 (s, 1H).

^13^C NMR (400 MHz, DMSO-*d*_6_) δ ppm 30.3, 35.5, 49.4, 100.4, 110.7, 111.3, 119.1, 119.4, 119.7, 120.8, 122.2, 122.9, 127.0, 128.1, 128.5, 130.6, 133.4, 134.0, 136.2, 136.5, 141.9, 142.1, 152.8, 153.8, 168.9.

**THE END**
